# Mixing tree species associated with arbuscular or ectotrophic mycorrhizae reveals dual mycorrhization and interactive effects on the fungal partners

**DOI:** 10.1002/ece3.7437

**Published:** 2021-04-02

**Authors:** Heike Heklau, Nicole Schindler, François Buscot, Nico Eisenhauer, Olga Ferlian, Luis D. Prada Salcedo, Helge Bruelheide

**Affiliations:** ^1^ Institute of Biology/Geobotany and Botanical Garden Martin Luther University Halle‐Wittenberg Halle (Saale) Germany; ^2^ Department of Soil Ecology Helmholtz Centre for Environmental Research – UFZ Halle (Saale) Germany; ^3^ German Centre for Integrative Biodiversity Research (iDiv) Halle‐Jena‐Leipzig Leipzig Germany; ^4^ Institute of Biology Leipzig University Leipzig Germany

**Keywords:** arbuscular mycorrhiza, biodiversity‐ecosystem functioning experiment, chernozem, dual mycorrhization, ectomycorrhiza, multi‐trophic interaction, next‐generation sequencing

## Abstract

1. Recent studies found that the majority of shrub and tree species are associated with both arbuscular mycorrhizal (AM) and ectomycorrhizal (EM) fungi. However, our knowledge on how different mycorrhizal types interact with each other is still limited. We asked whether the combination of hosts with a preferred association with either AM or EM fungi increases the host tree roots’ mycorrhization rate and affects AM and EM fungal richness and community composition.
2. We established a tree diversity experiment, where five tree species of each of the two mycorrhiza types were planted in monocultures, two‐species and four‐species mixtures. We applied morphological assessment to estimate mycorrhization rates and next‐generation molecular sequencing to quantify mycobiont richness.
3. Both the morphological and molecular assessment revealed dual‐mycorrhizal colonization in 79% and 100% of the samples, respectively. OTU community composition strongly differed between AM and EM trees. While host tree species richness did not affect mycorrhization rates, we observed significant effects of mixing AM‐ and EM‐associated hosts in AM mycorrhization rate. Glomeromycota richness was larger in monotypic AM tree combinations than in AM‐EM mixtures, pointing to a dilution or suppression effect of AM by EM trees. We found a strong match between morphological quantification of AM mycorrhization rate and Glomeromycota richness.
4. *Synthesis*. We provide evidence that the combination of hosts differing in their preferred mycorrhiza association affects the host's fungal community composition, thus revealing important biotic interactions among trees and their associated fungi.

## Graphical abstract

In a tree diversity experiment where different mycorrhizal host species were planted in monospecific stands and in mixtures, we found that combining arbuscular or ectotrophic mycorrhizae affect mycorrhization rates and richness of AM and EM fungi. Glomeromycota richness was larger in monotypic AM tree combinations than in mixtures of AM‐EM host trees, pointing to a suppression effect of AM fungi by the presence of EM trees.

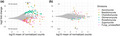

## 1 INTRODUCTION

The majority of terrestrial plant species benefit from symbiosis with mycorrhizal fungi (Smith & Read, 2009). It is well known that mycorrhizal fungi improve the mineral plant nutrition, provide protection against abiotic stress and pathogens, and mediate the communication between plants (Smith & Read, 2009; Tedersoo & Bahram, 2019). In turn, the host plants provide carbon to the root‐associated fungi. There are two main mycorrhizal types in trees: arbuscular mycorrhiza (AM), which is exclusively associated with Glomeromycota and ectomycorrhiza (EM), which forms associations mainly with Basidiomycota, but also with Ascomycota. EM trees prevail in temperate forests. EM fungi provide 34.1% of all fungal taxa in the northern temperate deciduous forests, but less (11.9%) in grass‐ and shrublands (Tedersoo et al., 2014). In contrast, AM fungi occur in 74% of angiosperm species (Brundrett, 2009), and in particular, dominate in relatively nutrient‐rich (e.g., arable soil) or phosphorous‐limited habitats (Tedersoo & Bahram, 2019). The two mycorrhizal types differ in nutrient acquisition strategies. While EM fungi have the ability to utilize organic nutrient sources (Dickie et al., 2014), AM fungi cannot (Read & Perez‐Moreno, 2003). EM fungi access organic P, which accumulates in older soil, via excretion of phosphatases. Due to the large hyphae system, EM fungi scavenge more effectively and at further distances from the host roots compared to AM (Teste et al., 2020). In contrast, AM fungi mobilize inorganic compounds.

While double symbioses with both AM and EM have been documented for some time (Allen et al., 1999; Frioni et al., 1999; Read & Haselwandter, 1981), it has been realized recently that many plant species are associated with both AM and EM, either simultaneously within the same root system or at different life stages or in different environments. In their review on plants that are associated both with AM and EM, Teste et al., (2020) counted 238 plant species with dual mycorrhization, belonging to 89 plant genera and 32 families. While many fungal taxa are found in both AM and EM plant species, they often differ in their relevant amounts, resulting in differences in the presence of particular taxa (Teste et al., 2020) and in fungal community composition (Christ et al., 2011). Teste et al., (2020) suggest that in such cases the preference for a mycorrhizal type depends on a wide range of abiotic and biotic factors, including soil moisture and nutrient availability. When growing two dual‐mycorrhizal tree species along a dune chronosequence in southwestern Australia, Albornoz et al., (2016) found the shift from AM to EM root colonization to be best explained by increasing soil age. However, it is still unclear how the mycorrhization rate of AM and EM depends on the biotic context, in particular on the presence of host species favoring either type of mycorrhiza.

There is strong evidence that host plant diversity affects soil microbial diversity (Steinauer et al., 2016). The positive effects of plant diversity operate through increases carbon inputs into the microbial community in the rhizosphere (Eisenhauer et al., 2017), resulting in both increased microbial activity and carbon storage (Lange et al., 2015). This positive relationship also applies in particular to mycorrhiza, seen in the positive relationship between plant richness and richness of AM in grasslands (Hiiesalu et al., 2014). Such host‐mycorrhiza relationships have been demonstrated also for trees. In young subtropical Chinese forest plantations with one to 16 different tree species, Weißbecker et al., (2019) showed that plots with higher tree diversity supported higher fungal species diversity. Similarly, in two forest diversity experiments in temperate Europe, richness of soil fungal groups in Estonia and EM fungi in Finland was positively correlated with tree species richness (Tedersoo et al., 2016).

Mechanistically, the positive effects of tree richness on mycobiont richness are often explained by complementary characteristics of the different tree species involved. So far, not much is known about how different host species affect their fungal partners. In tree species, one main difference is the preferred type of mycorrhizal partner, which is either AM or EM, and to which in the following we refer to as AM and EM tree species. Thus, the strongest tree richness effects would be expected when trees with these two symbiosis types grow together. Note that mixing tree species in this way in a community does not necessarily imply that AM trees also become associated with EM fungi and vice versa. In the following, we refer to such mixed tree communities as “mix‐type.” Knowledge on how much combining such different host species explains species richness of their associated fungi would be key for advancing our understanding of the underlying mechanisms.

Mycorrhizae can either be studied morphologically (e.g., Baltruschat et al., 2019; Brundrett & Tedersoo, 2020; Kottke & Oberwinkler, 1986; Rumberger et al., 2004) or by meta‐barcoding (e.g., Francioli et al., 2016). Both approaches have advantages and disadvantages. In contrast to EM fungi, for which morphotyping is well established (e.g., Agerer, 1997–2001, 2001; Jakucs & Erős‐Honti, 2008), a taxonomic identification of AM fungi is only possible at higher taxonomic levels (Gai et al., 2012). However, morphological examinations allow a relatively precise assessment of the amount of hyphae/arbuscules in the host's roots (e.g., Trouvelot et al., 1986). In contrast, meta‐barcoding provides a deep taxonomic resolution but quantification of the degree of mycorrhization is often difficult. However, reads cannot be simply taken as a measure of relative abundance because of primer design and the bioinformatics procedures involved in the data analysis (Ihrmark et al., 2012). In addition, we cannot exclude contamination (fungi identified in the sample which might not have a symbiotic relationship with the host). Thus, also often saprotrophic fungi are identified by meta‐barcoding in root tissues.

To address the question whether tree richness or the mixing of AM and EM tree species results in higher effects at the ecosystem level, the tree diversity experiment “MyDiv” was established (Ferlian et al., 2018). Here tree diversity (one, two, and four tree species) and mix‐type (either AM/EM trees or AM + EM combinations) were manipulated independently from each other. We made use of this experiment to study how these two factors affect mycorrhization rates and richness of AM and EM fungi. Overall, the objective of our study was to quantify mycorrhization rates both with morphological and molecular approaches in experimental tree communities of different tree species richness and types of preferred mycorrhizal association.

In particular, we tested the hypothesis that (H1) EM and AM trees have different and type‐specific effects on mycorrhization rates and fungal species richness. Although we expect that, in principle, AM and EM host trees show dual mycorrhization, differences should be reflected both in the morphological and by meta‐barcoding assessment. Furthermore, we hypothesized that (H2) mycorrhization rates and fungal richness respond positively to tree species diversity and (H3) to mixing tree species with a preference of different mycorrhiza types (mix‐type). Finally, we tested the hypothesis (H4) that the morphological assessment of mycorrhization rates shows a correspondence with the results obtained with the molecular approach. In particular, we expected that the amount of active ectomycorrhizal root tips was correlated with richness of Basidiomycota and the frequency/intensity of arbuscular mycorrhiza in the root system with richness of Glomeromycota.

## 2 MATERIAL AND METHODS

### 2.1 Site

The experimental site is located in Saxony‐Anhalt, Central Germany, southwest of Halle (51°23’ N,11°53’ E) at 114–116 m a. s. l. at the Bad Lauchstädt Experimental Research Station of the Helmholtz Centre for Environmental Research–UFZ (Ferlian et al., 2018). The climate is characterized by a mean annual precipitation of 484 mm and a mean annual temperature of 8.8°C. The soil is a Haplic Chernozem (Altermann et al., 2005). Until 2012, this site had been used for agriculture at which point it was converted to grassland for two years before it was ploughed to prepare the site for planting the trees in March 2015 (Ferlian et al., 2018).

### 2.2 Experimental design

In the experiment, 80 plots (plot size 11 × 11 m) were organized in two blocks (for details see Ferlian et al., 2018) and 140 about 2‐ to 3‐year‐old tree individuals were planted per plot in 2015. The pool consisted of ten tree species, each five of them predominantly associated with either AM or EM. The predominant mycorrhiza type (EM or AM) of each sample tree species was assessed according to the relevant literature (Table 1). Tree species of these two main mycorrhizal types (Myc_Type) were planted in monocultures, two‐species and four‐species mixtures, thus forming a tree species richness gradient. Additionally, the two‐species and four‐species mixtures contained only AM trees, only EM trees or AM and EM trees in mixture. Thus, each plot could have two states of mycorrhiza mixture type (Mix_Type), either monotypic (AM or EM) or mixed (AM and EM trees growing together).

**TABLE 1:** Species and their abbreviated names used in the study

Abbreviation	Species	Family	Myc_Type	References	ECT [%]		AM F [%]		AM M [%]		AM A [%]	
Ac	*Acer pseudoplatanus* L.	Sapindaceae	AM	(Harley & Harley, 1987, 1990; Lang et al., 2011; Pirazzi et al., 1999; Ruckli et al., 2014; Weber & Claus, 2000)	56.15	a	87.83	a	8.86	ac	0.29	b
Ae	*Aesculus hippocastanum* L.	Sapindaceae	AM	(Bainard et al., 2010; Harley & Harley, 1987; Karliński et al., 2014)	62.64	a	81.43	ab	9.04	ac	0.16	ab
Fr	*Fraxinus excelsior* L.	Oleaceae	AM	(Beyer et al., 2013; Cesarz et al., 2013; Harley & Harley, 1987; Kubisch et al., 2016; Pirazzi et al., 1999; Weber & Claus, 2000)	45.20	a	96.11	a	22.44	a	0.18	ab
Pr	*Prunus avium* L.	Rosaceae	AM	(Aka‐Kacar et al., 2010; Harley & Harley, 1987; Pirazzi et al., 1999)	43.10	a	85.98	a	13.07	ab	1.82	a
So	*Sorbus aucuparia* L.	Rosaceae	AM	(Harley & Harley, 1987; Sýkorová et al., 2016)	35.83	a	77.78	ab	4.58	ac	0.29	ab
Be	*Betula pendula* Roth.	Betulaceae	EM	(Brun et al., 1995; Brundrett, 2009; Cuvelier, 1990; Harley & Harley, 1987, 1990; Wright et al., 2005)	41.78	a	21.30	c	5.02	bc	1.21	ab
Ca	*Carpinus betulus*L.	Betulaceae	EM	(Brundrett, 2009; Harley & Harley, 1987, 1990; Lang et al., 2011; Rewald et al., 2014; Selosse et al., 2002)	47.94	a	30.28	c	3.19	c	0.07	b
Fa	*Fagus sylvatica L*.	Fagaceae	EM	(Beyer et al., 2013; Brundrett, 2009; Harley & Harley, 1987, 1990; Selosse et al., 2002)	43.31	a	40.49	bc	3.80	c	0.88	b
Qu	*Quercus petraea* (Matt.) Liebl.	Fagaceae	EM	(Bakker et al., 2000; Brundrett, 2009; Harley & Harley, 1987, 1990; Urban et al., 2008)	45.61	a	17.22	c	0.37	c	0	b
Ti	*Tilia platyphyllos*Scop.	Malvaceae	EM	(Brundrett, 2009; Harley & Harley, 1987, 1990; Lang et al., 2011; Sisti et al., 2003)	34.34	a	42.11	bc	3.28	bc	0.04	b

Myc_Type: The tree species’ preferred type of mycorrhiza (AM, arbuscular mycorrhiza; EM ectomycorrhiza). References: Studies on which the preferred mycorrhiza type was based. ECT: Frequency of active ectomycorrhizal root tips. AM F: Frequency of arbuscular mycorrhiza in the root system. AM M: Intensity of the arbuscular mycorrhizal colonization in the root system. AM A: Arbuscular abundance in the root system. Small letters show significant differences between species according to a Tukey post hoc test

### 2.3 Sampling

The sampling of fine roots of all ten tree species was carried out at the beginning of December 2017. In total, we took 120 root samples (20 samples each of *Acer pseudoplatanus* and *Carpinus betulus*, 19 each of *Fagus sylvatica* and *Prunus avium*, and eight each of *Betula pendula* and *Tilia platyphyllos*, seven each of *Aesculus hippocastanum* and *Sorbus aucuparia* and six each of *Fraxinus excelsior* and *Quercus petraea*). For the distribution of samples by tree species, their preferred mycorrhiza type (AM, EM) and diversity level of the plots see Appendix Table S1. The fine roots were excavated and washed gently. Then, three fractions were taken from each sample. While the morphological analysis of mycorrhiza was carried out on fresh material on subsequent days, a fraction of the samples was frozen at −18°C (Bainard et al., 2010) for later analysis. The morphological analysis has to be done with fresh material because the color and swelling get lost after fixation in alcohol.

### 2.4 Morphological preparation of root samples

Root tips were examined using a dissecting microscope (Stemi DV 4; Zeiss, Jena, Germany). From each tree individual, three 5 cm root pieces of the first order were assessed as colonized with ectomycorrhiza, as indicated by a lighter color and swollen tips, or as not colonized. For further analysis, the frequency of active ectomycorrhizal root tips (ECT) was calculated using formula (1):

(1) $$ECT=\frac{Number\;of\;root\;tips\;with\;ectomycorrhiza}{Number\;of\;total\;root\;tips\;examined}*100\left[\%\right]$$

As we had to process 120 root samples, we were unable to prepare thin cross sections for all roots. Thus, the unequivoval identification of a Hartig net as another key characteristic of EM trees (Brundrett & Tedersoo, 2020) was not possible in all cases.

For assessing the mycorrhization with AM fungi, the samples were stained with Trypan blue following the protocol by Chabaud et al. (2006). An additional bleaching step was added against dark tannins in the tree roots, exposing the samples to hydrogen peroxide solution (10% H_2_O_2_) containing a few drops of ammonia (25% NH_3_) for 1.5 – 2 hr. The colonization of the root with AM was determined using a light microscope (Axiostar Plus; Zeiss, Jena, Germany). Examination of the arbuscular mycorrhization was estimated following Trouvelot et al. (1986), assessing 30 root fragments and using the software Mycocalc (https://www2.dijon.inrae.fr/mychintec/Mycocalc‐prg/download.html). We assessed AM mycorrhiza fungi by the presence of vesicles or arbuscules. While vesicles are not a common characteristic of Glomeromycota (Smith & Read 2009), their presence was a reliable characteristic of the tree species in our study. Vesicles contain abundant lipid and numerous nuclei, making them important storage structures (Smith & Read 2009). Across all roots sampled, we calculated the frequency of arbuscular mycorrhiza (AM F), the intensity of the arbuscular mycorrhizal colonization (AM M), and the relative abundance of arbuscules (AM A) as follows:

(2) $$AM\;F=\frac{Number\;of\;fragments\;with\;AMF}{Total\;number\;of\;root\;fragments\;}100\left[\%\right]$$

(3) $$AM\;M=\frac{\sum_{i=1}^{Total\;number\;of\;root\;fragments}{Proportion\;colonized\;by\;AM}_{i}\;}{Total\;number\;of\;root\;fragments}100\left[\%\right]$$

(4) $$AM\;A=\frac{\sum_{i=1}^{Total\;number\;of\;root\;fragments}{Proportion\;with\;arbuscules}_{i}\;*{\;Proportion\;colonized\;by\;AM}_{i}\;}{Total\;number\;of\;root\;fragments}100\left[\%\right]$$

### 2.5 DNA Extraction, Amplicon Library Preparation and Illumina`s Next Gen Sequencing

DNA was extracted from frozen root samples with the Qiagen DNeasy Plant Mini Kit (250, Cat No./ID: 69106C) according to the manufacturer's instructions. In short, the roots were ground manually with mortar and pestle in liquid nitrogen. An aliquot of 60 mg (*Betula, Carpinus, Fagus, Fraxinus, Prunus, Quercus, Tilia*) or 80 mg (*Acer, Aesculus, Sorbus*) of root tissue was used for DNA extraction. The DNA yield of every sample was checked with a NanoDrop ND‐8000 spectrophotometer (Thermo Fisher Scientific, Dreieich, Germany). In the following PCR, we targeted ITS4 and ITS7, using a primer mix containing P5‐5 N‐ITS4 (TCGTCGGCAGCGTCAGATGTGTATAAGAGACAG NNNNNTCCTCCGCTTATTGATATGC), P5‐6 N‐ITS4 together with P7‐3 N‐fITS7 (GTCTCGT GGGCTCGGAGATGTGTATAAGAGACAGNNNGTGARTCATCGAATCTTTG) and P7‐4 N‐fITS7 (GTCTCGTGGGCTCGGAGATGTGTATAAGAGACAGNNNNGTGARTCATCGAATCTTTG) (Gardes & Bruns, 1993; Ihrmark et al., 2012; Leonhardt et al., 2019). All PCRs were conducted using the proofreading HiFi HotStart ReadyMix DNA Polymerase (Kapa Biosystems, Boston, MA, United States), using the following thermal profile: initial denaturation at 95°C for 5 min, followed by 30–32 cycles of denaturation at 98°C for 20 s, annealing at 56°C for 20 s, elongation at 72°C for 20 s, followed by a final extension at 72°C for 5 min. The cleaning‐up steps were performed with the Agencourt AMPure XP kit (Beckman Coulter, High Wycombe, United Kingdom).

An index PCR was conducted to attach dual indices (barcodes) and adapters for the Illumina sequencing to the cleaned PCR amplicons, using the Nextera XT Index Kit (Illumina Inc., San Diego, CA, United States). DNA was quantified with PicoGreen using the Quant‐iT PicoGreen dsDNA Assay Kit (Molecular Probes, Eugene, OR, United States) and then pooled to provide equimolar representation of all samples. Fragment sizes and quality of DNA sequencing libraries were determined using the Aglient 2,100 Bioanalyzer (Agilent Technologies, Palo Alto, CA, United States). In preparation of sequencing, the pool was denatured for paired‐end sequencing of 2 × 300 bp with MiSeq v3 reagent Kit (Illumina Inc., San Diego, CA, United States). All molecular work was carried out at the Department of Soil Ecology of the Helmholtz Centre for Environmental Research—UFZ in Halle (Saale), Germany.

The sequencing data generated for this study were submitted to the European Nucleotide Archive (ENA) under the accession number PRJEB38894.

### 2.6 Bioinformatics workflow

The raw reads were de‐multiplexed by the Illumina MiSeq Reporter software package v2.5.1.3 with default settings. Fastq files were analyzed using the pipeline DeltaMP (v0.2)2 by following the workflow presented in Schöps et al., (2018). In brief, after trimming off primer‐ and index sequences, the reads received from the Illumina sequencing were quality filtered with MOTHUR (Schloss et al., 2009), resulting in a number of 12,446,975 reads. Short reads smaller than 300 bp and rare sequences were removed from the dataset, leaving 6,350,228 available reads after all quality checks. The reads were merged employing the PandaSeq algorithm with a threshold of 0.6 and a minimum overlap of 20 nucleotides and assigned to OTUs, using a 97% similarity threshold using cd‐hit‐est (Blaxter et al., 2005; Fu et al., 2012). Potential chimers were removed using UCHIME (Edgar et al., 2011). For the taxonomical assignment, the representative sequence was assigned according to the UNITE v7 reference database using the Bayesian classifier (Nilsson et al., 2019). In total, 6,107,557 reads were assigned to 15,366 OTUs, with 6,091,521 reads being clustered in the 3,018 most abundant OTUs. Afterward, 242,671 reads were removed from the dataset because they did not have a fungal origin. As a last step, 1,996 OTUs were categorized into fungal guilds with the Online Annotation Tool FUNGuild (Nguyen et al., 2016). For the other OTUs, the FUNGuild database did not provide additional information.

### 2.7 Statistical analyses

All statistical analyses and plotting of figures were carried out with R, Version 3.6.1 (R Core Team, 2019). Graphs were created using the ggplot2‐package (Wickham, 2016). The impact of host species identity, mycorrhiza host type (Myc_Type), and the plot's mycorrhiza mixture type (Mix_Type) were analyzed with linear mixed effects models, using the “lmerTest” package (Kuznetsova et al., 2017). Response variables were mycorrhization rates assessed by morphological methods (ECT, AM F, AM M, AM A) and OTU richness and abundance‐based coverage estimator (ACE, calculated using vegan, Oksanen et al., 2019), as alpha‐diversity indices, both of all fungi and separately by phylum (Ascomycota, Basidiomycota, and Glomeromycota). To test for species‐specific differences in mycorrhization rates, the model included plot as random factor. The model assumptions were checked visually by plotting QQ plots of quantiles of model residuals versus the expected quantiles of a normal distribution. As most percentage values did not meet the requirement of normal distribution of residuals, they were turned into proportions and sine square root transformed. To test for differences we applied type III analysis of variance with a subsequent and Tukey post hoc test (“ghlt”‐function in the multcomp package, Hothorn et al., 2008). As the morphological analysis of the roots of *Aesculus hippocastanum* did not allow to estimate ectomycorrhizal mycorrhization rates, we repeated this analysis without this host species.

To test for common responses across all ten tree species in the experiment, we constructed mixed models using the “lmerTest” package and applying a type III ANOVA (Kuznetsova et al., 2017). Predictors were mycorrhiza host type (Myc_Type) and the plot's mycorrhiza mixture type (Mix_Type), also including the interaction (Myc_Type x Mix_Type). Tree species identity and plot were crossed random factors. We built the same model for tree species richness instead of Mix_Type, using the log_2_ of plot trees pecies richness as numerical predictor, together with Myc_Type and the Myc_Type x species richness interaction. To derive estimates and standard errors for all factor combinations, we used the ggeffects package (Lüdecke, 2018).

Fungal community composition was analyzed with a redundancy analysis (RDA), using the rda function in the vegan package (Oksanen et al., 2019). To achieve normal distribution of the OTU count data, the read numbers were first square root transformed and then standardized by subtracting the mean value and dividing by standard deviation. RDA allowed us to assess the amount of variation explained by mycorrhiza host type (Myc_Type), the plot's mycorrhiza mixture type (Mix_type), and log_2_ of plot tree richness. The marginal effect of each of these three constraining variables was assessed with a permutational ANOVA (*n* = 999 permutations).

To assess which OTUs were enriched in certain host species (target species versus. all other species), mycorrhiza host type (AM versus. EM trees) or mycorrhiza host type (AM or EM tree versus. AM and EM trees growing together) was analyzed with a differential abundance analysis of the fungal OTUs with the DESeq2 packages (Love et al., 2014). Using negative binomial generalized linear models, log_2_ fold changes were calculated, based on data‐driven prior distributions. A Wald test was applied to test whether the estimated standard error of a log_2_ fold change was equal to zero. For these analyses, nonstandardized OTU counts were used, as implemented in the DESeq2 package (Oberholster et al., 2018). The significant results were visualized in scatter plots showing the log_2_ fold change as a function of log_10_ mean of normalized counts.

The molecular and morphological data on mycorrhization rates were correlated with each other, using the “rcorr”‐function of the Hmisc package (Harrell, 2020) and Spearman's rank correlation coefficient. The results of the correlation analyses were displayed with the corrplot package (Wei & Simko, 2017). To visualize the relationship of frequency (AM F) and intensity (AM M) of arbuscular mycorrhiza in the root system with OTU richness and ACE, we applied generalized linear mixed effects models, using a binomial error function and a logit link. We checked for overdispersion by dividing the residual deviance by the residual degrees of freedom. To scale AM F or AM *M* (that had been assessed on a percentage scale), we divided by 100. The model included tree species identity as random intercept and the interaction of either OTU richness or ACE with species identity as random slope.

## 3 RESULTS

### 3.1 Host tree mycorrhiza types

All tree species except *Aesculus hippocastanum* showed dual association with both mycorrhiza types (Table 1). While *Aesculus hippocastanum* showed arbuscular mycorrhiza in the root parenchyma (Table 1) and also swollen root tips covered with hyphae, we did not detect a typical hyphal mantle as in ectomycorrhizal associations or a Hartig net (Appendix Fig. S1). In all other species, we encountered ectomycorrhizal root tips (Appendix Figs. S2 and S3). In our morphological assessment, across all species, we detected dual mycorrhization in 79.1% of all analyzed root samples. There were significant differences between the tree species in the frequency of arbuscular mycorrhiza (AM F), the intensity of arbuscular mycorrhizal colonization in the root system (AM M), and the abundance of arbuscules in the root system (AM A, Table 1). In contrast, there were no differences between species in the frequency of active ectomycorrhizal root tips (ECT). Disregarding *Aesculus hippocastanum* in the assessment of ecto‐mycorrhization rates (ECT) did also not give significant differences between species. AM frequency ranged between 17% and 96%, with the lowest frequency in *Quercus petraea* and the highest frequency in *Fraxinus excelsior*, respectively. The same species showed the lowest (0.4%) and highest (22.4%) intensity of arbuscular mycorrhizal colonization (AM M), respectively. The species also differed in OTU richness, which ranged between 240 in *Betula pendula* and 490 in *Tilia platyphyllos* (Table 2).

**TABLE 2:** OTU richness per sample of all fungi and separately by phylum (Ascomycota, Basidiomycota, and Glomeromycota)

Abbreviation	Species	Total OTU richness		OTU richness Ascomycota		OTU richness Basidiomycota			OTU richness Glomeromycota	
Ac	*Acer pseudoplatanus* L.	409	ab	187	ac	61	b	b	107	b
Ae	*Aesculus hippocastanum* L.	316	ac	153	ab	45	b	bc	88	bc
Fr	*Fraxinus excelsior*L.	446	ab	193	ab	57	b	a	155	a
Pr	*Prunus avium* L.	415	a	152	bc	79	b	ab	107	b
So	*Sorbus aucuparia* L.	363	ac	194	ab	73	b	bd	61	bd
Be	*Betula pendula* Roth.	240	c	135	bc	76	b	d	9	d
Ca	*Carpinus betulus*L.	299	bc	172	ab	75	b	d	17	d
Fa	*Fagus sylvatica L*.	250	c	137	b	45	b	d	20	d
Qu	*Quercus petraea*(Matt.) Liebl.	481	a	143	bc	293	a	cd	24	cd
Ti	*Tilia platyphyllos* Scop.	490	a	226	a	53	b	d	42	cd

Note

Small letters show significant differences between species according to a Tukey post hoc test.

**FIGURE 1:**
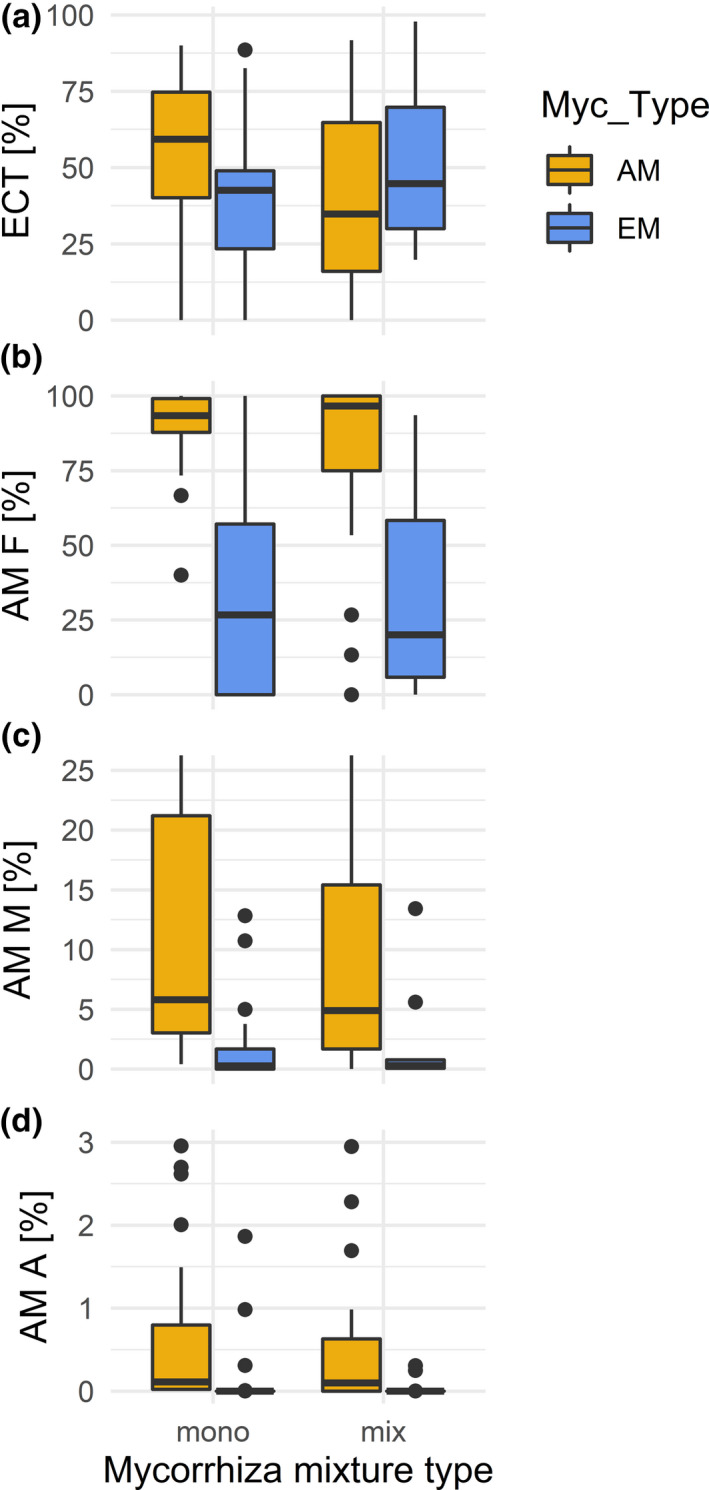
Mycorrhization rates of tree species with a preference for either AM or EM (Myc_Type) when grown in monotypic compositions (mono, i.e., either AM or EM) or mixed types (mix, i.e., trees with a preference for AM or EM growing together). a) Frequency of active ectomycorrhizal root tips (ECT), b) Frequency of arbuscular mycorrhiza in the root system (AM F), c) Intensity of arbuscular mycorrhizal colonization in the root system (AM M), and d) Abundance of arbuscules in the root system (AM A). For statistically significant differences, see Table 3

**FIGURE 2:**
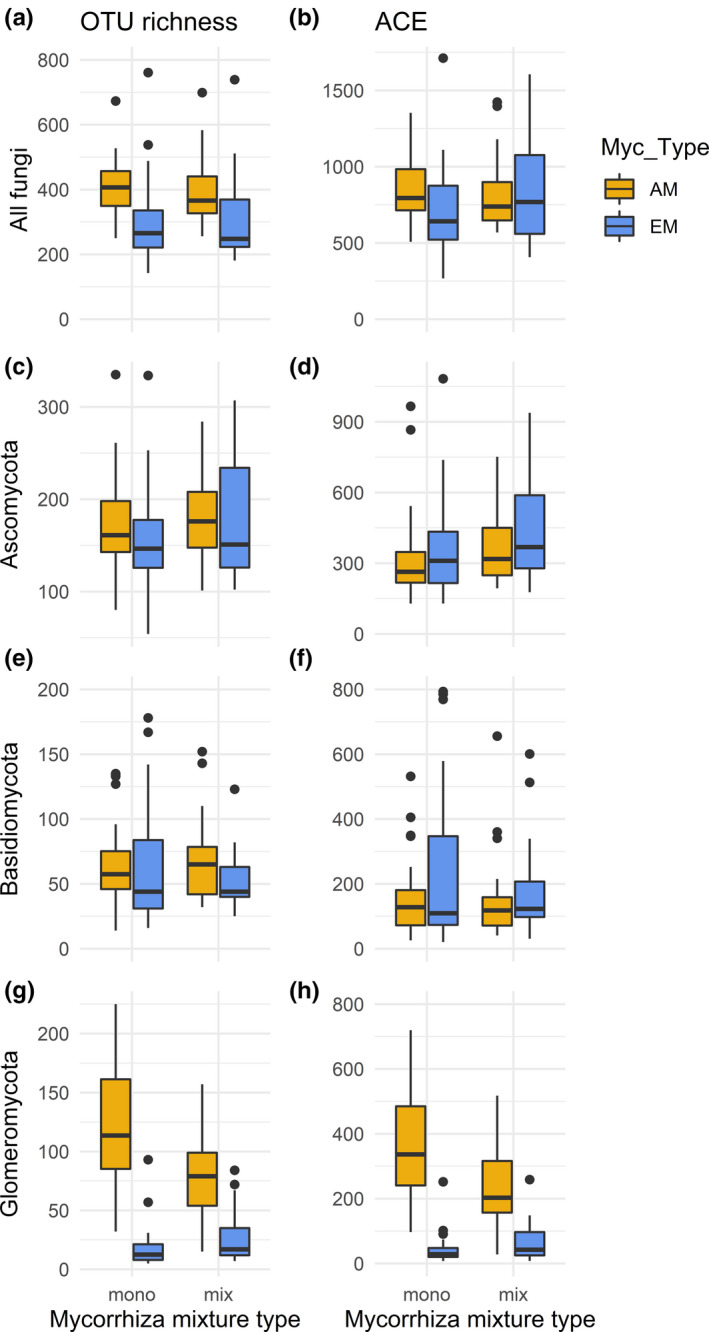
OTU richness (left column) and abundance‐based coverage estimator (ACE, right column) per sample of tree species with a preference for either AM or EM (Myc_Type) when grown in monotypic (mono, i.e., either AM or EM tree species) or mixed‐type compositions (mix, i.e., tree species with a preference for AM or EM growing together). a) and b) All fungal taxa, c) and d) Ascomycota, e) and f) Basidiomycota, g) and h) Glomeromycota. For statistically significant differences, see Table 3

The morphological and molecular measures of mycorrhization rates also differed between AM and EM host tree species, that is between species that were known to prefer either AM or EM as fungal partners (Table 3, Figures 1 and 2). Compared to EM host tree species, AM species had a significantly higher AM frequency (AM F, Figure 1b) and intensity of arbuscular mycorrhizal colonization (AM M, Figure 1c). In particular, AM tree species had a higher OTU richness and abundance‐based coverage estimator (ACE) of Glomeromycota (Table 3, Figure 2g and h). In contrast, the two mycorrhizal types did not significantly differ in frequency of active ectomycorrhizal root tips (ECT, Figure 1a) and abundance of arbuscules (Figure 1d). Similarly, there were no differences in OTU richness and ACE of all fungi (Figure 2a, b), Ascomycota (Figure 2c, d), and Basidiomycota (Figure 2e, f, Table 3).

**TABLE 3:** Linear mixed effects models relating mycorrhization rates assessed by morphological methods as well as OTU richness and ACE to the plot's mycorrhiza mixture type (Mix_Type) and mycorrhiza host type (Myc_Type). Shown are estimates (est) and standard errors (se) of marginal effects of fixed factors as well as *p* values based on a type III ANOVA, with significant *p* values given in bold fonts

	Mono_AM		Mono_EM		Mix_AM		Mix_EM		Mix_Type	Myc_Type	Mix_Type: Myc_Type
	est	se	est	se	est	se	est	se	*p* value	*p* value	*p* value
ECT	54.38	4.72	39.58	4.58	40.80	5.47	50.52	5.56	0.8028	0.6244	0.0137
AM F	89.73	4.89	32.70	4.51	81.16	5.95	32.37	6.38	0.4183	**<0.0001**	0.4538
AM M	12.22	1.95	3.46	1.82	9.88	2.30	3.07	2.43	0.4968	**0.031**	0.6269
AM A	0.88	0.41	0.45	0.39	0.40	0.46	0.48	0.49	0.5595	0.7343	0.5091
OTU richness all fungi	397.08	42.90	329.27	42.65	381.04	45.13	375.40	45.28	0.4935	0.5470	0.1586
ACE all fungi	855.04	89.00	783.00	88.25	811.63	95.28	929.17	95.80	0.3353	0.8533	0.0770
OTU richness Ascomycota	169.74	14.76	152.90	14.65	182.52	15.70	177.44	15.78	0.0649	0.5844	0.5246
ACE Ascomycota	339.55	61.90	365.28	61.43	387.64	65.46	509.78	65.75	0.0189	0.3941	0.1892
OTU richness Basidiomycota	62.81	32.51	100.16	32.39	64.56	33.68	113.58	33.75	0.5921	0.3642	0.6767
ACE Basidiomycota	149.73	72.10	292.65	71.70	139.12	73.84	262.16	74.00	0.6434	0.1948	0.8000
OTU richness Glomeromycota	121.31	12.09	18.00	12.04	77.33	12.44	28.86	12.46	**0.0102**	**0.0021**	**<0.0001**
ACE Glomeromycota	356.65	28.89	42.17	28.93	235.03	32.52	73.20	34.80	0.0605	**0.0006**	**0.0018**

Note

Mix_Type: the plot's mycorrhiza mixture type (Mono: AM or EM; Mix: AM and EM trees growing together). Myc_Type: the tree species’ preferred type of mycorrhiza (AM, arbuscular mycorrhiza; EM ectomycorrhiza). ECT: Frequency of active ectomycorrhizal root tips. AM F: Frequency of arbuscular mycorrhiza in the root system. AM M: Intensity of the arbuscular mycorrhizal colonization in the root system. AM A: Arbuscular abundance in the root system. OTU richness and ACE (abundance‐based coverage estimator) of all fungi and separately by phylum (Ascomycota, Basidiomycota, and Glomeromycota).

**FIGURE 3:**
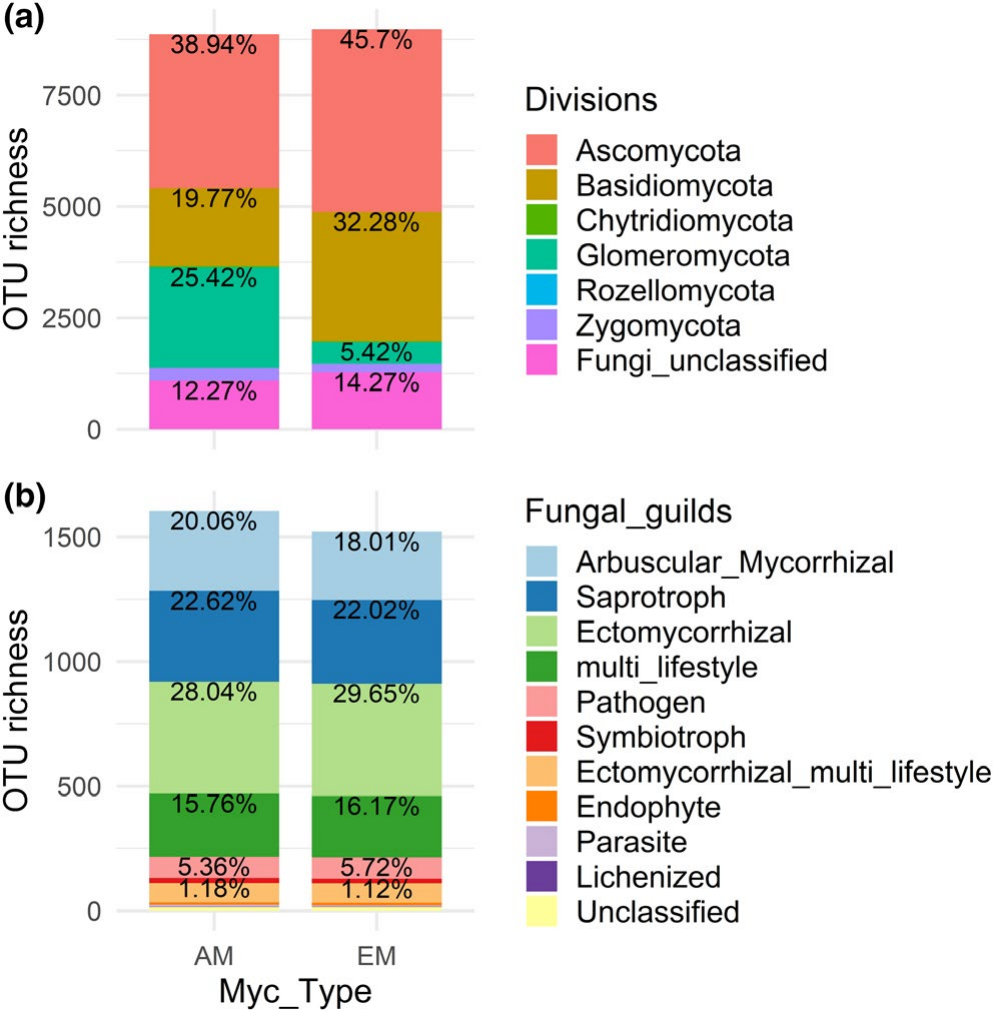
OTU richness across the whole study shown a) by fungal divisions and b) by fungal guild, separately by host tree species with a preference for either AM or EM (Myc_Type). a) is based on the total number of 15,366 OTUs detected across all samples, while b) refers to the 1,996 OTUs listed in FUNGuild (Nguyen et al., 2016). a) OTU richness by taxonomic divisions with percentages for Chytridiomycota 0.34% and 0.21%, Rozellomycota 0.011% and < 0.001%, Zygomycota 3.26% and 2.12%, respectively. b) OTU richness by fungal guilds with percentages for Ectomycorrhizal multi‐lifestyle 4.86% and 5.19%, Endophyte 0.62% and 0.72%, Parasite 0.498% and 0.329%, Lichenized 0.062% and 0.066%, and Unclassified 0.953% and 0.986%, respectively. All guilds containing more than one lifestyle were pooled in the group of “multi‐lifestyle.”

AM and EM tree species did not differ in OTU richness across all samples (Figure 3a). In both mycorrhiza host types, Ascomycota contributed most to read number and OTU richness, followed by Basidiomycota in EM tree species and Glomeromycota in AM tree species. However, Basidiomycota were also present in AM trees (19.8% of all OTUs), compared to 32.3% in EM trees. In contrast, Glomeromycota were less abundant in EM trees (5.4% of all OTUs), compared to 25.4% in AM trees. The other fungal divisions (Chytridiomycota, Rozellomycota, and Zygomycota) did not differ much in total OTU richness between the two host types. There were not many differences in OTU richness with respect to fungal guild assignment (Figure 3b). Total OTU richness of arbuscular fungi was comparable in AM and EM trees species (with 20.1% and 18.0%, respectively), as was total OTU richness of ectomyorrhizal fungi (with 28.0% and 30.0%, respectively) and of multi‐lifestyle fungi (15.8% and 16.2%, respectively). When OTU richness was compared at the species level, Basidiomycota contributed between 13.2% and 63.6% of all OTUs in *Tilia platyphyllos* and *Quercus petraea*, respectively (Appendix Fig. S4a). In contrast, the proportion of Glomeromycota ranged between 3.3% and 40.1% in *Betula pendula* and *Fraxinus excelsior*, respectively. However, the differences among tree species were less pronounced in the fungal guild assignment (Appendix Fig. S4b). At the level of individual samples, there was not a single root sample that did not have at least five different OTUs of both arbuscular and ectomyorrhizal fungi, revealing a 100% dual mycorrhization across all samples.

**FIGURE 4:**
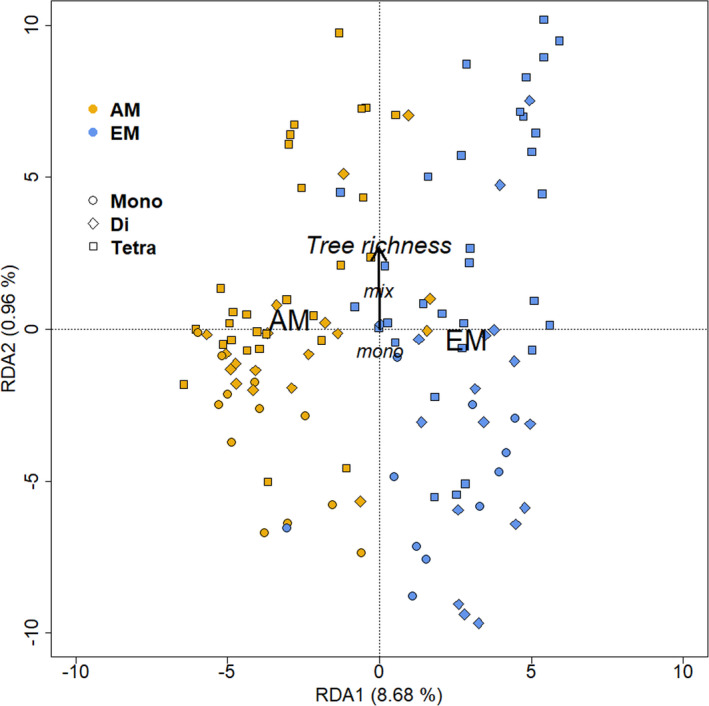
Redundancy analysis (RDA) of the fungal community composition based on the total number of 15,366 OTUs. Constraining variables were the preferred mycorrhiza type of the tree species (Myc_Type, which was either AM or EM), tree richness (log_2_ transformed with 0, 1, and 2 for tree richness levels 1, 2, and 4, respectively, shown as Mono, Di and Tetra species combinations in the legend) and mycorrhiza mixture type (Mix_Type), with monotypic (either AM or EM tree species) or mixed‐type compositions (AM or EM tree species growing together). While Myc_Type was a significantly constraining variable according to a permutation test (*p* =0.001), tree richness was only marginally significant (*p* =0.088) and Mix_Type was insignificant (*p* =0.4)

**FIGURE 5:**
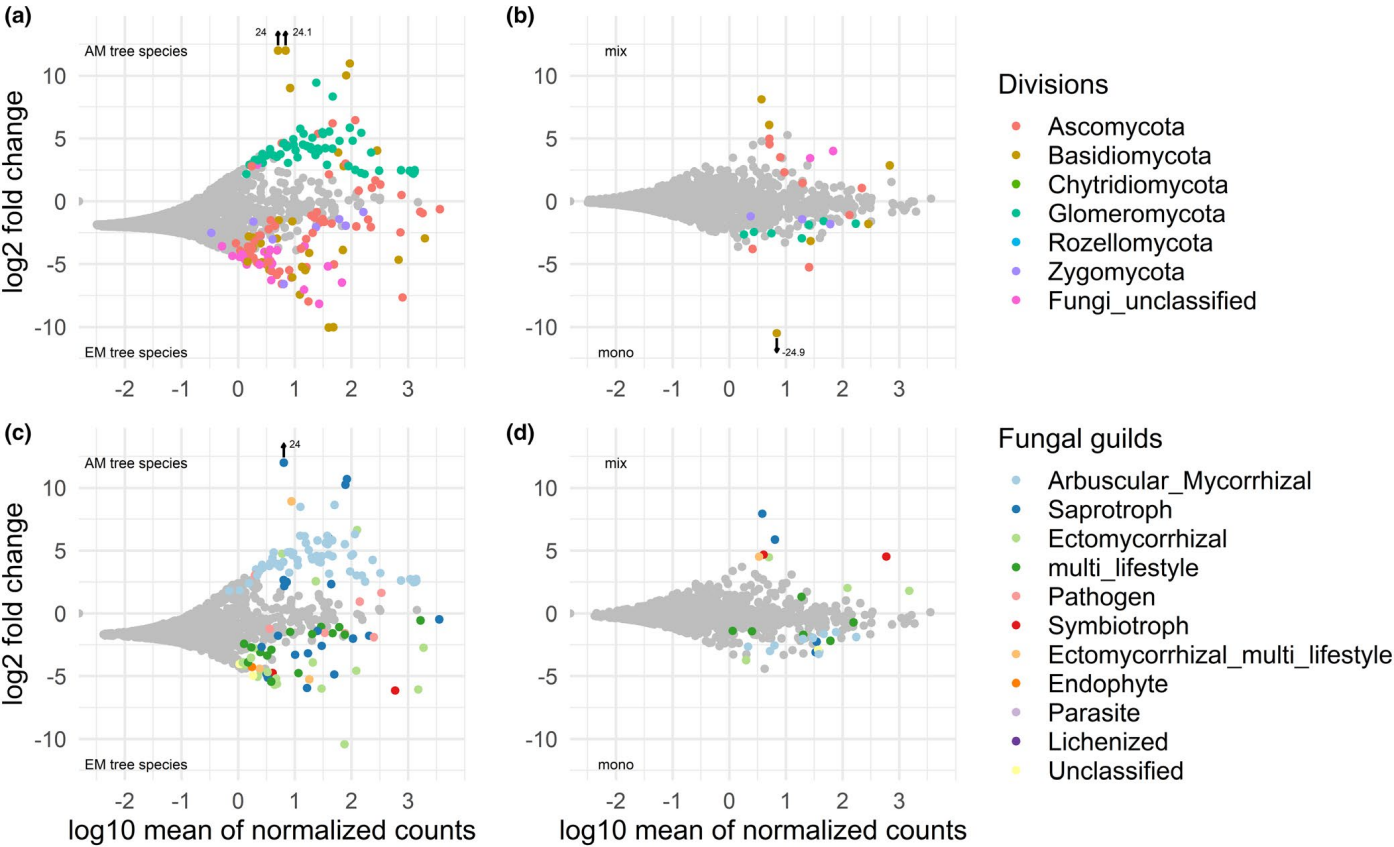
OTUs enriched in AM tree species (positive log_2_ fold changes)or in EM tree species (negative log_2_ fold changes) (a and c) and in mixed (AM or EM tree species growing together) as opposed to monotypic (only either AM or EM trees) (b and d) plotted against abundance (log_10_ mean of normalized counts). Insignificant fold changes are shown in gray, while significant fold change according to a Wald test (*p* <0.05) are highlighted in color. a) and b) significant fold changes by taxonomic division, based on all 15,366 OTUs detected across all samples. c) and d) significant fold changes by taxonomic fungal guilds, based on the 1,996 OTUs listed in FUNGuild (Nguyen et al., 2016)

AM and EM host tree species also differed significantly in fungal community composition, as host type was strongly correlated with the first RDA axis (Figure 4). The marginal effect of mycorrhizal host type (Myc_Type) was highly significant (*p* <0.001 in a permutational ANOVA) and explained 9.5% of the total variation in fungal community composition. Both mycorrhizal host types were characterized by particular OTUs that occurred significantly more often in either type (Figure 5a). According to a pairwise Wald test, there were 77 and 56 OTUs significantly enriched in AM and EM trees, respectively, using an unadjusted threshold of *p* =0.05 (Appendix Table S2). In the case of AM trees, these characteristic OTUs mostly belonged to the Glomeromycota, with highest degree of enrichment for *Glomus* sp., *Rhizophagus irregularis,* and *Septoglomus viscosum* (with log_2_ fold changes 9.4, 8.4, and 5.5, respectively). However, there were also some taxa of Basidiomycota encountered more frequently in AM trees, such as *Psathyrella corrugis* (log_2_ fold change 24.0), Agaricomycetes (24.1), and Stephanosporaceae (10.0) as well as of Ascomycota, such as *Pulvinula* sp. (6.5) and *Thielaviopsis basicola* (6.2). In contrast, OTUs found more often in EM trees belonged to all divisions other than Glomeromycota (Figure 5a). In decreasing degree of enrichment, the fungal taxa were assigned to Sebacinales (−10.1, Basidiomycota), *Tuber* sp. (−8.0, Ascomycota), *Tomentella ellisii* (−7.4, Basidiomycota), *Mortierella dichotoma* (−6.6, Zygomycota), *Geopora cervina* (−5.8, Ascomycota), and *Scleroderma areolatum* (−4.8, Basidiomycota). These differences were also reflected in the assignment of OTUs to fungal guilds (Figure 5c), where AM trees were positively characterized by OTUs assigned to arbuscular mycorrhiza, as well as by single OTUs of saprotrophic, ectomycorrhizal, and pathogenic lifestyle, while the OTUs significantly enriched in EM trees could belong to any guild other than arbuscular mycorrhiza (Figure 5c). Here, the three OTUs with highest enrichment in AM trees (*Psathyrella corrugis* and two taxa of Stephanosporaceae) were all saprotrophic fungi (Appendix Table S2).

Between two (*Aesculus hippocastanum, Sorbus aucuparia*) and 19 OTUs (*Fraxinus excelsior*) were significantly enriched in the roots of a particular tree species compared to all other tree species (Appendix Figs. S5 and S6, Appendix Table S3). While in most all AM tree species the significantly enriched OTUs belonged to both Glomeromycota and Basidiomycota or Ascomycota, *Aesculus hippocastanum* was exclusively associated with saprotrophic Ascomycota (Appendix Fig. S5c, d), which were *Acremonium implicatum* (also occurring as endophyte, Dongyi & Kelemu, 2004) and *Sarocladium* sp. Among OTUs highly concentrated in the roots of certain tree species were also pathogens, such as *Plectosphaerella alismatis* in *Fraxinus excelsior, Cyphellophora* sp. in *Prunus avium* and *Sorbus aucuparia, and Mycosphaerella tassiana* in *Betula pendula* (Appendix Table S3). However, most of the fungal taxa enriched in EM trees belonged to Basidiomycota (such as *Scleroderma areolatum* in *Quercus petraea*).

### 3.2 Tree species diversity

Host tree richness per plot did not have any significant effects on any of the morphological or molecular variables for mycorrhization rate (Appendix Table S4). There was only one significant interaction between mycorrhiza type of the host tree species and these variables, which was for ACE of Basidiomycota (Appendix Table S4). While in mono‐specific stands, ACE of Basidiomycota was lower in EM than AM tree species, ACE of Basidiomycota was similar in two‐species mixture and higher in EM than AM tree species in four‐species mixtures (data not shown). Consistent with the design of the study that made sure that all mycorrhizal types of host tree species occurred in all richness levels, tree richness was orthogonal to the first RDA axis that described host type (Figure 4). The effect of tree richness on overall fungal community composition was only marginally significant (*p* =0.088 in a permutational ANOVA) and explained 1.1% of the total variation.

### 3.3 Mixing host tree mycorrhiza types

Similar to host tree richness, the plots’ mycorrhiza mixture type (Mix_Type), with monotypic (either AM or EM tree species) or mixed‐type compositions (AM and EM tree species growing together), had also no significant effect on the morphological assessment of mycorrhization (Table 3, Figure 1). However, mixture type had a significant effect on OTU richness of Glomeromycota and a marginally significant effect on ACE of Glomeromycota. For both variables, there was also a significant interaction of the host's mycorrhiza type and mixture type (Table 3). While mixing of AM and EM tree species reduced both OTU richness and ACE of Glomeromycota in AM tree species, it increased OTU richness and ACE of Glomeromycota in EM tree species (Figure 2g and h). Although mycorrhiza mixture type explained only an insignificant proportion of fungal community composition (0.9%, *p* =.4), there were 11 OTUs that were significantly enriched in tree communities that consisted both of AM and EM trees (Figure 5b). These OTUs belonged to both Basidiomycota and Ascomycota, but did not comprise any Glomeromycota. These species were mostly ectomycorrhizal (*Hebeloma* sp., *Tuber maculatum*), but comprised also saprotrophs (such as *Flagelloscypha minutissima, Psathyrella corrugis, Strumella* sp.) and multi‐lifestyle fungi (*Nectria ramulariae*) (Figure 5d).

### 3.4 Correlation between the morphological and molecular assessment

**FIGURE 6:**
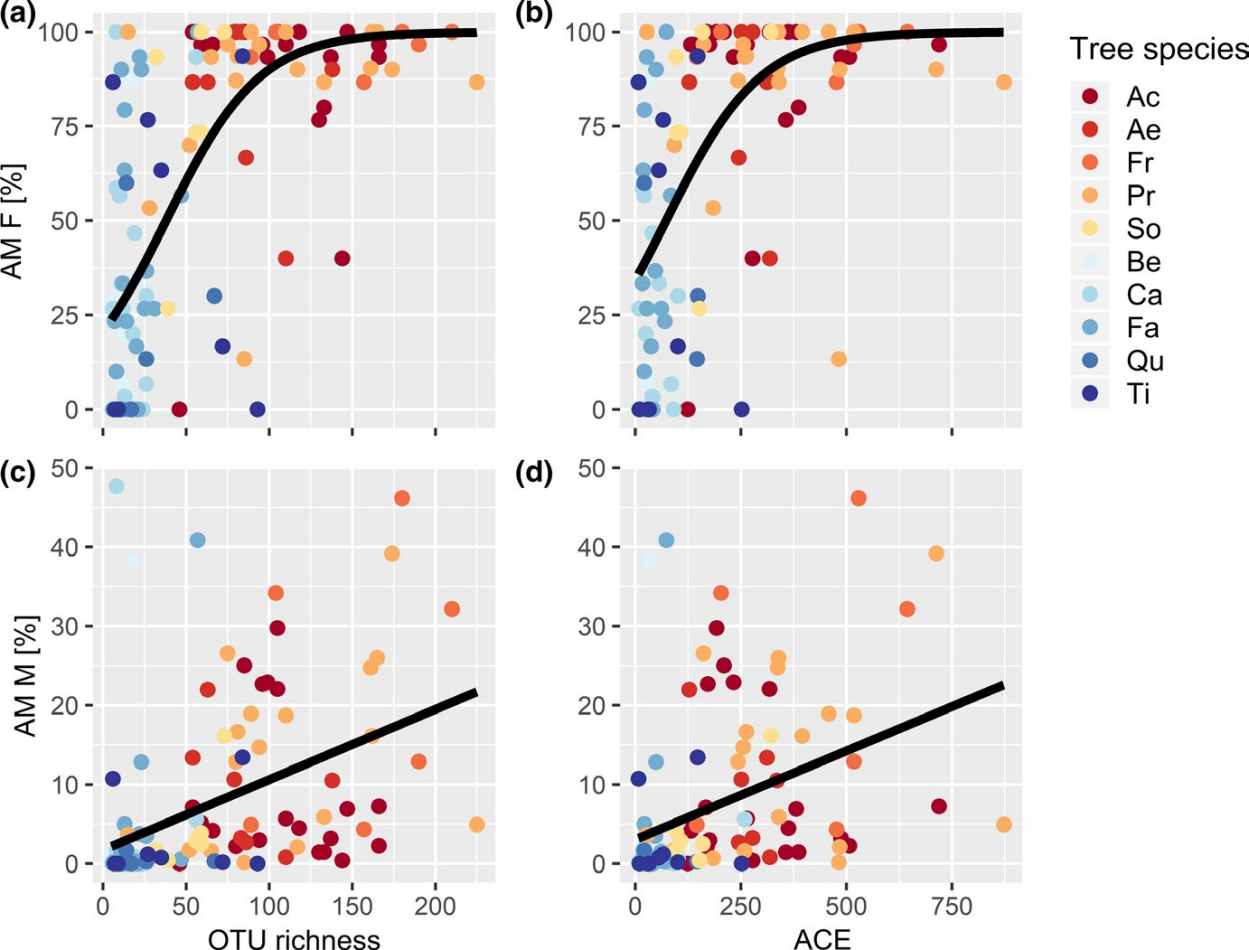
Relationship between mycorrhization rates assessed by microscopy and molecular data for Glomeromycota as obtained from next‐generation sequencing. a) and b) Frequency of arbuscular mycorrhiza in the root system (AM F) as a function of a) OTU richness of Glomeromycota and b) abundance‐based coverage estimator (ACE). The regression lines were obtained from generalized linear mixed effects models, using a binomial error function and a logit link. Logit estimates were 0.03543 and 0.00893 for a and b, respectively. c) and d) Intensity of arbuscular mycorrhizal colonization in the root system (AM M) as a function of c) OTU richness of Glomeromycota and d) ACE of Glomeromycota. The regression lines were obtained from linear mixed effects models, with estimates of 0.000876 and 0.000224 for a and b, respectively. All regressions have *p* <0.0001

While the frequency of active ectomycorrhizal root tips (ECT) showed no correlation with any phylum in OTU richness or ACE in the molecular assessment (Appendix Fig. S7), the frequency of arbuscular mycorrhiza in the root system (AM F) was strongly correlated with OTU richness and ACE of Glomeromycota (Figure 6a, b). However, this correlation was not linear because the AM F mycorrhization rate attained 100% in many samples. A similarly positive, but linear relationship was encountered for the intensity of arbuscular mycorrhizal colonization in the root system (AM M, Figure 6c, d). In addition, AM F was also strongly positively correlated with richness of Chytridiomycota and Zygomycota and weakly negatively with ACE of Ascomycota (Appendix Fig. S7). There was no correlation of the abundance of arbuscules in the root system (AM A) with any molecular variable of mycorrhization rate (Appendix Fig. S7).

## 4 DISCUSSION

### 4.1 Dual mycorrhization

A main outcome of our study was that almost all tree species showed dual mycorrhization, which was revealed both by the morphological and molecular assessment. However, AM and EM tree species clearly preferred certain fungal taxa and differed in fungal community composition. Although, in total, Ascomycota clearly dominated root fungal composition both with respect to OTU richness and abundance‐based coverage estimator (ACE), we encountered both Glomeromycota and Basidiomycota OTUs in comparable amounts, being more concentrated in AM and EM tree species, respectively. These findings fully confirm our first hypothesis of clear preferences of mycorrhiza types between AM and EM host tree species. Thus, our results add to the previous evidence of dual‐mycorrhizal plant species that have been reviewed by Teste et al., (2020). However, we have to consider that a dual mycorrhization is a dynamic process. Studying the mycorrhization of *Populus trichocarpa* during soil succession of a floodplain, Piotrowski et al., (2008) found a shift in abundance from AM to EM. With changing soil characteristics such as increasing content of soil organic matter, organic soil phosphates and potassium and decreasing nitrate, the abundance of EM increased. Confirming the findings of Piotrowski et al., (2008), Albornoz et al., (2016) described that AM fungi prefer younger soils in which phosphates are mainly available in inorganic forms. Thus, the increase of EM fungi in later successional stages might be linked to their ability to access organic phosphate fractions in the soils (Antibus et al., 1992).

We have to point out that our results are only valid for the particular conditions of our experiment, with young trees growing on former arable soil. We assessed mycorrhization only 2.5 years after the experiment had been established. Under such conditions, we would have expected that the fungal community would be dominated by species that were already present in the former farmland. The high proportion of Ascomycota conforms to findings from studies on arable soils (Bainard et al., 2015; Klaubauf et al., 2010). The Haplic Chernozem at our experimental site is a particular example of an exceptionally fertile farmland soil. In 1902 and near to our site, a “Static Fertilization Experiment” had been established with the aim to provide a comprehensive understanding of the effects of long‐term fertilization on the yields and quality of crops (Merbach & Schulz, 2013). In this experiment, Francioli et al., (2016) analyzed the fungal soil communities and found a predominance of Ascomycota (76.4% of reads; 431 OTUs), followed by Basidiomycota (12.6% of reads; 111 OTUs), Zygomycota (10.4% of reads; 30 OTUs), Chytridimycota (0.3% of reads; 14 OTUs), and Glomeromycota (0.02% of reads; 2 OTUs). Similar results were reported by Moll et al., (2016) who analyzed the spatial distribution of fungal communities in agricultural nutrient‐rich soils (Luvisol) in Lower Saxony (Central Germany). The most abundant fungal division was the Ascomycota with 68.3% of sequences, followed by Basidiomycota (2.4%) and Glomeromycota (less than 1%). Ascomycota are the dominant fungal phylum in agro‐ecosystems, since they are mainly important decomposers of organic matter, in particular of leaf litter (Lienhard et al., 2014). In addition, many of the Basidiomycota encountered by us were saprotrophs, too. In agricultural soils in Michigan (USA), most of the taxa belonged to the Agaricales, which were considered saprotrophs in litter and soil (Lynch & Thorn, 2006). However, in our case Agaricales were not only represented by saprotrophs, such as *Psathyrella corrugis*, *Flagelloscypha minutissima,* and *Calyptella* sp. but also by EM such as *Hebeloma* sp. Brundrett (2002) and Tedersoo et al., (2014) emphasized that ectomycorrhizal fungi would not be expected in agro‐ecosystems that lack suitable host plants. Indeed, EM communities at our study site were relatively species‐poor compared to those growing in their natural environment (Buée et al., 2009; Francioli et al., 2016). Many ectomycorrhizal fungi such as *Geopora cervina, Scleroderma areolatum,* and *Hebeloma* sp. are typical of nutrient‐rich soils. Komonen et al., (2016) studied macro‐fungi on such former farmland soils that had been afforested with *Betula pendula, Picea abies,* or *Pinus sylvatica* in Southern Finland 20 years before. The authors pointed out that afforested farmland can be a macro‐fungal hotspot for fungal species with a preference for nutrient‐rich soils. In the transition from farmland to forest after abandonment and afforestation, the change in EM community composition can take up to 80–100 years or more (Kałucka & Jagodziński, 2016).

*Scleroderma areolatum* is a frequent ectomycorrhizal fungus with a wide range of host species (Jeffries, 1999), and species of this genus have been reported from extreme habitats such as mine heaps and ore‐roasting beds (Jones & Hutchinson, 1986; Marescotti et al., 2013). *Scleroderma areolatum* seems to be a particularly effective early colonizer, as it is used for seedling inoculation in tree nurseries of *Fagus sylvatica* (Mrak et al., 2017). Thus, we cannot exclude that species such as *Scleroderma areolatum* had been introduced from the seedling nursery when the trees had been planted. The whole genus *Scleroderma* as EM fungal genus has a low host specificity (Mrak et al., 2017). For example, fruit bodies of *Scleroderma citrinum* were found in *Quercus rubra* plantations of different age (5, 21, 33, 43 years) on disturbed soils after lignite mining (Gebhardt et al., 2007). Here, the fungal colonization rate increased with the age of the plantation. Similarly, we would expect an increase in EM fungal taxa with ongoing age of the experiment.

We also have to consider that the occurrence of Glomeromycota in roots of EM trees might be an effect of this early colonization stage in the experiment. However, Glomeromycota have been described to be abundant on the same soil type as in our study (i.e., Chernozems, see Baltruschat et al., 2019). Brundrett (2002) suggested that AM in plants with dual associations might either be relicts, provide additional functions (such as increased access to nutrients) or be a backup mechanism. It might also be that only the AM fungus in an association with EM trees might benefit from this association (Teste et al., 2020). If the costs for the EM hosts are low, there would be no need to exclude AM colonization. Finally, as also pointed out by Teste et al., (2020), some fungal taxa might simply be promiscuous, equally well forming ectomycorrhizal and arbuscular mycorrhizal associations.

### 4.2 Tree species diversity and mycorrhization

In contrast to our second hypothesis, host tree species richness had no significant effects on mycorrhization, except for enhancing ACE of Basidiomycota in EM trees and decreasing it in AM trees. This result contradicts previous findings of generally positive host plant diversity effects on soil microbial diversity (Hiiesalu et al., 2014; Lange et al., 2015; Steinauer et al., 2016; Tedersoo et al., 2016; Weißbecker et al., 2019). There are several explanations for this finding. First, the high nutrient availability could have suppressed the development of a rich symbiotic soil biome. Soil type at the study site is Chernozem (Black Earth), which belongs to the most productive soils in the world (Allaby et al., 2012). This would explain why among the EM fungal taxa we encountered many generalists and only a few specialists. However, despite these nutrient‐rich conditions, we detected both AM and EM. Second, the experiment might not have been old enough to reveal such effects. It is well known that biodiversity effects develop with time (Eisenhauer et al., 2012; Guerrero‐Ramírez et al., 2017), and this is particularly true for plant–soil microbial associations (Eisenhauer et al., 2010). Thus, host richness effects might become more apparent in the future. Third, in addition to the young age of the experiment in terms of recent soil disturbance and short time for microbial community assembly, we also worked with 4‐ to 5‐year‐old trees. For example, OTU richness was observed to increase in nursery‐grown *Larix decidua* saplings (Leski et al., 2008). Furthermore, Beiler et al., (2010) described that the connectivity to the fungal network depended on the age of the tree. Thus, it might well be that the trees in our experiment have not yet acquired the full set of symbionts. Fourth, it was shown that initial biodiversity effects are often dominated by selection effects, while over time, biotic interactions increase, and complementarity effects become more important and net biodiversity more significant (e.g., Huang et al., 2018; Marquard et al., 2009; Reich et al., 2012).

### 4.3 Mixing host tree mycorrhiza types

Similar to tree species richness, mixing AM and EM tree species had no effect on most measures of mycorrhization. In particular, OTU richness and ACE of ectomycorrhizal fungi were not increased in AM trees when they grew together with EM trees. Such effects had been described by Bahram et al., (2011) for *Populus tremula* in Estonia and by Teste et al., (2014) for *Melaleuca preissiana* in southwestern Australia, but might not have occurred at our site because of the previous land use history. However, such mix‐type effects were encountered for Glomeromycota. Here, OTU richness and ACE of Glomeromycota in AM tree species were reduced when EM trees were present, which would be explained by the reduced density of typical AM host tree species. Thus, biomass and propagules of AM fungi would be lower than in pure AM tree plots. In consequence, OTU richness and ACE in the remaining AM host trees was reduced. In contrast, AM trees increased OTU richness and ACE of Glomeromycota in EM trees. Similar findings have been described by Dickie et al., (2001), when planting seedlings of *Quercus rubra* (EM tree species) next to *Acer rubrum* (AM tree species). This indicates spill‐over effects of arbuscular mycorrhizal fungi from AM to EM trees. To our knowledge, our study shows for the first time such mutual interaction effects of host tree species of contrasting mycorrhiza type. Thus, at least for Glomeromycota we can confirm the third hypothesis, while we have to reject it for the other taxa.

The underlying mechanisms, however, are still unclear. We cannot distinguish whether the reduction in Glomeromycota OTU richness in the presence of EM trees is a pure host density effect or an active suppression effect of more abundant ectomycorrhizal fungi in the soil. Separation of simple dilution effects from active suppression or competition would require mono‐specific stands with different tree densities, which were not included in the study. Conversely, such a potentially suppressive effect was not observed for AM tree species reducing the OTU richness and ACE of Basidiomycota in EM tree species, which is probably explained by the fact that a large part of Basidiomycota might also grow without a host, thus making this effect independent of host density.

Moreover, certain OTUs were significantly enriched in plots where AM and EM trees were mixed. In principle, these fungal taxa would be suitable candidates of connecting AM and EM trees. Such belowground networks between different host species have been described (Bahram et al., 2011; Weiss et al., 2004), but so far no particular taxa have been identified that would particularly link AM with EM trees. Although there were only 11 taxa that were enriched in AM‐EM mix‐type plots, some of them were symbiotic or had a multi‐lifestyle that included symbiotic interactions with plants. The finding that the symbiotic taxa in this group were exclusively members of Ascomycota and Basidiomycota, but not of Glomeromycota, suggests that fungi specialized in linking AM and EM trees are ectomycorrhizal and not arbuscular mycorrhizal. This finding could be explained by the obligate biotrophic character of AM fungi, while EM fungi can additionally acquire resources as saprophytes (e.g., Sebacinaceae, Selosse et al., 2002). Assuming that EM fungal networks extend over much larger areas than AM fungi, they would also encounter more different mycorrhizal host types. Potential candidates of such “link” species in our study are *Hebeloma* sp. (Basidiomycota) and *Tuber maculatum* (Ascomycota). A next step would be to test these links experimentally by inoculating trees with these taxa in comparison with fungi from other groups. The idea that “link” species are ectomycorrhizal fits to the observation that only approximately 10% of the EM fungal taxa are truly host‐specific (Rog et al., 2020; van der Linde et al., 2018). If associations with multiple host trees are widespread among EM fungi, these associations might also include AM hosts. However, an alternative explanation could also be that EM fungi connecting AM and EM trees are a very common characteristic and that many EM fungal taxa play this role.

### 4.4 Correlation between the morphological and molecular assessment

Comparing the morphological assessment with the molecular approach allowed us identifying the methods that are consistent or complementary in quantifying mycorrhization rates. Here, we can confirm the fourth hypothesis of a congruence between both approaches only for arbuscular mycorrhiza. There was a close relationship between the frequency and intensity of arbuscular mycorrhiza in the root system (AM F and AM M, respectively) and OTU richness and ACE of Glomeromycota. To our knowledge, no other study has reported a similar high congruence between morphological and molecular assessments, as most authors tended to emphasize the difference between both approaches (e.g., Bainard et al., 2015). In contrast, we did not encounter any significant relationship between the frequency of active ectomycorrhizal root tips and Basidiomycota or Ascomycota. Thus, our morphological results on the frequency of active ectomycorrhizal root tips (ECT) were not confirmed by the molecular data, which reflects the difficulties associated with the morphological determination of EM (Agerer, 2001; Kottke & Oberwinkler, 1986). In particular, saprotrophic hyphae may have been confounded with ectomycorrhizal root tips.

## 5 CONCLUSION

Our results clearly supported the emerging view that most tree species host both AM and EM (Teste et al., 2020). It is known that both mycorrhiza types contribute differently to the benefit of their host, with EM fungi mobilizing nutrients both from mineral and organic sources (Abuzinadah & Read, 1989) and AM fungi mainly from mineral sources (Hodge et al., 2001). To shed light on the potentially complementary effects of simultaneous association with EM and AM, resource tracer experiments would have to be conducted (Gockele et al., 2014). Tracers would also be one suitable approach to test whether the potential “link” species in the fungal network, which we identified only by their more frequent occurrence in plots with mixed mycorrhiza host types, actually connect different tree species.

Our study has greatly increased our knowledge on mycorrhizal interactions with host trees of different preferences to particular mycorrhiza types. Despite the young age of the experiment, these interactions were already well reflected in our morphological and molecular assessments. Thus, our findings are highly relevant for young tree plantations and reforestation sites, which are very widespread globally. Mixing AM and EM trees in such plantations might have strong positive ecological effects.

## CONFLICT OF INTEREST

The authors declare to have no conflict of interest.

## AUTHOR CONTRIBUTION

Heike Heklau: Conceptualization (equal); Formal analysis (supporting); Investigation (equal); Methodology (equal); Supervision (lead); Validation (supporting); Visualization (supporting); Writing‐original draft (supporting); Writing‐review & editing (equal). Nicole Schindler: Formal analysis (supporting); Investigation (lead); Methodology (supporting); Writing‐review & editing (equal). François Buscot: Methodology (equal); Resources (equal); Writing‐review & editing (equal). Nico Eisenhauer: Project administration (equal); Resources (equal); Writing‐review & editing (equal). Olga Ferlian: Project administration (equal); Writing‐review & editing (equal). Luis Daniel Prada Salcedo: Data curation (equal); Formal analysis (equal); Methodology (equal); Supervision (equal); Validation (equal); Writing‐review & editing (equal). Helge Bruelheide: Conceptualization (equal); Formal analysis (equal); Funding acquisition (lead); Project administration (equal); Resources (equal); Supervision (equal); Visualization (lead); Writing‐original draft (lead); Writing‐review & editing (equal).

## Supporting information

Appendix S1

## Data Availability

The sequencing data generated for this study were submitted to the European Nucleotide Archive (ENA) under the accession number PRJEB38894.
