# Supplementary material for: Mixing tree species associated with arbuscular or ectotrophic mycorrhizae reveals dual mycorrhization and interactive effects on the fungal partners
Source: Ecol Evol. 2021 Apr 2;11(10):5424–40. doi: 10.1002/ece3.7437 (PMC8131788; doi:10.1002/ece3.7437)
Supplement: Supplementary file 1 — Appendix S1 [file ECE3-11-5424-s001.docx]

Supplementary Material for the article in Ecology & Evolution

**Mixing tree species associated with arbuscular or ectotrophic mycorrhizae reveals dual mycorrhization and interactive effects on the fungal partners**

Heklau, H., Schindler, N., Buscot, F., Eisenhauer, N., Ferlian, O., Prada Salcedo, L.D. & Bruelheide, H.

Appendix Table S1: Number of samples analysed per tree species, separately by their preferred mycorrhiza type (AM, EM) and diversity level of the plots in which they were taken.

| Abbre-viation | Species | Monocultures | | | 2-species combinations | | | | 4-species combinations | | | |
| --- | --- | --- | --- | --- | --- | --- | --- | --- | --- | --- | --- | --- |
|  |  | AM | EM | AM | | EM | EM+AM | AM | | EM | EM+AM |  |
| Ac | Acer pseudoplatanus L. | 6 |  | 3 | |  | 3 | 4 | |  | 4 |  |
| Ae | Aesculus hippocastanum L. | 0 |  | 1 | |  | 1 | 4 | |  | 1 |  |
| Fr | Fraxinus excelsiorL. | 0 |  | 0 | |  | 2 | 3 | |  | 1 |  |
| Pr | Prunus avium L. | 6 |  | 2 | |  | 4 | 3 | |  | 4 |  |
| So | Sorbus aucuparia L. | 0 |  | 1 | |  | 1 | 3 | |  | 2 |  |
| Be | Betula pendula Roth. |  | 0 |  | | 1 | 2 |  | | 3 | 2 |  |
| Ca | Carpinus betulusL. |  | 6 |  | | 4 | 2 |  | | 5 | 3 |  |
| Fa | Fagus sylvatica L. |  | 6 |  | | 2 | 3 |  | | 5 | 3 |  |
| Qu | Quercus petraea (Matt.) Liebl. |  | 0 |  | | 0 | 0 |  | | 3 | 3 |  |
| Ti | Tilia platyphyllosScop. |  | 0 |  | | 1 | 2 |  | | 4 | 1 |  |

Appendix Table S2: OTUs enriched in tree species with the preferred mycorrhiza type AM (positive log_2_ fold changes) as opposed to EM (negative log_2_ fold changes). OTUs are arranged according to log_2_ fold changes (i.e. in descending affinity to AM trees and increasing affinity to EM trees).

| **OTU** | **Fungal species** | **Phylum** | **log10 mean of normalized counts** | **log2 fold changes** |
| --- | --- | --- | --- | --- |
| **OTUs enriched in AM trees** | |  |  |  |
| Otu00262 | Agaricomycetes_unclassified | Basidiomycota | 0.837 | 24.093 |
| Otu00159 | Psathyrella_corrugis | Basidiomycota | 0.705 | 24.015 |
| Otu00067 | Stephanosporaceae_sp | Basidiomycota | 1.972 | 10.966 |
| Otu00084 | Stephanosporaceae_sp | Basidiomycota | 1.905 | 10.033 |
| Otu00183 | Glomus_sp | Glomeromycota | 1.379 | 9.436 |
| Otu00113 | Entoloma_lanicum | Basidiomycota | 0.917 | 9.019 |
| Otu00150 | Rhizophagus_irregularis | Glomeromycota | 1.669 | 8.349 |
| Otu00061 | Pulvinula_sp | Ascomycota | 2.066 | 6.458 |
| Otu00127 | Thielaviopsis_basicola | Ascomycota | 1.666 | 6.208 |
| Otu00085 | Glomeraceae_sp | Glomeromycota | 1.971 | 5.861 |
| Otu00275 | Glomeraceae_sp | Glomeromycota | 1.095 | 5.770 |
| Otu00147 | Septoglomus_viscosum | Glomeromycota | 1.608 | 5.550 |
| Otu00174 | Claroideoglomus_sp | Glomeromycota | 1.484 | 5.505 |
| Otu00047 | Glomus_sp | Glomeromycota | 2.173 | 5.460 |
| Otu00064 | Ascomycota_unclassified | Ascomycota | 1.408 | 5.392 |
| Otu00259 | Septoglomus_viscosum | Glomeromycota | 1.155 | 5.385 |
| Otu00182 | Septoglomus_constrictum | Glomeromycota | 1.499 | 5.353 |
| Otu00212 | Claroideoglomeraceae_unclassified | Glomeromycota | 1.310 | 5.067 |
| Otu00149 | Glomus_compressum | Glomeromycota | 0.973 | 4.958 |
| Otu00063 | Rhizophagus_unclassified | Glomeromycota | 2.022 | 4.822 |
| Otu00109 | Claroideoglomus_sp | Glomeromycota | 1.790 | 4.804 |
| Otu00327 | Claroideoglomus_claroideum | Glomeromycota | 0.806 | 4.652 |
| Otu00358 | Pulvinula_sp | Ascomycota | 0.768 | 4.628 |
| Otu00367 | Glomus_sp | Glomeromycota | 0.798 | 4.575 |
| Otu00257 | Septoglomus_constrictum | Glomeromycota | 1.144 | 4.518 |
| Otu00215 | Rhizophagus_intraradices | Glomeromycota | 0.982 | 4.490 |
| Otu00264 | Fungi_unclassified | Fungi_unclassified | 0.866 | 4.457 |
| Otu00282 | Septoglomus_viscosum | Glomeromycota | 1.214 | 4.403 |
| Otu00355 | Claroideoglomus_sp | Glomeromycota | 0.874 | 4.388 |
| Otu00235 | Glomus_unclassified | Glomeromycota | 0.856 | 4.327 |
| Otu00168 | Glomeraceae_unclassified | Glomeromycota | 1.278 | 4.319 |
| Otu00190 | Septoglomus_unclassified | Glomeromycota | 1.540 | 4.234 |
| Otu00121 | Rhizophagus_irregularis | Glomeromycota | 1.663 | 4.200 |
| Otu00240 | Septoglomus_viscosum | Glomeromycota | 1.405 | 4.193 |
| Otu00171 | Glomeraceae_sp | Glomeromycota | 1.282 | 4.167 |
| Otu00383 | Diversisporaceae_sp | Glomeromycota | 0.562 | 4.146 |
| Otu00202 | Diversisporaceae_sp | Glomeromycota | 1.321 | 4.094 |
| Otu00315 | Glomus_sp | Glomeromycota | 1.014 | 4.066 |
| Otu00027 | Agaricales_unclassified | Basidiomycota | 2.453 | 4.034 |
| Otu00218 | Glomus_sp | Glomeromycota | 1.422 | 4.026 |
| Otu00112 | Sebacinales_sp | Basidiomycota | 1.766 | 3.893 |
| Otu00049 | Rhizophagus_irregularis | Glomeromycota | 2.345 | 3.891 |
| Otu00237 | Glomus_sp | Glomeromycota | 0.428 | 3.785 |
| Otu00366 | Glomeraceae_sp | Glomeromycota | 0.746 | 3.766 |
| Otu00356 | Fungi_unclassified | Fungi_unclassified | 0.518 | 3.715 |
| Otu00211 | Glomus_sp | Glomeromycota | 1.365 | 3.680 |
| Otu00390 | Paraglomerales_sp | Glomeromycota | 0.624 | 3.668 |
| Otu00398 | Diversisporaceae_sp | Glomeromycota | 0.493 | 3.629 |
| Otu00334 | Glomus_sp | Glomeromycota | 0.684 | 3.535 |
| Otu00283 | Glomeraceae_unclassified | Glomeromycota | 1.049 | 3.494 |
| Otu00565 | Rhizophagus_irregularis | Glomeromycota | 0.391 | 3.386 |
| Otu00393 | Glomeraceae_sp | Glomeromycota | 0.293 | 3.310 |
| Otu00511 | Septoglomus_viscosum | Glomeromycota | 0.352 | 3.255 |
| Otu00345 | Rhizophagus_irregularis | Glomeromycota | 0.879 | 3.052 |
| Otu00332 | Glomus_sp | Glomeromycota | 0.435 | 3.051 |
| Otu00091 | Ascomycota_sp | Ascomycota | 1.896 | 3.004 |
| Otu00416 | Fungi_unclassified | Fungi_unclassified | 0.339 | 2.932 |
| Otu00189 | Glomus_sp | Glomeromycota | 1.569 | 2.906 |
| Otu00472 | Diversisporaceae_sp | Glomeromycota | 0.200 | 2.904 |
| Otu00090 | Agaricales_unclassified | Basidiomycota | 1.862 | 2.818 |
| Otu00092 | Glomus_sp | Glomeromycota | 1.946 | 2.817 |
| Otu00526 | Leptosphaeria_veronicae | Ascomycota | 0.238 | 2.794 |
| Otu00080 | Glomeraceae_unclassified | Glomeromycota | 2.068 | 2.505 |
| Otu00011 | Rhizophagus_irregularis | Glomeromycota | 3.116 | 2.478 |
| Otu00021 | Rhizophagus_irregularis | Glomeromycota | 2.875 | 2.474 |
| Otu00037 | Glomus_sp | Glomeromycota | 2.489 | 2.434 |
| Otu00015 | Rhizophagus_irregularis | Glomeromycota | 3.026 | 2.427 |
| Otu00012 | Rhizophagus_irregularis | Glomeromycota | 3.115 | 2.338 |
| Otu00017 | Rhizophagus_irregularis | Glomeromycota | 3.068 | 2.280 |
| Otu00065 | Glomeraceae_unclassified | Glomeromycota | 2.168 | 2.274 |
| Otu00014 | Rhizophagus_irregularis | Glomeromycota | 3.092 | 2.196 |
| Otu00619 | Rhizophagus_irregularis | Glomeromycota | 0.143 | 2.174 |
| Otu00050 | Rhizophagus_irregularis | Glomeromycota | 2.233 | 2.169 |
| Otu00122 | Myrmecridium_unclassified | Ascomycota | 1.602 | 2.151 |
| Otu00039 | Leotiomycetes_unclassified | Ascomycota | 2.420 | 1.666 |
| Otu00025 | Cyphellophora_sp | Ascomycota | 2.510 | 1.341 |
| Otu00031 | Pleosporales_unclassified | Ascomycota | 2.359 | 1.074 |
| Otu00056 | Xylariales_sp | Ascomycota | 2.128 | 0.847 |
| Otu00018 | Xylariales_unclassified | Ascomycota | 2.883 | 0.501 |
| **OTUs enriched in EM trees** | |  |  |  |
| Otu00002 | Ilyonectria_macrodidyma | Ascomycota | 3.561 | -0.630 |
| Otu00010 | Exophiala_unclassified | Ascomycota | 3.213 | -0.825 |
| Otu00070 | Mortierella_elongata | Zygomycota | 2.210 | -0.845 |
| Otu00103 | Dendryphion_nanum | Ascomycota | 1.390 | -0.852 |
| Otu00004 | Helotiales_unclassified | Ascomycota | 3.257 | -0.938 |
| Otu00258 | Nectria_ramulariae | Ascomycota | 1.293 | -1.123 |
| Otu00252 | Chaetomiaceae_unclassified | Ascomycota | 1.343 | -1.293 |
| Otu00201 | Pseudogymnoascus_roseus | Ascomycota | 1.494 | -1.405 |
| Otu00176 | Mortierella_alpina | Zygomycota | 1.783 | -1.425 |
| Otu00066 | Mycosphaerella_tassiana | Ascomycota | 2.293 | -1.446 |
| Otu00247 | Malassezia_globosa | Basidiomycota | 0.717 | -1.505 |
| Otu00479 | Cylindrocarpon_sp | Ascomycota | 0.574 | -1.512 |
| Otu00267 | Cryptococcus_carnescens | Basidiomycota | 0.955 | -1.603 |
| Otu00677 | Mortierella_gamsii | Zygomycota | 0.270 | -1.630 |
| Otu00227 | Gibberella_zeae | Ascomycota | 1.518 | -1.649 |
| Otu00249 | Schizothecium_glutinans | Ascomycota | 1.442 | -1.697 |
| Otu00157 | Exophiala_opportunistica | Ascomycota | 1.650 | -1.758 |
| Otu00297 | Pyrenochaeta_inflorescentiae | Ascomycota | 0.644 | -1.940 |
| Otu00146 | Mortierella_alpina | Zygomycota | 1.899 | -1.941 |
| Otu00079 | Talaromyces_aculeatus | Ascomycota | 2.086 | -2.006 |
| Otu00052 | Strumella_sp | Ascomycota | 2.340 | -2.044 |
| Otu00256 | Mortierella_hyalina | Zygomycota | 1.372 | -2.060 |
| Otu00633 | Plectosphaerella_alismatis | Ascomycota | 0.539 | -2.220 |
| Otu00020 | Oidiodendron_unclassified | Ascomycota | 2.862 | -2.475 |
| Otu01239 | Mortierella_gamsii | Zygomycota | -0.474 | -2.503 |
| Otu00302 | Sordariomycetes_unclassified | Ascomycota | 1.316 | -2.505 |
| Otu00955 | Ilyonectria_macrodidyma | Ascomycota | 0.390 | -2.696 |
| Otu00520 | Acremonium_psammosporum | Ascomycota | 0.360 | -2.739 |
| Otu00674 | Ramicandelaber_sp | Zygomycota | 0.396 | -2.751 |
| Otu00844 | Rhodotorula_mucilaginosa | Basidiomycota | 0.186 | -2.793 |
| Otu00649 | Paxillus_unclassified | Basidiomycota | 0.266 | -2.897 |
| Otu00013 | Hebeloma_mesophaeum | Basidiomycota | 3.296 | -2.953 |
| Otu00251 | Agaricomycetes_unclassified | Basidiomycota | 0.685 | -2.967 |
| Otu00242 | Ilyonectria_mors-panacis | Ascomycota | 1.200 | -3.001 |
| Otu00593 | Mortierella_sp | Zygomycota | 0.608 | -3.030 |
| Otu00689 | Acremonium_stromaticum | Ascomycota | 0.285 | -3.156 |
| Otu00505 | Malassezia_restricta | Basidiomycota | 0.397 | -3.331 |
| Otu01317 | Tuber_sp | Ascomycota | -0.041 | -3.337 |
| Otu00053 | Fungi_unclassified | Fungi_unclassified | 1.174 | -3.558 |
| Otu01085 | Fungi_unclassified | Fungi_unclassified | -0.285 | -3.582 |
| Otu00830 | Exophiala_sp | Ascomycota | 0.176 | -3.612 |
| Otu00404 | Incertae_sedis_unclassified | Basidiomycota | 0.262 | -3.696 |
| Otu00206 | Scytalidium_circinatum | Ascomycota | 1.110 | -3.765 |
| Otu00966 | Tuber_sp | Ascomycota | 0.246 | -3.801 |
| Otu00534 | Fungi_unclassified | Fungi_unclassified | 0.559 | -3.867 |
| Otu00038 | Hebeloma_sp | Basidiomycota | 1.847 | -3.884 |
| Otu00314 | Fungi_unclassified | Fungi_unclassified | 0.684 | -3.901 |
| Otu00497 | Pulvinula_sp | Ascomycota | 0.038 | -3.932 |
| Otu00148 | Scleroderma_areolatum | Basidiomycota | 0.543 | -3.972 |
| Otu00750 | Tuber_unclassified | Ascomycota | 0.205 | -3.989 |
| Otu00125 | Fungi_unclassified | Fungi_unclassified | 0.462 | -4.010 |
| Otu00191 | Leucoagaricus_leucothites | Basidiomycota | 0.542 | -4.015 |
| Otu00096 | Tomentella_sp | Basidiomycota | 1.253 | -4.106 |
| Otu00535 | Chaetosphaeria_vermicularioides | Ascomycota | 0.204 | -4.128 |
| Otu00448 | Scleroderma_verrucosum | Basidiomycota | 0.146 | -4.169 |
| Otu00312 | Pezizaceae_sp | Ascomycota | 0.140 | -4.186 |
| Otu00732 | Fungi_unclassified | Fungi_unclassified | 0.074 | -4.202 |
| Otu00449 | Sporothrix_inflata | Ascomycota | 0.048 | -4.247 |
| Otu00447 | Pulvinula_sp | Ascomycota | 0.081 | -4.270 |
| Otu00865 | Fungi_unclassified | Fungi_unclassified | -0.100 | -4.352 |
| Otu00461 | Fungi_unclassified | Fungi_unclassified | 0.529 | -4.427 |
| Otu00540 | Fungi_unclassified | Fungi_unclassified | 0.028 | -4.436 |
| Otu00395 | Cadophora_finlandica | Ascomycota | 0.270 | -4.488 |
| Otu00644 | Fungi_unclassified | Fungi_unclassified | 0.120 | -4.644 |
| Otu00019 | Hebeloma_sp | Basidiomycota | 2.831 | -4.663 |
| Otu00073 | Oidiodendron_rhodogenum | Ascomycota | 0.571 | -4.754 |
| Otu00878 | Scleroderma_areolatum | Basidiomycota | 0.165 | -4.818 |
| Otu00826 | Tuber_sp | Ascomycota | 0.539 | -4.827 |
| Otu00165 | Melanogaster_variegatus | Basidiomycota | 0.422 | -4.849 |
| Otu00210 | Pezizaceae_sp | Ascomycota | 0.332 | -4.881 |
| Otu00446 | Fungi_unclassified | Fungi_unclassified | 0.600 | -4.966 |
| Otu00509 | Fungi_unclassified | Fungi_unclassified | 0.592 | -4.999 |
| Otu00276 | Talaromyces_unclassified | Ascomycota | 0.499 | -5.021 |
| Otu00585 | Fungi_unclassified | Fungi_unclassified | 0.151 | -5.023 |
| Otu00153 | Ilyonectria_robusta | Ascomycota | 1.689 | -5.025 |
| Otu00451 | Fungi_unclassified | Fungi_unclassified | 0.380 | -5.034 |
| Otu00207 | Fungi_unclassified | Fungi_unclassified | 1.585 | -5.174 |
| Otu00114 | Strophariaceae_unclassified | Basidiomycota | 1.128 | -5.224 |
| Otu00026 | Peziza_michelii | Ascomycota | 1.205 | -5.250 |
| Otu00307 | Tomentella_sp | Basidiomycota | 0.548 | -5.440 |
| Otu00277 | Helotiales_unclassified | Ascomycota | 0.900 | -5.478 |
| Otu00134 | Alnicola_macrospora | Basidiomycota | 1.183 | -5.479 |
| Otu00399 | Hyphopichia_burtonii | Ascomycota | 0.587 | -5.551 |
| Otu00099 | Geopora_cervina | Ascomycota | 0.727 | -5.584 |
| Otu00045 | Geopora_cervina | Ascomycota | 0.683 | -5.868 |
| Otu00293 | Agaricomycetes_unclassified | Basidiomycota | 0.953 | -6.058 |
| Otu00194 | Fungi_unclassified | Fungi_unclassified | 0.938 | -6.079 |
| Otu00400 | Fungi_sp | unclassified_Fungi | 0.584 | -6.289 |
| Otu00074 | Fungi_unclassified | Fungi_unclassified | 1.833 | -6.461 |
| Otu00319 | Incertae_sedis_unclassified | Ascomycota | 0.772 | -6.579 |
| Otu00405 | Mortierella_dichotoma | Zygomycota | 0.803 | -6.581 |
| Otu00162 | Fungi_sp | unclassified_Fungi | 1.162 | -7.035 |
| Otu00102 | Tomentella_ellisii | Basidiomycota | 1.089 | -7.426 |
| Otu00051 | Ascomycota_unclassified | Ascomycota | 2.902 | -7.661 |
| Otu00679 | Tuber_sp | Ascomycota | 1.241 | -7.987 |
| Otu00128 | Fungi_unclassified | Fungi_unclassified | 1.429 | -8.164 |
| Otu00083 | Incertae_sedis_unclassified | Basidiomycota | 1.680 | -10.028 |
| Otu00223 | Sebacinales_unclassified | Basidiomycota | 1.598 | -10.052 |

Appendix Table S3: OTUs significantly enriched in each of the 10 target tree species (positive log_2_ fold changes) as opposed to the other nine tree species (negative log_2_ fold changes, i.e. depleted in that target species). OTUs are arranged according to log_2_ fold changes (i.e. in descending affinity to the respective target tree species).

| **Tree species** | **OTU** | **Fungal species** | **Phylum** | **log10 mean of normalized counts** | **log2 fold changes** |
| --- | --- | --- | --- | --- | --- |
| **OTUs significantly enriched or depleted in Acer pseudoplatanus** | |  |  |  |  |
| Acer pseudoplatanus | Otu00122 | Myrmecridium_unclassified | Ascomycota | 1.602 | 3.508 |
| Acer pseudoplatanus | Otu00091 | Ascomycota_sp | Ascomycota | 1.896 | 3.334 |
| Acer pseudoplatanus | Otu00112 | Sebacinales_sp | Basidiomycota | 1.766 | 3.295 |
| Acer pseudoplatanus | Otu00366 | Glomeraceae_sp | Glomeromycota | 0.530 | 3.267 |
| Acer pseudoplatanus | Otu00398 | Diversisporaceae_sp | Glomeromycota | 0.379 | 3.144 |
| Acer pseudoplatanus | Otu00090 | Agaricales_unclassified | Basidiomycota | 1.862 | 2.740 |
| Acer pseudoplatanus | Otu00085 | Glomeraceae_sp | Glomeromycota | 1.971 | 2.176 |
| Acer pseudoplatanus | Otu00057 | Microdochium_bolleyi | Ascomycota | 2.092 | 1.491 |
| Acer pseudoplatanus | Otu00056 | Xylariales_sp | Ascomycota | 2.128 | 1.308 |
| Acer pseudoplatanus | Otu00002 | Ilyonectria_macrodidyma | Ascomycota | 3.561 | -0.458 |
| Acer pseudoplatanus | Otu00010 | Exophiala_unclassified | Ascomycota | 3.213 | -0.725 |
| Acer pseudoplatanus | Otu00146 | Mortierella_alpina | Zygomycota | 1.899 | -1.189 |
| Acer pseudoplatanus | Otu00039 | Leotiomycetes_unclassified | Ascomycota | 2.420 | -1.296 |
| Acer pseudoplatanus | Otu00052 | Strumella_sp | Ascomycota | 2.311 | -1.621 |
| Acer pseudoplatanus | Otu00094 | Paraphoma_unclassified | Ascomycota | 1.569 | -1.834 |
| Acer pseudoplatanus | Otu00247 | Malassezia_globosa | Basidiomycota | 0.717 | -2.035 |
| Acer pseudoplatanus | Otu00157 | Exophiala_opportunistica | Ascomycota | 1.650 | -2.038 |
| Acer pseudoplatanus | Otu00408 | Acremonium_persicinum | Ascomycota | 0.723 | -2.092 |
| Acer pseudoplatanus | Otu00079 | Talaromyces_aculeatus | Ascomycota | 1.982 | -2.515 |
| Acer pseudoplatanus | Otu00013 | Hebeloma_mesophaeum | Basidiomycota | 3.005 | -2.523 |
| Acer pseudoplatanus | Otu00071 | Auriculariales_unclassified | Basidiomycota | 1.491 | -2.709 |
| Acer pseudoplatanus | Otu00153 | Ilyonectria_robusta | Ascomycota | 1.115 | -2.726 |
| Acer pseudoplatanus | Otu00221 | Geopyxis_unclassified | Ascomycota | 0.977 | -2.913 |
| Acer pseudoplatanus | Otu00344 | Staphylotrichum_boninense | Ascomycota | 0.975 | -2.989 |
| Acer pseudoplatanus | Otu00271 | Phaeoacremonium_hungaricum | Ascomycota | 1.064 | -3.036 |
| Acer pseudoplatanus | Otu00966 | Tuber_sp | Ascomycota | 0.246 | -3.228 |
| Acer pseudoplatanus | Otu00471 | Acremonium_alternatum | Ascomycota | 0.704 | -3.350 |
| Acer pseudoplatanus | Otu00161 | Agaricomycetes_unclassified | Basidiomycota | 0.724 | -3.412 |
| Acer pseudoplatanus | Otu00192 | Incertae_sedis_unclassified | Ascomycota | 1.104 | -3.928 |
| Acer pseudoplatanus | Otu00505 | Malassezia_restricta | Basidiomycota | 0.397 | -3.978 |
| Acer pseudoplatanus | Otu00480 | Stachybotrys_elegans | Ascomycota | 0.157 | -3.990 |
| Acer pseudoplatanus | Otu00207 | Fungi_unclassified | Fungi_unclassified | 1.372 | -4.070 |
| Acer pseudoplatanus | Otu00277 | Helotiales_unclassified | Ascomycota | 0.799 | -4.115 |
| Acer pseudoplatanus | Otu00231 | Auriculariales_sp | Basidiomycota | 0.552 | -4.180 |
| Acer pseudoplatanus | Otu00403 | Aureobasidium_pullulans | Ascomycota | 0.482 | -4.262 |
| Acer pseudoplatanus | Otu00826 | Tuber_sp | Ascomycota | 0.539 | -4.283 |
| Acer pseudoplatanus | Otu00170 | Ceratobasidiaceae_sp | Basidiomycota | 1.623 | -4.287 |
| Acer pseudoplatanus | Otu00633 | Plectosphaerella_alismatis | Ascomycota | 0.525 | -4.376 |
| Acer pseudoplatanus | Otu00149 | Glomus_compressum | Glomeromycota | 0.456 | -4.429 |
| Acer pseudoplatanus | Otu00019 | Hebeloma_sp | Basidiomycota | 2.590 | -4.576 |
| Acer pseudoplatanus | Otu00598 | Cladosporium_unclassified | Ascomycota | 0.422 | -4.786 |
| Acer pseudoplatanus | Otu00534 | Fungi_unclassified | Fungi_unclassified | 0.439 | -4.835 |
| Acer pseudoplatanus | Otu00040 | Flagelloscypha_minutissima | Basidiomycota | 2.364 | -4.920 |
| Acer pseudoplatanus | Otu00045 | Geopora_cervina | Ascomycota | 0.687 | -4.986 |
| Acer pseudoplatanus | Otu00238 | Scleroderma_verrucosum | Basidiomycota | 0.458 | -5.060 |
| Acer pseudoplatanus | Otu00527 | Fungi_sp | unclassified_Fungi | 0.336 | -5.114 |
| Acer pseudoplatanus | Otu00038 | Hebeloma_sp | Basidiomycota | 1.625 | -5.158 |
| Acer pseudoplatanus | Otu00549 | Fungi_unclassified | Fungi_unclassified | 0.364 | -5.205 |
| Acer pseudoplatanus | Otu00191 | Leucoagaricus_leucothites | Basidiomycota | 0.617 | -5.229 |
| Acer pseudoplatanus | Otu00026 | Peziza_michelii | Ascomycota | 1.205 | -5.242 |
| Acer pseudoplatanus | Otu00140 | Phialocephala_fortinii | Ascomycota | 0.404 | -5.257 |
| Acer pseudoplatanus | Otu00034 | Hebeloma_sp | Basidiomycota | 0.479 | -5.259 |
| Acer pseudoplatanus | Otu00237 | Glomus_sp | Glomeromycota | 0.428 | -5.263 |
| Acer pseudoplatanus | Otu00099 | Geopora_cervina | Ascomycota | 1.170 | -5.478 |
| Acer pseudoplatanus | Otu00507 | Fungi_unclassified | Fungi_unclassified | 0.489 | -5.592 |
| Acer pseudoplatanus | Otu00276 | Talaromyces_unclassified | Ascomycota | 0.531 | -5.726 |
| Acer pseudoplatanus | Otu00046 | Agaricomycetes_unclassified | Basidiomycota | 0.882 | -5.877 |
| Acer pseudoplatanus | Otu00314 | Fungi_unclassified | Fungi_unclassified | 0.684 | -5.980 |
| Acer pseudoplatanus | Otu00188 | Pezizaceae_sp | Ascomycota | 0.729 | -6.066 |
| Acer pseudoplatanus | Otu00156 | Auricularia_unclassified | Basidiomycota | 0.802 | -6.155 |
| Acer pseudoplatanus | Otu00148 | Scleroderma_areolatum | Basidiomycota | 0.920 | -6.274 |
| Acer pseudoplatanus | Otu00051 | Ascomycota_unclassified | Ascomycota | 2.498 | -6.346 |
| Acer pseudoplatanus | Otu00400 | Fungi_sp | unclassified_Fungi | 0.762 | -6.398 |
| Acer pseudoplatanus | Otu00286 | Serendipita_sp | Basidiomycota | 0.752 | -6.428 |
| Acer pseudoplatanus | Otu00319 | Incertae_sedis_unclassified | Ascomycota | 0.772 | -6.494 |
| Acer pseudoplatanus | Otu00020 | Oidiodendron_unclassified | Ascomycota | 2.807 | -6.799 |
| Acer pseudoplatanus | Otu00194 | Fungi_unclassified | Fungi_unclassified | 0.938 | -6.851 |
| Acer pseudoplatanus | Otu00074 | Fungi_unclassified | Fungi_unclassified | 1.388 | -6.992 |
| Acer pseudoplatanus | Otu00679 | Tuber_sp | Ascomycota | 1.239 | -7.754 |
| Acer pseudoplatanus | Otu00102 | Tomentella_ellisii | Basidiomycota | 1.089 | -7.810 |
| Acer pseudoplatanus | Otu00113 | Entoloma_lanicum | Basidiomycota | 1.001 | -7.861 |
| Acer pseudoplatanus | Otu00223 | Sebacinales_unclassified | Basidiomycota | 1.478 | -8.801 |
| Acer pseudoplatanus | Otu00232 | Flagelloscypha_minutissima | Basidiomycota | 0.839 | -9.057 |
| Acer pseudoplatanus | Otu00128 | Fungi_unclassified | Fungi_unclassified | 1.075 | -9.969 |
| Acer pseudoplatanus | Otu00083 | Incertae_sedis_unclassified | Basidiomycota | 1.702 | -10.588 |
| Acer pseudoplatanus | Otu00053 | Fungi_unclassified | Fungi_unclassified | 1.652 | -10.748 |
| Acer pseudoplatanus | Otu00162 | Fungi_sp | unclassified_Fungi | 1.167 | -23.701 |
| **OTUs significantly enriched or depleted in Aesculus hippocastanum** | |  |  |  |  |
| Aesculus hippocastanum | Otu00241 | Acremonium_implicatum | Ascomycota | 0.955 | 10.709 |
| Aesculus hippocastanum | Otu00145 | Sarocladium_sp | Ascomycota | 1.393 | 7.603 |
| Aesculus hippocastanum | Otu00075 | Fusarium_unclassified | Ascomycota | 2.108 | -1.423 |
| Aesculus hippocastanum | Otu00160 | Fusarium_solani | Ascomycota | 1.650 | -1.609 |
| Aesculus hippocastanum | Otu00181 | Pyrenochaetopsis_leptospora | Ascomycota | 1.507 | -2.086 |
| Aesculus hippocastanum | Otu00214 | Fusicolla_unclassified | Ascomycota | 1.447 | -2.331 |
| Aesculus hippocastanum | Otu00157 | Exophiala_opportunistica | Ascomycota | 1.650 | -2.344 |
| Aesculus hippocastanum | Otu00258 | Nectria_ramulariae | Ascomycota | 1.293 | -2.377 |
| Aesculus hippocastanum | Otu00245 | Gibellulopsis_nigrescens | Ascomycota | 1.258 | -2.517 |
| Aesculus hippocastanum | Otu00176 | Mortierella_alpina | Zygomycota | 1.674 | -2.613 |
| Aesculus hippocastanum | Otu00169 | Sordariomycetes_unclassified | Ascomycota | 1.639 | -2.681 |
| Aesculus hippocastanum | Otu00180 | Pleosporales_sp | Ascomycota | 1.541 | -2.725 |
| Aesculus hippocastanum | Otu00025 | Cyphellophora_sp | Ascomycota | 2.510 | -2.878 |
| Aesculus hippocastanum | Otu00146 | Mortierella_alpina | Zygomycota | 1.899 | -3.046 |
| Aesculus hippocastanum | Otu00098 | Clonostachys_rosea | Ascomycota | 1.703 | -3.087 |
| Aesculus hippocastanum | Otu00272 | Mortierella_alpina | Zygomycota | 1.191 | -3.116 |
| Aesculus hippocastanum | Otu00110 | Pyrenochaetopsis_leptospora | Ascomycota | 1.718 | -3.299 |
| Aesculus hippocastanum | Otu00256 | Mortierella_hyalina | Zygomycota | 1.309 | -3.371 |
| Aesculus hippocastanum | Otu00112 | Sebacinales_sp | Basidiomycota | 1.427 | -3.392 |
| Aesculus hippocastanum | Otu00052 | Strumella_sp | Ascomycota | 2.340 | -3.656 |
| Aesculus hippocastanum | Otu00133 | Bartalinia_robillardoides | Ascomycota | 1.113 | -3.847 |
| Aesculus hippocastanum | Otu00193 | Slopeiomyces_cylindrosporus | Ascomycota | 1.588 | -3.859 |
| Aesculus hippocastanum | Otu00419 | Metarhizium_marquandii | Ascomycota | 0.564 | -3.914 |
| Aesculus hippocastanum | Otu00333 | Ascomycota_unclassified | Ascomycota | 0.888 | -4.077 |
| Aesculus hippocastanum | Otu00966 | Tuber_sp | Ascomycota | 0.246 | -4.142 |
| Aesculus hippocastanum | Otu00019 | Hebeloma_sp | Basidiomycota | 2.591 | -4.166 |
| Aesculus hippocastanum | Otu00371 | Pleosporales_unclassified | Ascomycota | 0.600 | -4.202 |
| Aesculus hippocastanum | Otu00252 | Chaetomiaceae_unclassified | Ascomycota | 1.343 | -4.311 |
| Aesculus hippocastanum | Otu00028 | Geopora_tenuis | Ascomycota | 0.707 | -4.394 |
| Aesculus hippocastanum | Otu00379 | Gibellulopsis_nigrescens | Ascomycota | 0.686 | -4.402 |
| Aesculus hippocastanum | Otu00325 | Lasiosphaeriaceae_unclassified | Ascomycota | 0.934 | -4.406 |
| Aesculus hippocastanum | Otu00349 | Fusarium_solani | Ascomycota | 0.886 | -4.437 |
| Aesculus hippocastanum | Otu00049 | Rhizophagus_irregularis | Glomeromycota | 1.979 | -4.589 |
| Aesculus hippocastanum | Otu00669 | Ascomycota_unclassified | Ascomycota | 0.033 | -4.596 |
| Aesculus hippocastanum | Otu00826 | Tuber_sp | Ascomycota | 0.539 | -4.866 |
| Aesculus hippocastanum | Otu00518 | Phaeosphaeriaceae_unclassified | Ascomycota | 0.200 | -4.922 |
| Aesculus hippocastanum | Otu00259 | Septoglomus_viscosum | Glomeromycota | 0.706 | -5.078 |
| Aesculus hippocastanum | Otu00560 | Cyphellophora_laciniata | Ascomycota | 0.210 | -5.106 |
| Aesculus hippocastanum | Otu00257 | Septoglomus_constrictum | Glomeromycota | 1.029 | -5.115 |
| Aesculus hippocastanum | Otu00213 | Alternaria_tenuissima | Ascomycota | 1.407 | -5.159 |
| Aesculus hippocastanum | Otu00368 | Roussoella_unclassified | Ascomycota | 0.235 | -5.194 |
| Aesculus hippocastanum | Otu00633 | Plectosphaerella_alismatis | Ascomycota | 0.539 | -5.242 |
| Aesculus hippocastanum | Otu00253 | Naevala_sp | Ascomycota | 0.296 | -5.296 |
| Aesculus hippocastanum | Otu00520 | Acremonium_psammosporum | Ascomycota | 0.355 | -5.301 |
| Aesculus hippocastanum | Otu00450 | Chrysosporium_pseudomerdarium | Ascomycota | 0.649 | -5.334 |
| Aesculus hippocastanum | Otu00498 | Pleosporales_unclassified | Ascomycota | 0.320 | -5.411 |
| Aesculus hippocastanum | Otu00047 | Glomus_sp | Glomeromycota | 1.540 | -5.426 |
| Aesculus hippocastanum | Otu00534 | Fungi_unclassified | Fungi_unclassified | 0.453 | -5.429 |
| Aesculus hippocastanum | Otu00468 | Exophiala_opportunistica | Ascomycota | 0.365 | -5.496 |
| Aesculus hippocastanum | Otu00013 | Hebeloma_mesophaeum | Basidiomycota | 3.017 | -5.498 |
| Aesculus hippocastanum | Otu00297 | Pyrenochaeta_inflorescentiae | Ascomycota | 0.676 | -5.586 |
| Aesculus hippocastanum | Otu00424 | Cryptococcus_heimaeyensis | Basidiomycota | 0.786 | -5.586 |
| Aesculus hippocastanum | Otu00505 | Malassezia_restricta | Basidiomycota | 0.397 | -5.605 |
| Aesculus hippocastanum | Otu00483 | Mortierella_unclassified | Zygomycota | 0.556 | -5.641 |
| Aesculus hippocastanum | Otu00383 | Diversisporaceae_sp | Glomeromycota | 0.426 | -5.700 |
| Aesculus hippocastanum | Otu00495 | Pleosporales_unclassified | Ascomycota | 0.571 | -5.744 |
| Aesculus hippocastanum | Otu00240 | Septoglomus_viscosum | Glomeromycota | 0.982 | -5.765 |
| Aesculus hippocastanum | Otu00457 | Phialocephala_xalapensis | Ascomycota | 0.578 | -5.820 |
| Aesculus hippocastanum | Otu00289 | Chaetomium_unclassified | Ascomycota | 0.959 | -5.845 |
| Aesculus hippocastanum | Otu00174 | Claroideoglomus_sp | Glomeromycota | 1.220 | -5.896 |
| Aesculus hippocastanum | Otu00091 | Ascomycota_sp | Ascomycota | 1.650 | -5.920 |
| Aesculus hippocastanum | Otu00117 | Psathyrella_panaeoloides | Basidiomycota | 1.494 | -5.920 |
| Aesculus hippocastanum | Otu00034 | Hebeloma_sp | Basidiomycota | 0.486 | -5.942 |
| Aesculus hippocastanum | Otu00189 | Glomus_sp | Glomeromycota | 1.357 | -5.950 |
| Aesculus hippocastanum | Otu00430 | Darksidea_unclassified | Ascomycota | 0.507 | -6.000 |
| Aesculus hippocastanum | Otu00282 | Septoglomus_viscosum | Glomeromycota | 0.807 | -6.002 |
| Aesculus hippocastanum | Otu00283 | Glomeraceae_unclassified | Glomeromycota | 0.935 | -6.022 |
| Aesculus hippocastanum | Otu00403 | Aureobasidium_pullulans | Ascomycota | 0.515 | -6.035 |
| Aesculus hippocastanum | Otu00144 | Chalastospora_ellipsoidea | Ascomycota | 0.818 | -6.059 |
| Aesculus hippocastanum | Otu00287 | Sordariales_unclassified | Ascomycota | 1.051 | -6.062 |
| Aesculus hippocastanum | Otu00153 | Ilyonectria_robusta | Ascomycota | 1.115 | -6.150 |
| Aesculus hippocastanum | Otu00367 | Glomus_sp | Glomeromycota | 0.555 | -6.171 |
| Aesculus hippocastanum | Otu00445 | Hirsutella_vermicola | Ascomycota | 0.568 | -6.207 |
| Aesculus hippocastanum | Otu00231 | Auriculariales_sp | Basidiomycota | 0.580 | -6.255 |
| Aesculus hippocastanum | Otu00131 | Periconia_sp | Ascomycota | 1.299 | -6.267 |
| Aesculus hippocastanum | Otu00277 | Helotiales_unclassified | Ascomycota | 0.768 | -6.443 |
| Aesculus hippocastanum | Otu00020 | Oidiodendron_unclassified | Ascomycota | 2.861 | -6.465 |
| Aesculus hippocastanum | Otu00204 | Montagnulaceae_unclassified | Ascomycota | 0.779 | -6.470 |
| Aesculus hippocastanum | Otu00051 | Ascomycota_unclassified | Ascomycota | 2.499 | -6.517 |
| Aesculus hippocastanum | Otu00295 | Glomeraceae_sp | Glomeromycota | 0.969 | -6.526 |
| Aesculus hippocastanum | Otu00087 | Paxillus_unclassified | Basidiomycota | 1.605 | -6.681 |
| Aesculus hippocastanum | Otu00408 | Acremonium_persicinum | Ascomycota | 0.753 | -6.717 |
| Aesculus hippocastanum | Otu00139 | Glarea_unclassified | Ascomycota | 0.765 | -6.827 |
| Aesculus hippocastanum | Otu00321 | Schizothecium_inaequale | Ascomycota | 0.807 | -6.925 |
| Aesculus hippocastanum | Otu00250 | Coprinopsis_unclassified | Basidiomycota | 0.984 | -7.203 |
| Aesculus hippocastanum | Otu00207 | Fungi_unclassified | Fungi_unclassified | 1.434 | -7.233 |
| Aesculus hippocastanum | Otu00206 | Scytalidium_circinatum | Ascomycota | 0.924 | -7.354 |
| Aesculus hippocastanum | Otu00255 | Herpotrichiellaceae_unclassified | Ascomycota | 1.054 | -7.367 |
| Aesculus hippocastanum | Otu00129 | Lactarius_scoticus | Basidiomycota | 1.066 | -7.409 |
| Aesculus hippocastanum | Otu00251 | Agaricomycetes_unclassified | Basidiomycota | 0.980 | -7.534 |
| Aesculus hippocastanum | Otu00221 | Geopyxis_unclassified | Ascomycota | 0.991 | -7.548 |
| Aesculus hippocastanum | Otu00211 | Glomus_sp | Glomeromycota | 1.030 | -7.697 |
| Aesculus hippocastanum | Otu00099 | Geopora_cervina | Ascomycota | 1.170 | -7.739 |
| Aesculus hippocastanum | Otu00074 | Fungi_unclassified | Fungi_unclassified | 1.388 | -7.842 |
| Aesculus hippocastanum | Otu00026 | Peziza_michelii | Ascomycota | 1.227 | -7.930 |
| Aesculus hippocastanum | Otu00227 | Gibberella_zeae | Ascomycota | 1.389 | -7.964 |
| Aesculus hippocastanum | Otu00302 | Sordariomycetes_unclassified | Ascomycota | 1.154 | -8.061 |
| Aesculus hippocastanum | Otu00679 | Tuber_sp | Ascomycota | 1.241 | -8.182 |
| Aesculus hippocastanum | Otu00216 | Ceratobasidium_unclassified | Basidiomycota | 1.384 | -8.450 |
| Aesculus hippocastanum | Otu00058 | Calyptella_sp | Basidiomycota | 1.184 | -8.780 |
| Aesculus hippocastanum | Otu00067 | Stephanosporaceae_sp | Basidiomycota | 1.332 | -8.803 |
| Aesculus hippocastanum | Otu00086 | Subulicystidium_sp | Basidiomycota | 1.531 | -9.476 |
| Aesculus hippocastanum | Otu00079 | Talaromyces_aculeatus | Ascomycota | 1.996 | -9.677 |
| Aesculus hippocastanum | Otu00130 | Mycosphaerella_tassiana | Ascomycota | 1.432 | -9.954 |
| Aesculus hippocastanum | Otu00053 | Fungi_unclassified | Fungi_unclassified | 1.432 | -10.135 |
| Aesculus hippocastanum | Otu00032 | Tuber_maculatum | Ascomycota | 1.613 | -10.691 |
| Aesculus hippocastanum | Otu00040 | Flagelloscypha_minutissima | Basidiomycota | 2.256 | -10.775 |
| Aesculus hippocastanum | Otu00024 | Tuber_maculatum | Ascomycota | 1.777 | -11.177 |
| Aesculus hippocastanum | Otu00162 | Fungi_sp | unclassified_Fungi | 1.167 | -22.119 |
| Aesculus hippocastanum | Otu00084 | Stephanosporaceae_sp | Basidiomycota | 0.998 | -22.220 |
| Aesculus hippocastanum | Otu00128 | Fungi_unclassified | Fungi_unclassified | 1.079 | -22.471 |
| Aesculus hippocastanum | Otu00118 | Glarea_unclassified | Ascomycota | 1.269 | -23.091 |
| Aesculus hippocastanum | Otu00127 | Thielaviopsis_basicola | Ascomycota | 1.283 | -23.121 |
| Aesculus hippocastanum | Otu00170 | Ceratobasidiaceae_sp | Basidiomycota | 1.640 | -24.243 |
| Aesculus hippocastanum | Otu00083 | Incertae_sedis_unclassified | Basidiomycota | 1.702 | -24.436 |
| **OTUs significantly enriched or depleted in Fraxinus excelsior** | |  |  |  |  |
| Fraxinus excelsior | Otu00544 | Plectosphaerella_alismatis | Ascomycota | 0.434 | 27.900 |
| Fraxinus excelsior | Otu00478 | Pleosporales_unclassified | Ascomycota | 0.647 | 6.436 |
| Fraxinus excelsior | Otu00259 | Septoglomus_viscosum | Glomeromycota | 1.342 | 5.725 |
| Fraxinus excelsior | Otu00240 | Septoglomus_viscosum | Glomeromycota | 1.405 | 5.450 |
| Fraxinus excelsior | Otu00147 | Septoglomus_viscosum | Glomeromycota | 1.799 | 5.264 |
| Fraxinus excelsior | Otu00282 | Septoglomus_viscosum | Glomeromycota | 1.214 | 5.206 |
| Fraxinus excelsior | Otu00565 | Rhizophagus_irregularis | Glomeromycota | 0.391 | 4.400 |
| Fraxinus excelsior | Otu00190 | Septoglomus_unclassified | Glomeromycota | 1.540 | 4.296 |
| Fraxinus excelsior | Otu00382 | Ophiosphaerella_sp | Ascomycota | 0.816 | 3.648 |
| Fraxinus excelsior | Otu00021 | Rhizophagus_irregularis | Glomeromycota | 2.875 | 3.511 |
| Fraxinus excelsior | Otu00345 | Rhizophagus_irregularis | Glomeromycota | 0.879 | 3.502 |
| Fraxinus excelsior | Otu00105 | Sordariomycetes_unclassified | Ascomycota | 1.952 | 3.044 |
| Fraxinus excelsior | Otu00017 | Rhizophagus_irregularis | Glomeromycota | 3.068 | 2.969 |
| Fraxinus excelsior | Otu00012 | Rhizophagus_irregularis | Glomeromycota | 3.115 | 2.951 |
| Fraxinus excelsior | Otu00185 | Periconia_sp | Ascomycota | 1.566 | 2.925 |
| Fraxinus excelsior | Otu00011 | Rhizophagus_irregularis | Glomeromycota | 3.116 | 2.824 |
| Fraxinus excelsior | Otu00014 | Rhizophagus_irregularis | Glomeromycota | 3.092 | 2.773 |
| Fraxinus excelsior | Otu00015 | Rhizophagus_irregularis | Glomeromycota | 3.026 | 2.587 |
| Fraxinus excelsior | Otu00115 | Acremonium_nepalense | Ascomycota | 1.766 | 2.307 |
| Fraxinus excelsior | Otu00010 | Exophiala_unclassified | Ascomycota | 3.213 | -1.340 |
| Fraxinus excelsior | Otu00146 | Mortierella_alpina | Zygomycota | 1.899 | -1.953 |
| Fraxinus excelsior | Otu00070 | Mortierella_elongata | Zygomycota | 2.210 | -1.961 |
| Fraxinus excelsior | Otu00052 | Strumella_sp | Ascomycota | 2.340 | -2.749 |
| Fraxinus excelsior | Otu00081 | Ascomycota_unclassified | Ascomycota | 2.066 | -3.534 |
| Fraxinus excelsior | Otu00019 | Hebeloma_sp | Basidiomycota | 2.592 | -3.604 |
| Fraxinus excelsior | Otu00242 | Ilyonectria_mors-panacis | Ascomycota | 1.082 | -3.982 |
| Fraxinus excelsior | Otu00079 | Talaromyces_aculeatus | Ascomycota | 1.996 | -4.417 |
| Fraxinus excelsior | Otu00051 | Ascomycota_unclassified | Ascomycota | 2.498 | -5.414 |
| Fraxinus excelsior | Otu00047 | Glomus_sp | Glomeromycota | 1.540 | -5.597 |
| Fraxinus excelsior | Otu00040 | Flagelloscypha_minutissima | Basidiomycota | 2.256 | -6.797 |
| Fraxinus excelsior | Otu00020 | Oidiodendron_unclassified | Ascomycota | 2.862 | -7.394 |
| Fraxinus excelsior | Otu00083 | Incertae_sedis_unclassified | Basidiomycota | 1.702 | -8.317 |
| Fraxinus excelsior | Otu00118 | Glarea_unclassified | Ascomycota | 1.270 | -17.849 |
| Fraxinus excelsior | Otu00084 | Stephanosporaceae_sp | Basidiomycota | 1.001 | -19.105 |
| Fraxinus excelsior | Otu00102 | Tomentella_ellisii | Basidiomycota | 1.097 | -20.106 |
| Fraxinus excelsior | Otu00053 | Fungi_unclassified | Fungi_unclassified | 1.432 | -20.268 |
| Fraxinus excelsior | Otu00074 | Fungi_unclassified | Fungi_unclassified | 1.388 | -20.331 |
| Fraxinus excelsior | Otu00162 | Fungi_sp | unclassified_Fungi | 1.167 | -20.414 |
| **OTUs significantly enriched or depleted in Prunus avium** | |  |  |  |  |
| Prunus avium | Otu00067 | Stephanosporaceae_sp | Basidiomycota | 1.972 | 4.889 |
| Prunus avium | Otu00235 | Glomus_unclassified | Glomeromycota | 1.199 | 4.800 |
| Prunus avium | Otu00084 | Stephanosporaceae_sp | Basidiomycota | 1.905 | 4.741 |
| Prunus avium | Otu00264 | Fungi_unclassified | Fungi_unclassified | 1.181 | 4.509 |
| Prunus avium | Otu00168 | Glomeraceae_unclassified | Glomeromycota | 1.594 | 4.376 |
| Prunus avium | Otu00315 | Glomus_sp | Glomeromycota | 0.932 | 4.223 |
| Prunus avium | Otu00171 | Glomeraceae_sp | Glomeromycota | 1.589 | 4.204 |
| Prunus avium | Otu00049 | Rhizophagus_irregularis | Glomeromycota | 2.178 | 4.178 |
| Prunus avium | Otu00127 | Thielaviopsis_basicola | Ascomycota | 1.708 | 3.730 |
| Prunus avium | Otu00047 | Glomus_sp | Glomeromycota | 1.990 | 3.249 |
| Prunus avium | Otu00040 | Flagelloscypha_minutissima | Basidiomycota | 2.502 | 2.877 |
| Prunus avium | Otu00039 | Leotiomycetes_unclassified | Ascomycota | 2.420 | 2.730 |
| Prunus avium | Otu00799 | Ascomycota_unclassified | Ascomycota | -0.144 | 2.567 |
| Prunus avium | Otu00080 | Glomeraceae_unclassified | Glomeromycota | 2.068 | 2.422 |
| Prunus avium | Otu00027 | Agaricales_unclassified | Basidiomycota | 2.453 | 1.953 |
| Prunus avium | Otu00025 | Cyphellophora_sp | Ascomycota | 2.510 | 1.813 |
| Prunus avium | Otu00103 | Dendryphion_nanum | Ascomycota | 1.414 | -1.333 |
| Prunus avium | Otu00146 | Mortierella_alpina | Zygomycota | 1.899 | -1.335 |
| Prunus avium | Otu00052 | Strumella_sp | Ascomycota | 2.340 | -1.483 |
| Prunus avium | Otu00066 | Mycosphaerella_tassiana | Ascomycota | 2.129 | -1.542 |
| Prunus avium | Otu00201 | Pseudogymnoascus_roseus | Ascomycota | 1.494 | -1.549 |
| Prunus avium | Otu00044 | Incertae_sedis_unclassified | Basidiomycota | 2.129 | -1.755 |
| Prunus avium | Otu00249 | Schizothecium_glutinans | Ascomycota | 1.292 | -2.027 |
| Prunus avium | Otu00273 | Mortierella_minutissima | Zygomycota | 1.286 | -2.038 |
| Prunus avium | Otu00242 | Ilyonectria_mors-panacis | Ascomycota | 1.082 | -2.089 |
| Prunus avium | Otu00267 | Cryptococcus_carnescens | Basidiomycota | 1.066 | -2.206 |
| Prunus avium | Otu00311 | Cryptococcus_victoriae | Basidiomycota | 1.079 | -2.476 |
| Prunus avium | Otu00344 | Staphylotrichum_boninense | Ascomycota | 0.996 | -2.556 |
| Prunus avium | Otu00402 | Pleosporales_unclassified | Ascomycota | 0.516 | -2.563 |
| Prunus avium | Otu00110 | Pyrenochaetopsis_leptospora | Ascomycota | 1.745 | -2.685 |
| Prunus avium | Otu00004 | Helotiales_unclassified | Ascomycota | 3.235 | -2.805 |
| Prunus avium | Otu00826 | Tuber_sp | Ascomycota | 0.539 | -2.813 |
| Prunus avium | Otu00240 | Septoglomus_viscosum | Glomeromycota | 1.102 | -2.966 |
| Prunus avium | Otu00213 | Alternaria_tenuissima | Ascomycota | 1.484 | -2.994 |
| Prunus avium | Otu00518 | Phaeosphaeriaceae_unclassified | Ascomycota | 0.185 | -3.006 |
| Prunus avium | Otu00674 | Ramicandelaber_sp | Zygomycota | 0.396 | -3.009 |
| Prunus avium | Otu00204 | Montagnulaceae_unclassified | Ascomycota | 0.754 | -3.055 |
| Prunus avium | Otu00302 | Sordariomycetes_unclassified | Ascomycota | 1.114 | -3.278 |
| Prunus avium | Otu00505 | Malassezia_restricta | Basidiomycota | 0.397 | -3.396 |
| Prunus avium | Otu00578 | Coniochaeta_sp | Ascomycota | 0.100 | -3.479 |
| Prunus avium | Otu00520 | Acremonium_psammosporum | Ascomycota | 0.360 | -3.545 |
| Prunus avium | Otu00153 | Ilyonectria_robusta | Ascomycota | 1.115 | -3.628 |
| Prunus avium | Otu00376 | Ramularia_unclassified | Ascomycota | 0.399 | -3.640 |
| Prunus avium | Otu00219 | Hypocreales_unclassified | Ascomycota | 0.821 | -3.783 |
| Prunus avium | Otu00561 | Sphaerulina_pseudovirgaureae | Ascomycota | 0.284 | -3.817 |
| Prunus avium | Otu00122 | Myrmecridium_unclassified | Ascomycota | 1.369 | -3.849 |
| Prunus avium | Otu00689 | Acremonium_stromaticum | Ascomycota | 0.285 | -3.885 |
| Prunus avium | Otu00649 | Paxillus_unclassified | Basidiomycota | 0.081 | -3.891 |
| Prunus avium | Otu00147 | Septoglomus_viscosum | Glomeromycota | 1.291 | -3.951 |
| Prunus avium | Otu00312 | Pezizaceae_sp | Ascomycota | 0.187 | -3.973 |
| Prunus avium | Otu00423 | Lecanicillium_primulinum | Ascomycota | 0.187 | -4.037 |
| Prunus avium | Otu00282 | Septoglomus_viscosum | Glomeromycota | 0.921 | -4.102 |
| Prunus avium | Otu00305 | Monographella_cucumerina | Ascomycota | 0.796 | -4.135 |
| Prunus avium | Otu00276 | Talaromyces_unclassified | Ascomycota | 0.499 | -4.233 |
| Prunus avium | Otu00335 | Pleosporales_unclassified | Ascomycota | 0.359 | -4.359 |
| Prunus avium | Otu00438 | Phaeosphaeriaceae_unclassified | Ascomycota | 0.325 | -4.448 |
| Prunus avium | Otu00118 | Glarea_unclassified | Ascomycota | 1.196 | -4.497 |
| Prunus avium | Otu00393 | Glomeraceae_sp | Glomeromycota | 0.281 | -4.497 |
| Prunus avium | Otu00478 | Pleosporales_unclassified | Ascomycota | 0.485 | -4.541 |
| Prunus avium | Otu00058 | Calyptella_sp | Basidiomycota | 1.162 | -4.550 |
| Prunus avium | Otu00424 | Cryptococcus_heimaeyensis | Basidiomycota | 0.787 | -4.567 |
| Prunus avium | Otu00130 | Mycosphaerella_tassiana | Ascomycota | 1.421 | -4.573 |
| Prunus avium | Otu00534 | Fungi_unclassified | Fungi_unclassified | 0.420 | -4.600 |
| Prunus avium | Otu00158 | Cortinarius_helobius | Basidiomycota | 0.487 | -4.666 |
| Prunus avium | Otu00500 | Paecilomyces_dactylethromorphus | Ascomycota | 0.398 | -4.670 |
| Prunus avium | Otu00139 | Glarea_unclassified | Ascomycota | 0.605 | -4.965 |
| Prunus avium | Otu00463 | Monographella_cucumerina | Ascomycota | 0.473 | -5.073 |
| Prunus avium | Otu00206 | Scytalidium_circinatum | Ascomycota | 0.924 | -5.090 |
| Prunus avium | Otu00471 | Acremonium_alternatum | Ascomycota | 0.699 | -5.102 |
| Prunus avium | Otu00445 | Hirsutella_vermicola | Ascomycota | 0.587 | -5.104 |
| Prunus avium | Otu00131 | Periconia_sp | Ascomycota | 1.464 | -5.142 |
| Prunus avium | Otu00128 | Fungi_unclassified | Fungi_unclassified | 0.792 | -5.222 |
| Prunus avium | Otu00045 | Geopora_cervina | Ascomycota | 0.683 | -5.261 |
| Prunus avium | Otu00277 | Helotiales_unclassified | Ascomycota | 0.822 | -5.623 |
| Prunus avium | Otu00215 | Rhizophagus_intraradices | Glomeromycota | 0.742 | -5.627 |
| Prunus avium | Otu00250 | Coprinopsis_unclassified | Basidiomycota | 0.973 | -5.665 |
| Prunus avium | Otu00129 | Lactarius_scoticus | Basidiomycota | 1.063 | -5.750 |
| Prunus avium | Otu00156 | Auricularia_unclassified | Basidiomycota | 0.820 | -6.023 |
| Prunus avium | Otu00207 | Fungi_unclassified | Fungi_unclassified | 1.353 | -6.034 |
| Prunus avium | Otu00096 | Tomentella_sp | Basidiomycota | 1.349 | -6.054 |
| Prunus avium | Otu00013 | Hebeloma_mesophaeum | Basidiomycota | 3.113 | -6.216 |
| Prunus avium | Otu00251 | Agaricomycetes_unclassified | Basidiomycota | 0.975 | -6.402 |
| Prunus avium | Otu00102 | Tomentella_ellisii | Basidiomycota | 1.097 | -6.613 |
| Prunus avium | Otu00024 | Tuber_maculatum | Ascomycota | 1.777 | -6.685 |
| Prunus avium | Otu00679 | Tuber_sp | Ascomycota | 1.241 | -6.757 |
| Prunus avium | Otu00079 | Talaromyces_aculeatus | Ascomycota | 2.083 | -6.891 |
| Prunus avium | Otu00132 | Rhizophagus_sp | Glomeromycota | 1.058 | -6.905 |
| Prunus avium | Otu00192 | Incertae_sedis_unclassified | Ascomycota | 1.100 | -6.937 |
| Prunus avium | Otu00187 | Apodus_deciduus | Ascomycota | 1.100 | -7.086 |
| Prunus avium | Otu00099 | Geopora_cervina | Ascomycota | 1.170 | -7.158 |
| Prunus avium | Otu00026 | Peziza_michelii | Ascomycota | 1.227 | -7.217 |
| Prunus avium | Otu00051 | Ascomycota_unclassified | Ascomycota | 2.498 | -7.471 |
| Prunus avium | Otu00223 | Sebacinales_unclassified | Basidiomycota | 1.478 | -8.339 |
| Prunus avium | Otu00064 | Ascomycota_unclassified | Ascomycota | 1.430 | -8.411 |
| Prunus avium | Otu00083 | Incertae_sedis_unclassified | Basidiomycota | 1.680 | -8.640 |
| Prunus avium | Otu00020 | Oidiodendron_unclassified | Ascomycota | 2.862 | -8.691 |
| Prunus avium | Otu00134 | Alnicola_macrospora | Basidiomycota | 1.178 | -8.762 |
| Prunus avium | Otu00087 | Paxillus_unclassified | Basidiomycota | 1.708 | -9.425 |
| Prunus avium | Otu00032 | Tuber_maculatum | Ascomycota | 1.612 | -9.641 |
| Prunus avium | Otu00232 | Flagelloscypha_minutissima | Basidiomycota | 0.811 | -22.171 |
| **OTUs significantly enriched or depleted in Sorbus aucuparia** | |  |  |  |  |
| Sorbus aucuparia | Otu00159 | Psathyrella_corrugis | Basidiomycota | 1.581 | 11.559 |
| Sorbus aucuparia | Otu00031 | Pleosporales_unclassified | Ascomycota | 2.359 | 1.999 |
| Sorbus aucuparia | Otu00075 | Fusarium_unclassified | Ascomycota | 2.108 | -1.582 |
| Sorbus aucuparia | Otu00105 | Sordariomycetes_unclassified | Ascomycota | 1.952 | -2.023 |
| Sorbus aucuparia | Otu00146 | Mortierella_alpina | Zygomycota | 1.899 | -2.074 |
| Sorbus aucuparia | Otu00057 | Microdochium_bolleyi | Ascomycota | 2.092 | -2.360 |
| Sorbus aucuparia | Otu00103 | Dendryphion_nanum | Ascomycota | 1.414 | -2.427 |
| Sorbus aucuparia | Otu00214 | Fusicolla_unclassified | Ascomycota | 1.447 | -2.486 |
| Sorbus aucuparia | Otu00110 | Pyrenochaetopsis_leptospora | Ascomycota | 1.741 | -2.704 |
| Sorbus aucuparia | Otu00479 | Cylindrocarpon_sp | Ascomycota | 0.574 | -2.783 |
| Sorbus aucuparia | Otu00098 | Clonostachys_rosea | Ascomycota | 1.703 | -2.900 |
| Sorbus aucuparia | Otu00176 | Mortierella_alpina | Zygomycota | 1.674 | -3.073 |
| Sorbus aucuparia | Otu00628 | Mortierella_unclassified | Zygomycota | 0.232 | -3.305 |
| Sorbus aucuparia | Otu00273 | Mortierella_minutissima | Zygomycota | 1.286 | -3.330 |
| Sorbus aucuparia | Otu00418 | Mortierella_antarctica | Zygomycota | 0.714 | -3.332 |
| Sorbus aucuparia | Otu00227 | Gibberella_zeae | Ascomycota | 1.389 | -3.462 |
| Sorbus aucuparia | Otu00197 | Schizothecium_carpinicola | Ascomycota | 1.132 | -3.929 |
| Sorbus aucuparia | Otu00674 | Ramicandelaber_sp | Zygomycota | 0.396 | -4.164 |
| Sorbus aucuparia | Otu00285 | Nectria_ramulariae | Ascomycota | 1.083 | -4.375 |
| Sorbus aucuparia | Otu00518 | Phaeosphaeriaceae_unclassified | Ascomycota | 0.172 | -4.469 |
| Sorbus aucuparia | Otu00040 | Flagelloscypha_minutissima | Basidiomycota | 2.241 | -4.475 |
| Sorbus aucuparia | Otu00051 | Ascomycota_unclassified | Ascomycota | 2.496 | -4.532 |
| Sorbus aucuparia | Otu00216 | Ceratobasidium_unclassified | Basidiomycota | 1.382 | -4.794 |
| Sorbus aucuparia | Otu00402 | Pleosporales_unclassified | Ascomycota | 0.513 | -4.930 |
| Sorbus aucuparia | Otu00049 | Rhizophagus_irregularis | Glomeromycota | 1.988 | -4.955 |
| Sorbus aucuparia | Otu00561 | Sphaerulina_pseudovirgaureae | Ascomycota | 0.284 | -4.956 |
| Sorbus aucuparia | Otu00256 | Mortierella_hyalina | Zygomycota | 1.306 | -5.065 |
| Sorbus aucuparia | Otu00450 | Chrysosporium_pseudomerdarium | Ascomycota | 0.647 | -5.272 |
| Sorbus aucuparia | Otu00091 | Ascomycota_sp | Ascomycota | 1.640 | -5.276 |
| Sorbus aucuparia | Otu00259 | Septoglomus_viscosum | Glomeromycota | 0.712 | -5.318 |
| Sorbus aucuparia | Otu00396 | Trichoderma_unclassified | Ascomycota | 0.709 | -5.374 |
| Sorbus aucuparia | Otu00369 | Ascobolaceae_sp | Ascomycota | 0.444 | -5.408 |
| Sorbus aucuparia | Otu00153 | Ilyonectria_robusta | Ascomycota | 1.115 | -5.491 |
| Sorbus aucuparia | Otu00383 | Diversisporaceae_sp | Glomeromycota | 0.485 | -5.553 |
| Sorbus aucuparia | Otu00430 | Darksidea_unclassified | Ascomycota | 0.497 | -5.619 |
| Sorbus aucuparia | Otu00240 | Septoglomus_viscosum | Glomeromycota | 0.992 | -5.648 |
| Sorbus aucuparia | Otu00170 | Ceratobasidiaceae_sp | Basidiomycota | 1.619 | -5.795 |
| Sorbus aucuparia | Otu00019 | Hebeloma_sp | Basidiomycota | 2.592 | -5.892 |
| Sorbus aucuparia | Otu00257 | Septoglomus_constrictum | Glomeromycota | 1.029 | -5.922 |
| Sorbus aucuparia | Otu00204 | Montagnulaceae_unclassified | Ascomycota | 0.776 | -6.113 |
| Sorbus aucuparia | Otu00221 | Geopyxis_unclassified | Ascomycota | 0.984 | -6.161 |
| Sorbus aucuparia | Otu00471 | Acremonium_alternatum | Ascomycota | 0.692 | -6.235 |
| Sorbus aucuparia | Otu00382 | Ophiosphaerella_sp | Ascomycota | 0.816 | -6.249 |
| Sorbus aucuparia | Otu00289 | Chaetomium_unclassified | Ascomycota | 0.959 | -6.337 |
| Sorbus aucuparia | Otu00408 | Acremonium_persicinum | Ascomycota | 0.753 | -6.369 |
| Sorbus aucuparia | Otu00378 | Fusarium_poae | Ascomycota | 0.755 | -6.427 |
| Sorbus aucuparia | Otu00302 | Sordariomycetes_unclassified | Ascomycota | 1.147 | -6.825 |
| Sorbus aucuparia | Otu00251 | Agaricomycetes_unclassified | Basidiomycota | 0.980 | -7.187 |
| Sorbus aucuparia | Otu00117 | Psathyrella_panaeoloides | Basidiomycota | 1.515 | -7.207 |
| Sorbus aucuparia | Otu00083 | Incertae_sedis_unclassified | Basidiomycota | 1.702 | -7.678 |
| Sorbus aucuparia | Otu00099 | Geopora_cervina | Ascomycota | 1.170 | -7.804 |
| Sorbus aucuparia | Otu00679 | Tuber_sp | Ascomycota | 1.241 | -7.835 |
| Sorbus aucuparia | Otu00134 | Alnicola_macrospora | Basidiomycota | 1.183 | -7.856 |
| Sorbus aucuparia | Otu00128 | Fungi_unclassified | Fungi_unclassified | 1.079 | -7.953 |
| Sorbus aucuparia | Otu00026 | Peziza_michelii | Ascomycota | 1.227 | -7.995 |
| Sorbus aucuparia | Otu00058 | Calyptella_sp | Basidiomycota | 1.182 | -8.297 |
| Sorbus aucuparia | Otu00038 | Hebeloma_sp | Basidiomycota | 1.631 | -8.376 |
| Sorbus aucuparia | Otu00190 | Septoglomus_unclassified | Glomeromycota | 1.421 | -8.647 |
| Sorbus aucuparia | Otu00182 | Septoglomus_constrictum | Glomeromycota | 1.499 | -8.905 |
| Sorbus aucuparia | Otu00064 | Ascomycota_unclassified | Ascomycota | 1.430 | -8.930 |
| Sorbus aucuparia | Otu00032 | Tuber_maculatum | Ascomycota | 1.613 | -9.731 |
| Sorbus aucuparia | Otu00084 | Stephanosporaceae_sp | Basidiomycota | 0.953 | -21.035 |
| Sorbus aucuparia | Otu00113 | Entoloma_lanicum | Basidiomycota | 1.001 | -21.794 |
| Sorbus aucuparia | Otu00168 | Glomeraceae_unclassified | Glomeromycota | 1.068 | -22.001 |
| Sorbus aucuparia | Otu00102 | Tomentella_ellisii | Basidiomycota | 1.097 | -22.059 |
| Sorbus aucuparia | Otu00074 | Fungi_unclassified | Fungi_unclassified | 1.387 | -22.178 |
| Sorbus aucuparia | Otu00162 | Fungi_sp | unclassified_Fungi | 1.165 | -22.322 |
| Sorbus aucuparia | Otu00053 | Fungi_unclassified | Fungi_unclassified | 1.417 | -22.507 |
| Sorbus aucuparia | Otu00096 | Tomentella_sp | Basidiomycota | 1.352 | -22.877 |
| **OTUs significantly enriched or depleted in Betula pendula** | |  |  |  |  |
| Betula pendula | Otu00129 | Lactarius_scoticus | Basidiomycota | 2.182 | 7.553 |
| Betula pendula | Otu00133 | Bartalinia_robillardoides | Ascomycota | 1.614 | 4.474 |
| Betula pendula | Otu00019 | Hebeloma_sp | Basidiomycota | 3.365 | 3.116 |
| Betula pendula | Otu00013 | Hebeloma_mesophaeum | Basidiomycota | 3.296 | 2.632 |
| Betula pendula | Otu00157 | Exophiala_opportunistica | Ascomycota | 1.650 | 2.170 |
| Betula pendula | Otu00010 | Exophiala_unclassified | Ascomycota | 3.213 | 1.026 |
| Betula pendula | Otu00057 | Microdochium_bolleyi | Ascomycota | 2.092 | -1.939 |
| Betula pendula | Otu00004 | Helotiales_unclassified | Ascomycota | 3.257 | -1.942 |
| Betula pendula | Otu00078 | Ascomycota_unclassified | Ascomycota | 2.231 | -2.435 |
| Betula pendula | Otu00012 | Rhizophagus_irregularis | Glomeromycota | 3.115 | -2.668 |
| Betula pendula | Otu00056 | Xylariales_sp | Ascomycota | 2.128 | -3.050 |
| Betula pendula | Otu00344 | Staphylotrichum_boninense | Ascomycota | 1.007 | -3.290 |
| Betula pendula | Otu00015 | Rhizophagus_irregularis | Glomeromycota | 3.026 | -3.354 |
| Betula pendula | Otu00483 | Mortierella_unclassified | Zygomycota | 0.550 | -3.446 |
| Betula pendula | Otu00014 | Rhizophagus_irregularis | Glomeromycota | 3.092 | -3.482 |
| Betula pendula | Otu00081 | Ascomycota_unclassified | Ascomycota | 2.066 | -3.564 |
| Betula pendula | Otu00011 | Rhizophagus_irregularis | Glomeromycota | 3.116 | -3.706 |
| Betula pendula | Otu00358 | Pulvinula_sp | Ascomycota | 0.512 | -3.832 |
| Betula pendula | Otu00424 | Cryptococcus_heimaeyensis | Basidiomycota | 0.787 | -3.908 |
| Betula pendula | Otu00325 | Lasiosphaeriaceae_unclassified | Ascomycota | 0.846 | -3.910 |
| Betula pendula | Otu00386 | Basidiomycota_unclassified | Basidiomycota | 0.565 | -3.985 |
| Betula pendula | Otu00021 | Rhizophagus_irregularis | Glomeromycota | 2.875 | -4.038 |
| Betula pendula | Otu00163 | Pyrenochaeta_inflorescentiae | Ascomycota | 1.420 | -4.141 |
| Betula pendula | Otu00271 | Phaeoacremonium_hungaricum | Ascomycota | 0.874 | -4.161 |
| Betula pendula | Otu00379 | Gibellulopsis_nigrescens | Ascomycota | 0.686 | -4.279 |
| Betula pendula | Otu00345 | Rhizophagus_irregularis | Glomeromycota | 0.784 | -4.320 |
| Betula pendula | Otu00027 | Agaricales_unclassified | Basidiomycota | 2.453 | -4.353 |
| Betula pendula | Otu00259 | Septoglomus_viscosum | Glomeromycota | 0.712 | -4.457 |
| Betula pendula | Otu00408 | Acremonium_persicinum | Ascomycota | 0.753 | -4.495 |
| Betula pendula | Otu00378 | Fusarium_poae | Ascomycota | 0.755 | -4.553 |
| Betula pendula | Otu00204 | Montagnulaceae_unclassified | Ascomycota | 0.761 | -4.602 |
| Betula pendula | Otu00193 | Slopeiomyces_cylindrosporus | Ascomycota | 1.689 | -4.748 |
| Betula pendula | Otu00017 | Rhizophagus_irregularis | Glomeromycota | 3.066 | -4.752 |
| Betula pendula | Otu00282 | Septoglomus_viscosum | Glomeromycota | 0.823 | -4.781 |
| Betula pendula | Otu00190 | Septoglomus_unclassified | Glomeromycota | 1.419 | -4.826 |
| Betula pendula | Otu00255 | Herpotrichiellaceae_unclassified | Ascomycota | 0.848 | -4.887 |
| Betula pendula | Otu00256 | Mortierella_hyalina | Zygomycota | 1.306 | -5.087 |
| Betula pendula | Otu00104 | Pezizaceae_sp | Ascomycota | 0.963 | -5.257 |
| Betula pendula | Otu00202 | Diversisporaceae_sp | Glomeromycota | 1.318 | -5.260 |
| Betula pendula | Otu00122 | Myrmecridium_unclassified | Ascomycota | 1.408 | -5.265 |
| Betula pendula | Otu00112 | Sebacinales_sp | Basidiomycota | 1.464 | -5.288 |
| Betula pendula | Otu00221 | Geopyxis_unclassified | Ascomycota | 0.991 | -5.328 |
| Betula pendula | Otu00080 | Glomeraceae_unclassified | Glomeromycota | 2.068 | -5.333 |
| Betula pendula | Otu00240 | Septoglomus_viscosum | Glomeromycota | 0.992 | -5.357 |
| Betula pendula | Otu00061 | Pulvinula_sp | Ascomycota | 1.927 | -5.371 |
| Betula pendula | Otu00211 | Glomus_sp | Glomeromycota | 1.030 | -5.476 |
| Betula pendula | Otu00257 | Septoglomus_constrictum | Glomeromycota | 1.036 | -5.502 |
| Betula pendula | Otu00283 | Glomeraceae_unclassified | Glomeromycota | 1.049 | -5.547 |
| Betula pendula | Otu00295 | Glomeraceae_sp | Glomeromycota | 1.063 | -5.568 |
| Betula pendula | Otu00049 | Rhizophagus_irregularis | Glomeromycota | 1.986 | -5.615 |
| Betula pendula | Otu00275 | Glomeraceae_sp | Glomeromycota | 1.095 | -5.701 |
| Betula pendula | Otu00230 | Monographella_cucumerina | Ascomycota | 1.227 | -5.756 |
| Betula pendula | Otu00171 | Glomeraceae_sp | Glomeromycota | 1.115 | -5.761 |
| Betula pendula | Otu00037 | Glomus_sp | Glomeromycota | 2.489 | -5.800 |
| Betula pendula | Otu00212 | Claroideoglomeraceae_unclassified | Glomeromycota | 1.157 | -5.905 |
| Betula pendula | Otu00150 | Rhizophagus_irregularis | Glomeromycota | 1.167 | -5.933 |
| Betula pendula | Otu00147 | Septoglomus_viscosum | Glomeromycota | 1.293 | -5.984 |
| Betula pendula | Otu00047 | Glomus_sp | Glomeromycota | 1.540 | -6.083 |
| Betula pendula | Otu00174 | Claroideoglomus_sp | Glomeromycota | 1.222 | -6.108 |
| Betula pendula | Otu00216 | Ceratobasidium_unclassified | Basidiomycota | 1.372 | -6.241 |
| Betula pendula | Otu00218 | Glomus_sp | Glomeromycota | 1.266 | -6.262 |
| Betula pendula | Otu00121 | Rhizophagus_irregularis | Glomeromycota | 1.420 | -6.771 |
| Betula pendula | Otu00118 | Glarea_unclassified | Ascomycota | 1.268 | -6.793 |
| Betula pendula | Otu00189 | Glomus_sp | Glomeromycota | 1.437 | -6.825 |
| Betula pendula | Otu00182 | Septoglomus_constrictum | Glomeromycota | 1.499 | -7.032 |
| Betula pendula | Otu00109 | Claroideoglomus_sp | Glomeromycota | 1.617 | -7.064 |
| Betula pendula | Otu00086 | Subulicystidium_sp | Basidiomycota | 1.520 | -7.189 |
| Betula pendula | Otu00050 | Rhizophagus_irregularis | Glomeromycota | 2.233 | -7.209 |
| Betula pendula | Otu00053 | Fungi_unclassified | Fungi_unclassified | 1.432 | -7.655 |
| Betula pendula | Otu00170 | Ceratobasidiaceae_sp | Basidiomycota | 1.640 | -7.828 |
| Betula pendula | Otu00090 | Agaricales_unclassified | Basidiomycota | 1.720 | -7.937 |
| Betula pendula | Otu00063 | Rhizophagus_unclassified | Glomeromycota | 1.579 | -8.061 |
| Betula pendula | Otu00071 | Auriculariales_unclassified | Basidiomycota | 1.664 | -8.369 |
| Betula pendula | Otu00024 | Tuber_maculatum | Ascomycota | 1.777 | -8.762 |
| Betula pendula | Otu00065 | Glomeraceae_unclassified | Glomeromycota | 2.168 | -9.181 |
| Betula pendula | Otu00102 | Tomentella_ellisii | Basidiomycota | 1.074 | -19.692 |
| Betula pendula | Otu00084 | Stephanosporaceae_sp | Basidiomycota | 1.001 | -20.306 |
| Betula pendula | Otu00113 | Entoloma_lanicum | Basidiomycota | 1.001 | -20.321 |
| Betula pendula | Otu00168 | Glomeraceae_unclassified | Glomeromycota | 1.085 | -20.588 |
| Betula pendula | Otu00162 | Fungi_sp | unclassified_Fungi | 1.167 | -20.774 |
| Betula pendula | Otu00067 | Stephanosporaceae_sp | Basidiomycota | 1.332 | -21.051 |
| Betula pendula | Otu00127 | Thielaviopsis_basicola | Ascomycota | 1.283 | -21.220 |
| Betula pendula | Otu00074 | Fungi_unclassified | Fungi_unclassified | 1.388 | -21.459 |
| Betula pendula | Otu00083 | Incertae_sedis_unclassified | Basidiomycota | 1.650 | -22.009 |
| Betula pendula | Otu00092 | Glomus_sp | Glomeromycota | 1.834 | -22.990 |
| Betula pendula | Otu00085 | Glomeraceae_sp | Glomeromycota | 1.971 | -23.239 |
| **OTUs significantly enriched or depleted in Carpinus betulus** | |  |  |  |  |
| Carpinus betulus | Otu00134 | Alnicola_macrospora | Basidiomycota | 2.114 | 10.021 |
| Carpinus betulus | Otu00319 | Incertae_sedis_unclassified | Ascomycota | 1.064 | 7.958 |
| Carpinus betulus | Otu00083 | Incertae_sedis_unclassified | Basidiomycota | 2.317 | 7.548 |
| Carpinus betulus | Otu00188 | Pezizaceae_sp | Ascomycota | 0.707 | 7.496 |
| Carpinus betulus | Otu00102 | Tomentella_ellisii | Basidiomycota | 1.500 | 6.867 |
| Carpinus betulus | Otu00026 | Peziza_michelii | Ascomycota | 1.030 | 6.744 |
| Carpinus betulus | Otu00404 | Incertae_sedis_unclassified | Basidiomycota | 0.953 | 6.094 |
| Carpinus betulus | Otu00153 | Ilyonectria_robusta | Ascomycota | 1.597 | 5.416 |
| Carpinus betulus | Otu00312 | Pezizaceae_sp | Ascomycota | 0.602 | 4.799 |
| Carpinus betulus | Otu00058 | Calyptella_sp | Basidiomycota | 1.409 | 4.089 |
| Carpinus betulus | Otu00192 | Incertae_sedis_unclassified | Ascomycota | 1.218 | 3.690 |
| Carpinus betulus | Otu00249 | Schizothecium_glutinans | Ascomycota | 1.442 | 3.007 |
| Carpinus betulus | Otu00561 | Sphaerulina_pseudovirgaureae | Ascomycota | 0.284 | 2.883 |
| Carpinus betulus | Otu00052 | Strumella_sp | Ascomycota | 2.340 | 1.187 |
| Carpinus betulus | Otu00009 | Tetracladium_maxilliforme | Ascomycota | 3.174 | 0.788 |
| Carpinus betulus | Otu00115 | Acremonium_nepalense | Ascomycota | 1.766 | -1.135 |
| Carpinus betulus | Otu00039 | Leotiomycetes_unclassified | Ascomycota | 2.420 | -1.167 |
| Carpinus betulus | Otu00098 | Clonostachys_rosea | Ascomycota | 1.680 | -1.411 |
| Carpinus betulus | Otu00014 | Rhizophagus_irregularis | Glomeromycota | 3.092 | -1.624 |
| Carpinus betulus | Otu00021 | Rhizophagus_irregularis | Glomeromycota | 2.875 | -1.739 |
| Carpinus betulus | Otu00094 | Paraphoma_unclassified | Ascomycota | 1.592 | -1.796 |
| Carpinus betulus | Otu00345 | Rhizophagus_irregularis | Glomeromycota | 0.879 | -1.845 |
| Carpinus betulus | Otu00169 | Sordariomycetes_unclassified | Ascomycota | 1.648 | -1.916 |
| Carpinus betulus | Otu00619 | Rhizophagus_irregularis | Glomeromycota | 0.143 | -1.972 |
| Carpinus betulus | Otu00321 | Schizothecium_inaequale | Ascomycota | 0.912 | -1.979 |
| Carpinus betulus | Otu00287 | Sordariales_unclassified | Ascomycota | 1.051 | -2.091 |
| Carpinus betulus | Otu00015 | Rhizophagus_irregularis | Glomeromycota | 3.026 | -2.163 |
| Carpinus betulus | Otu00011 | Rhizophagus_irregularis | Glomeromycota | 3.116 | -2.180 |
| Carpinus betulus | Otu00012 | Rhizophagus_irregularis | Glomeromycota | 3.115 | -2.244 |
| Carpinus betulus | Otu00552 | Fungi_unclassified | Fungi_unclassified | 0.271 | -2.261 |
| Carpinus betulus | Otu00050 | Rhizophagus_irregularis | Glomeromycota | 2.169 | -2.598 |
| Carpinus betulus | Otu00049 | Rhizophagus_irregularis | Glomeromycota | 1.983 | -2.600 |
| Carpinus betulus | Otu00038 | Hebeloma_sp | Basidiomycota | 1.593 | -2.644 |
| Carpinus betulus | Otu00498 | Pleosporales_unclassified | Ascomycota | 0.428 | -2.752 |
| Carpinus betulus | Otu00396 | Trichoderma_unclassified | Ascomycota | 0.733 | -2.799 |
| Carpinus betulus | Otu00402 | Pleosporales_unclassified | Ascomycota | 0.499 | -2.822 |
| Carpinus betulus | Otu00040 | Flagelloscypha_minutissima | Basidiomycota | 2.364 | -2.880 |
| Carpinus betulus | Otu00189 | Glomus_sp | Glomeromycota | 1.399 | -2.882 |
| Carpinus betulus | Otu00565 | Rhizophagus_irregularis | Glomeromycota | 0.248 | -2.896 |
| Carpinus betulus | Otu00358 | Pulvinula_sp | Ascomycota | 0.512 | -2.970 |
| Carpinus betulus | Otu00133 | Bartalinia_robillardoides | Ascomycota | 1.114 | -3.057 |
| Carpinus betulus | Otu00403 | Aureobasidium_pullulans | Ascomycota | 0.436 | -3.125 |
| Carpinus betulus | Otu00091 | Ascomycota_sp | Ascomycota | 1.761 | -3.264 |
| Carpinus betulus | Otu00366 | Glomeraceae_sp | Glomeromycota | 0.429 | -3.276 |
| Carpinus betulus | Otu00197 | Schizothecium_carpinicola | Ascomycota | 1.099 | -3.339 |
| Carpinus betulus | Otu00078 | Ascomycota_unclassified | Ascomycota | 2.224 | -3.351 |
| Carpinus betulus | Otu00034 | Hebeloma_sp | Basidiomycota | 0.410 | -3.370 |
| Carpinus betulus | Otu00086 | Subulicystidium_sp | Basidiomycota | 1.580 | -3.406 |
| Carpinus betulus | Otu00131 | Periconia_sp | Ascomycota | 1.318 | -3.468 |
| Carpinus betulus | Otu00459 | Metarhizium_marquandii | Ascomycota | 0.397 | -3.526 |
| Carpinus betulus | Otu00190 | Septoglomus_unclassified | Glomeromycota | 1.421 | -3.531 |
| Carpinus betulus | Otu00081 | Ascomycota_unclassified | Ascomycota | 2.066 | -3.585 |
| Carpinus betulus | Otu00161 | Agaricomycetes_unclassified | Basidiomycota | 1.055 | -3.594 |
| Carpinus betulus | Otu00315 | Glomus_sp | Glomeromycota | 0.513 | -3.878 |
| Carpinus betulus | Otu00355 | Claroideoglomus_sp | Glomeromycota | 0.556 | -3.882 |
| Carpinus betulus | Otu00383 | Diversisporaceae_sp | Glomeromycota | 0.562 | -3.995 |
| Carpinus betulus | Otu00367 | Glomus_sp | Glomeromycota | 0.555 | -4.008 |
| Carpinus betulus | Otu00264 | Fungi_unclassified | Fungi_unclassified | 0.665 | -4.024 |
| Carpinus betulus | Otu00017 | Rhizophagus_irregularis | Glomeromycota | 3.068 | -4.030 |
| Carpinus betulus | Otu00257 | Septoglomus_constrictum | Glomeromycota | 1.036 | -4.103 |
| Carpinus betulus | Otu00149 | Glomus_compressum | Glomeromycota | 0.667 | -4.184 |
| Carpinus betulus | Otu00390 | Paraglomerales_sp | Glomeromycota | 0.624 | -4.205 |
| Carpinus betulus | Otu00211 | Glomus_sp | Glomeromycota | 1.100 | -4.356 |
| Carpinus betulus | Otu00240 | Septoglomus_viscosum | Glomeromycota | 1.102 | -4.363 |
| Carpinus betulus | Otu00353 | Glomus_sp | Glomeromycota | 0.683 | -4.403 |
| Carpinus betulus | Otu00182 | Septoglomus_constrictum | Glomeromycota | 1.499 | -4.439 |
| Carpinus betulus | Otu00230 | Monographella_cucumerina | Ascomycota | 1.187 | -4.607 |
| Carpinus betulus | Otu00282 | Septoglomus_viscosum | Glomeromycota | 0.925 | -4.639 |
| Carpinus betulus | Otu00147 | Septoglomus_viscosum | Glomeromycota | 1.293 | -4.692 |
| Carpinus betulus | Otu00053 | Fungi_unclassified | Fungi_unclassified | 1.654 | -4.772 |
| Carpinus betulus | Otu00220 | Lasiosphaeriaceae_unclassified | Ascomycota | 1.105 | -4.807 |
| Carpinus betulus | Otu00090 | Agaricales_unclassified | Basidiomycota | 1.862 | -4.849 |
| Carpinus betulus | Otu00206 | Scytalidium_circinatum | Ascomycota | 0.911 | -4.892 |
| Carpinus betulus | Otu00129 | Lactarius_scoticus | Basidiomycota | 1.007 | -4.910 |
| Carpinus betulus | Otu00099 | Geopora_cervina | Ascomycota | 1.160 | -4.929 |
| Carpinus betulus | Otu00071 | Auriculariales_unclassified | Basidiomycota | 1.395 | -4.934 |
| Carpinus betulus | Otu00679 | Tuber_sp | Ascomycota | 1.241 | -4.971 |
| Carpinus betulus | Otu00187 | Apodus_deciduus | Ascomycota | 1.089 | -5.158 |
| Carpinus betulus | Otu00047 | Glomus_sp | Glomeromycota | 1.859 | -5.269 |
| Carpinus betulus | Otu00275 | Glomeraceae_sp | Glomeromycota | 1.095 | -5.417 |
| Carpinus betulus | Otu00127 | Thielaviopsis_basicola | Ascomycota | 1.708 | -5.470 |
| Carpinus betulus | Otu00132 | Rhizophagus_sp | Glomeromycota | 1.058 | -5.571 |
| Carpinus betulus | Otu00283 | Glomeraceae_unclassified | Glomeromycota | 1.038 | -5.578 |
| Carpinus betulus | Otu00065 | Glomeraceae_unclassified | Glomeromycota | 2.168 | -5.640 |
| Carpinus betulus | Otu00024 | Tuber_maculatum | Ascomycota | 1.772 | -5.669 |
| Carpinus betulus | Otu00085 | Glomeraceae_sp | Glomeromycota | 1.966 | -5.716 |
| Carpinus betulus | Otu00096 | Tomentella_sp | Basidiomycota | 1.297 | -5.956 |
| Carpinus betulus | Otu00212 | Claroideoglomeraceae_unclassified | Glomeromycota | 1.157 | -5.975 |
| Carpinus betulus | Otu00150 | Rhizophagus_irregularis | Glomeromycota | 1.167 | -5.998 |
| Carpinus betulus | Otu00121 | Rhizophagus_irregularis | Glomeromycota | 1.406 | -6.072 |
| Carpinus betulus | Otu00202 | Diversisporaceae_sp | Glomeromycota | 1.315 | -6.138 |
| Carpinus betulus | Otu00037 | Glomus_sp | Glomeromycota | 2.487 | -6.177 |
| Carpinus betulus | Otu00027 | Agaricales_unclassified | Basidiomycota | 2.453 | -6.316 |
| Carpinus betulus | Otu00218 | Glomus_sp | Glomeromycota | 1.266 | -6.329 |
| Carpinus betulus | Otu00061 | Pulvinula_sp | Ascomycota | 1.914 | -6.401 |
| Carpinus betulus | Otu00174 | Claroideoglomus_sp | Glomeromycota | 1.297 | -6.427 |
| Carpinus betulus | Otu00084 | Stephanosporaceae_sp | Basidiomycota | 1.205 | -6.552 |
| Carpinus betulus | Otu00109 | Claroideoglomus_sp | Glomeromycota | 1.613 | -6.570 |
| Carpinus betulus | Otu00216 | Ceratobasidium_unclassified | Basidiomycota | 1.505 | -6.657 |
| Carpinus betulus | Otu00032 | Tuber_maculatum | Ascomycota | 1.613 | -6.848 |
| Carpinus betulus | Otu00080 | Glomeraceae_unclassified | Glomeromycota | 2.064 | -7.378 |
| Carpinus betulus | Otu00168 | Glomeraceae_unclassified | Glomeromycota | 1.085 | -7.402 |
| Carpinus betulus | Otu00184 | Agaricomycetes_unclassified | Basidiomycota | 1.129 | -7.794 |
| Carpinus betulus | Otu00067 | Stephanosporaceae_sp | Basidiomycota | 1.332 | -8.069 |
| Carpinus betulus | Otu00063 | Rhizophagus_unclassified | Glomeromycota | 1.713 | -8.135 |
| Carpinus betulus | Otu00092 | Glomus_sp | Glomeromycota | 1.829 | -8.547 |
| Carpinus betulus | Otu00171 | Glomeraceae_sp | Glomeromycota | 1.115 | -9.045 |
| Carpinus betulus | Otu00128 | Fungi_unclassified | Fungi_unclassified | 1.067 | -20.342 |
| Carpinus betulus | Otu00183 | Glomus_sp | Glomeromycota | 0.989 | -21.808 |
| Carpinus betulus | Otu00113 | Entoloma_lanicum | Basidiomycota | 1.001 | -21.958 |
| **OTUs significantly enriched or depleted in Fagus sylvatica** | |  |  |  |  |
| Fagus sylvatica | Otu00429 | Incertae_sedis_unclassified | Basidiomycota | 1.027 | 8.449 |
| Fagus sylvatica | Otu00679 | Tuber_sp | Ascomycota | 1.241 | 8.263 |
| Fagus sylvatica | Otu01609 | Fungi_unclassified | Fungi_unclassified | 0.508 | 7.003 |
| Fagus sylvatica | Otu00032 | Tuber_maculatum | Ascomycota | 1.399 | 6.171 |
| Fagus sylvatica | Otu00165 | Melanogaster_variegatus | Basidiomycota | 0.422 | 5.965 |
| Fagus sylvatica | Otu00415 | Trichopezizella_sp | Ascomycota | 0.548 | 5.418 |
| Fagus sylvatica | Otu00955 | Ilyonectria_macrodidyma | Ascomycota | 0.390 | 5.049 |
| Fagus sylvatica | Otu00826 | Tuber_sp | Ascomycota | 0.539 | 3.547 |
| Fagus sylvatica | Otu00169 | Sordariomycetes_unclassified | Ascomycota | 1.904 | 3.337 |
| Fagus sylvatica | Otu01317 | Tuber_sp | Ascomycota | -0.041 | 3.289 |
| Fagus sylvatica | Otu00966 | Tuber_sp | Ascomycota | 0.246 | 3.131 |
| Fagus sylvatica | Otu00207 | Fungi_unclassified | Fungi_unclassified | 1.820 | 3.031 |
| Fagus sylvatica | Otu00674 | Ramicandelaber_sp | Zygomycota | 0.234 | 2.553 |
| Fagus sylvatica | Otu00078 | Ascomycota_unclassified | Ascomycota | 2.170 | 2.399 |
| Fagus sylvatica | Otu00418 | Mortierella_antarctica | Zygomycota | 0.675 | 2.145 |
| Fagus sylvatica | Otu00273 | Mortierella_minutissima | Zygomycota | 1.286 | 1.999 |
| Fagus sylvatica | Otu00278 | Cryptococcus_aerius | Basidiomycota | 1.194 | 1.375 |
| Fagus sylvatica | Otu00002 | Ilyonectria_macrodidyma | Ascomycota | 3.561 | 1.052 |
| Fagus sylvatica | Otu00009 | Tetracladium_maxilliforme | Ascomycota | 3.138 | -0.653 |
| Fagus sylvatica | Otu00039 | Leotiomycetes_unclassified | Ascomycota | 2.420 | -1.230 |
| Fagus sylvatica | Otu00031 | Pleosporales_unclassified | Ascomycota | 2.359 | -1.305 |
| Fagus sylvatica | Otu00254 | Rhodotorula_ferulica | Basidiomycota | 1.044 | -1.743 |
| Fagus sylvatica | Otu00094 | Paraphoma_unclassified | Ascomycota | 1.592 | -1.859 |
| Fagus sylvatica | Otu00013 | Hebeloma_mesophaeum | Basidiomycota | 3.017 | -1.874 |
| Fagus sylvatica | Otu00025 | Cyphellophora_sp | Ascomycota | 2.510 | -1.980 |
| Fagus sylvatica | Otu00017 | Rhizophagus_irregularis | Glomeromycota | 3.036 | -2.036 |
| Fagus sylvatica | Otu00021 | Rhizophagus_irregularis | Glomeromycota | 2.855 | -2.214 |
| Fagus sylvatica | Otu00037 | Glomus_sp | Glomeromycota | 2.489 | -2.215 |
| Fagus sylvatica | Otu00020 | Oidiodendron_unclassified | Ascomycota | 2.802 | -2.352 |
| Fagus sylvatica | Otu00345 | Rhizophagus_irregularis | Glomeromycota | 0.879 | -2.414 |
| Fagus sylvatica | Otu00190 | Septoglomus_unclassified | Glomeromycota | 1.421 | -2.533 |
| Fagus sylvatica | Otu00050 | Rhizophagus_irregularis | Glomeromycota | 2.233 | -2.594 |
| Fagus sylvatica | Otu00212 | Claroideoglomeraceae_unclassified | Glomeromycota | 1.157 | -2.797 |
| Fagus sylvatica | Otu00112 | Sebacinales_sp | Basidiomycota | 1.476 | -2.797 |
| Fagus sylvatica | Otu00218 | Glomus_sp | Glomeromycota | 1.254 | -2.893 |
| Fagus sylvatica | Otu00027 | Agaricales_unclassified | Basidiomycota | 2.453 | -2.905 |
| Fagus sylvatica | Otu00202 | Diversisporaceae_sp | Glomeromycota | 1.321 | -2.934 |
| Fagus sylvatica | Otu00211 | Glomus_sp | Glomeromycota | 0.974 | -2.984 |
| Fagus sylvatica | Otu00283 | Glomeraceae_unclassified | Glomeromycota | 1.049 | -3.019 |
| Fagus sylvatica | Otu00282 | Septoglomus_viscosum | Glomeromycota | 0.925 | -3.049 |
| Fagus sylvatica | Otu00121 | Rhizophagus_irregularis | Glomeromycota | 1.420 | -3.068 |
| Fagus sylvatica | Otu00182 | Septoglomus_constrictum | Glomeromycota | 1.499 | -3.213 |
| Fagus sylvatica | Otu00150 | Rhizophagus_irregularis | Glomeromycota | 1.167 | -3.283 |
| Fagus sylvatica | Otu00240 | Septoglomus_viscosum | Glomeromycota | 0.971 | -3.386 |
| Fagus sylvatica | Otu00122 | Myrmecridium_unclassified | Ascomycota | 1.400 | -3.567 |
| Fagus sylvatica | Otu00174 | Claroideoglomus_sp | Glomeromycota | 1.297 | -3.664 |
| Fagus sylvatica | Otu00061 | Pulvinula_sp | Ascomycota | 1.927 | -3.935 |
| Fagus sylvatica | Otu00109 | Claroideoglomus_sp | Glomeromycota | 1.617 | -4.056 |
| Fagus sylvatica | Otu00275 | Glomeraceae_sp | Glomeromycota | 1.095 | -4.128 |
| Fagus sylvatica | Otu00197 | Schizothecium_carpinicola | Ascomycota | 1.245 | -4.533 |
| Fagus sylvatica | Otu00189 | Glomus_sp | Glomeromycota | 1.415 | -4.585 |
| Fagus sylvatica | Otu00083 | Incertae_sedis_unclassified | Basidiomycota | 1.646 | -5.317 |
| Fagus sylvatica | Otu00085 | Glomeraceae_sp | Glomeromycota | 1.971 | -5.825 |
| Fagus sylvatica | Otu00024 | Tuber_maculatum | Ascomycota | 1.777 | -5.932 |
| Fagus sylvatica | Otu00090 | Agaricales_unclassified | Basidiomycota | 1.862 | -6.298 |
| Fagus sylvatica | Otu00067 | Stephanosporaceae_sp | Basidiomycota | 1.332 | -7.535 |
| Fagus sylvatica | Otu00084 | Stephanosporaceae_sp | Basidiomycota | 1.205 | -7.539 |
| Fagus sylvatica | Otu00047 | Glomus_sp | Glomeromycota | 1.495 | -7.578 |
| Fagus sylvatica | Otu00232 | Flagelloscypha_minutissima | Basidiomycota | 0.839 | -7.728 |
| Fagus sylvatica | Otu00064 | Ascomycota_unclassified | Ascomycota | 1.430 | -22.295 |
| **OTUs significantly enriched or depleted in Quercus petraea** | |  |  |  |  |
| Quercus petraea | Otu00108 | Rhodoscypha_sp | Ascomycota | 2.103 | 11.306 |
| Quercus petraea | Otu00140 | Phialocephala_fortinii | Ascomycota | 1.621 | 8.631 |
| Quercus petraea | Otu00148 | Scleroderma_areolatum | Basidiomycota | 1.657 | 8.075 |
| Quercus petraea | Otu00019 | Hebeloma_sp | Basidiomycota | 2.851 | 3.978 |
| Quercus petraea | Otu00070 | Mortierella_elongata | Zygomycota | 2.210 | -1.500 |
| Quercus petraea | Otu00012 | Rhizophagus_irregularis | Glomeromycota | 3.115 | -2.042 |
| Quercus petraea | Otu00011 | Rhizophagus_irregularis | Glomeromycota | 3.116 | -2.193 |
| Quercus petraea | Otu00185 | Periconia_sp | Ascomycota | 1.566 | -2.362 |
| Quercus petraea | Otu00591 | Mortierella_rishikesha | Zygomycota | 0.309 | -3.493 |
| Quercus petraea | Otu00252 | Chaetomiaceae_unclassified | Ascomycota | 1.343 | -3.513 |
| Quercus petraea | Otu00021 | Rhizophagus_irregularis | Glomeromycota | 2.875 | -3.678 |
| Quercus petraea | Otu00245 | Gibellulopsis_nigrescens | Ascomycota | 1.258 | -3.750 |
| Quercus petraea | Otu00267 | Cryptococcus_carnescens | Basidiomycota | 1.066 | -3.757 |
| Quercus petraea | Otu00483 | Mortierella_unclassified | Zygomycota | 0.571 | -3.862 |
| Quercus petraea | Otu00015 | Rhizophagus_irregularis | Glomeromycota | 3.026 | -4.172 |
| Quercus petraea | Otu00345 | Rhizophagus_irregularis | Glomeromycota | 0.784 | -4.182 |
| Quercus petraea | Otu00014 | Rhizophagus_irregularis | Glomeromycota | 3.092 | -4.224 |
| Quercus petraea | Otu00379 | Gibellulopsis_nigrescens | Ascomycota | 0.686 | -4.257 |
| Quercus petraea | Otu00402 | Pleosporales_unclassified | Ascomycota | 0.516 | -4.301 |
| Quercus petraea | Otu00358 | Pulvinula_sp | Ascomycota | 0.512 | -4.310 |
| Quercus petraea | Otu00065 | Glomeraceae_unclassified | Glomeromycota | 2.168 | -4.370 |
| Quercus petraea | Otu00450 | Chrysosporium_pseudomerdarium | Ascomycota | 0.649 | -4.644 |
| Quercus petraea | Otu00240 | Septoglomus_viscosum | Glomeromycota | 0.992 | -4.854 |
| Quercus petraea | Otu00373 | Stephanosporaceae_unclassified | Basidiomycota | 0.706 | -4.897 |
| Quercus petraea | Otu00259 | Septoglomus_viscosum | Glomeromycota | 0.712 | -4.934 |
| Quercus petraea | Otu00204 | Montagnulaceae_unclassified | Ascomycota | 0.779 | -5.137 |
| Quercus petraea | Otu00424 | Cryptococcus_heimaeyensis | Basidiomycota | 0.787 | -5.143 |
| Quercus petraea | Otu00282 | Septoglomus_viscosum | Glomeromycota | 0.823 | -5.257 |
| Quercus petraea | Otu00382 | Ophiosphaerella_sp | Ascomycota | 0.816 | -5.264 |
| Quercus petraea | Otu00221 | Geopyxis_unclassified | Ascomycota | 0.991 | -5.303 |
| Quercus petraea | Otu00087 | Paxillus_unclassified | Basidiomycota | 1.614 | -5.305 |
| Quercus petraea | Otu00121 | Rhizophagus_irregularis | Glomeromycota | 1.420 | -5.401 |
| Quercus petraea | Otu00189 | Glomus_sp | Glomeromycota | 1.437 | -5.548 |
| Quercus petraea | Otu00325 | Lasiosphaeriaceae_unclassified | Ascomycota | 0.934 | -5.648 |
| Quercus petraea | Otu00295 | Glomeraceae_sp | Glomeromycota | 0.972 | -5.736 |
| Quercus petraea | Otu00117 | Psathyrella_panaeoloides | Basidiomycota | 1.518 | -5.906 |
| Quercus petraea | Otu00150 | Rhizophagus_irregularis | Glomeromycota | 1.167 | -5.911 |
| Quercus petraea | Otu00257 | Septoglomus_constrictum | Glomeromycota | 1.036 | -5.978 |
| Quercus petraea | Otu00283 | Glomeraceae_unclassified | Glomeromycota | 1.049 | -6.023 |
| Quercus petraea | Otu00275 | Glomeraceae_sp | Glomeromycota | 1.095 | -6.177 |
| Quercus petraea | Otu00220 | Lasiosphaeriaceae_unclassified | Ascomycota | 1.105 | -6.183 |
| Quercus petraea | Otu00212 | Claroideoglomeraceae_unclassified | Glomeromycota | 1.157 | -6.381 |
| Quercus petraea | Otu00050 | Rhizophagus_irregularis | Glomeromycota | 2.233 | -6.396 |
| Quercus petraea | Otu00174 | Claroideoglomus_sp | Glomeromycota | 1.222 | -6.584 |
| Quercus petraea | Otu00085 | Glomeraceae_sp | Glomeromycota | 1.971 | -6.587 |
| Quercus petraea | Otu00230 | Monographella_cucumerina | Ascomycota | 1.231 | -6.607 |
| Quercus petraea | Otu00147 | Septoglomus_viscosum | Glomeromycota | 1.293 | -6.821 |
| Quercus petraea | Otu00202 | Diversisporaceae_sp | Glomeromycota | 1.321 | -6.918 |
| Quercus petraea | Otu00216 | Ceratobasidium_unclassified | Basidiomycota | 1.384 | -7.118 |
| Quercus petraea | Otu00190 | Septoglomus_unclassified | Glomeromycota | 1.421 | -7.249 |
| Quercus petraea | Otu00061 | Pulvinula_sp | Ascomycota | 1.927 | -7.377 |
| Quercus petraea | Otu00182 | Septoglomus_constrictum | Glomeromycota | 1.499 | -7.507 |
| Quercus petraea | Otu00067 | Stephanosporaceae_sp | Basidiomycota | 1.332 | -7.592 |
| Quercus petraea | Otu00109 | Claroideoglomus_sp | Glomeromycota | 1.617 | -7.902 |
| Quercus petraea | Otu00049 | Rhizophagus_irregularis | Glomeromycota | 1.990 | -8.185 |
| Quercus petraea | Otu00092 | Glomus_sp | Glomeromycota | 1.735 | -8.244 |
| Quercus petraea | Otu00024 | Tuber_maculatum | Ascomycota | 1.777 | -9.037 |
| Quercus petraea | Otu00113 | Entoloma_lanicum | Basidiomycota | 1.001 | -19.562 |
| Quercus petraea | Otu00091 | Ascomycota_sp | Ascomycota | 1.657 | -19.897 |
| Quercus petraea | Otu00168 | Glomeraceae_unclassified | Glomeromycota | 1.085 | -20.423 |
| Quercus petraea | Otu00171 | Glomeraceae_sp | Glomeromycota | 1.115 | -20.629 |
| Quercus petraea | Otu00162 | Fungi_sp | unclassified_Fungi | 1.167 | -20.849 |
| Quercus petraea | Otu00058 | Calyptella_sp | Basidiomycota | 1.184 | -20.895 |
| Quercus petraea | Otu00127 | Thielaviopsis_basicola | Ascomycota | 1.283 | -21.028 |
| Quercus petraea | Otu00096 | Tomentella_sp | Basidiomycota | 1.352 | -21.289 |
| Quercus petraea | Otu00053 | Fungi_unclassified | Fungi_unclassified | 1.432 | -21.623 |
| Quercus petraea | Otu00064 | Ascomycota_unclassified | Ascomycota | 1.430 | -21.654 |
| Quercus petraea | Otu00170 | Ceratobasidiaceae_sp | Basidiomycota | 1.640 | -22.219 |
| **OTUs significantly enriched or depleted in Tilia platyphyllos** | |  |  |  |  |
| Tilia platyphyllos | Otu00024 | Tuber_maculatum | Ascomycota | 1.477 | 6.622 |
| Tilia platyphyllos | Otu00221 | Geopyxis_unclassified | Ascomycota | 1.333 | 4.244 |
| Tilia platyphyllos | Otu00094 | Paraphoma_unclassified | Ascomycota | 1.995 | 3.790 |
| Tilia platyphyllos | Otu00271 | Phaeoacremonium_hungaricum | Ascomycota | 1.000 | 3.174 |
| Tilia platyphyllos | Otu00098 | Clonostachys_rosea | Ascomycota | 1.906 | 3.145 |
| Tilia platyphyllos | Otu00287 | Sordariales_unclassified | Ascomycota | 1.051 | 2.928 |
| Tilia platyphyllos | Otu00146 | Mortierella_alpina | Zygomycota | 1.899 | 2.266 |
| Tilia platyphyllos | Otu00493 | Mortierella_unclassified | Zygomycota | 0.482 | 2.213 |
| Tilia platyphyllos | Otu00075 | Fusarium_unclassified | Ascomycota | 2.108 | 1.176 |
| Tilia platyphyllos | Otu00009 | Tetracladium_maxilliforme | Ascomycota | 3.174 | -1.533 |
| Tilia platyphyllos | Otu00011 | Rhizophagus_irregularis | Glomeromycota | 3.116 | -1.883 |
| Tilia platyphyllos | Otu00021 | Rhizophagus_irregularis | Glomeromycota | 2.875 | -2.469 |
| Tilia platyphyllos | Otu00017 | Rhizophagus_irregularis | Glomeromycota | 3.068 | -2.514 |
| Tilia platyphyllos | Otu00044 | Incertae_sedis_unclassified | Basidiomycota | 2.150 | -2.571 |
| Tilia platyphyllos | Otu00052 | Strumella_sp | Ascomycota | 2.329 | -2.579 |
| Tilia platyphyllos | Otu00025 | Cyphellophora_sp | Ascomycota | 2.510 | -2.763 |
| Tilia platyphyllos | Otu00256 | Mortierella_hyalina | Zygomycota | 1.151 | -3.045 |
| Tilia platyphyllos | Otu00019 | Hebeloma_sp | Basidiomycota | 2.527 | -3.047 |
| Tilia platyphyllos | Otu00242 | Ilyonectria_mors-panacis | Ascomycota | 1.082 | -3.052 |
| Tilia platyphyllos | Otu00079 | Talaromyces_aculeatus | Ascomycota | 1.911 | -3.081 |
| Tilia platyphyllos | Otu00185 | Periconia_sp | Ascomycota | 1.559 | -3.144 |
| Tilia platyphyllos | Otu00041 | Ascomycota_unclassified | Ascomycota | 2.404 | -3.565 |
| Tilia platyphyllos | Otu00249 | Schizothecium_glutinans | Ascomycota | 1.292 | -3.874 |
| Tilia platyphyllos | Otu00182 | Septoglomus_constrictum | Glomeromycota | 1.499 | -3.899 |
| Tilia platyphyllos | Otu00275 | Glomeraceae_sp | Glomeromycota | 1.095 | -3.912 |
| Tilia platyphyllos | Otu00085 | Glomeraceae_sp | Glomeromycota | 1.971 | -3.940 |
| Tilia platyphyllos | Otu00343 | Guehomyces_pullulans | Basidiomycota | 0.509 | -4.007 |
| Tilia platyphyllos | Otu00358 | Pulvinula_sp | Ascomycota | 0.512 | -4.044 |
| Tilia platyphyllos | Otu00386 | Basidiomycota_unclassified | Basidiomycota | 0.565 | -4.197 |
| Tilia platyphyllos | Otu00144 | Chalastospora_ellipsoidea | Ascomycota | 0.875 | -4.426 |
| Tilia platyphyllos | Otu00090 | Agaricales_unclassified | Basidiomycota | 1.679 | -4.600 |
| Tilia platyphyllos | Otu00104 | Pezizaceae_sp | Ascomycota | 0.959 | -4.701 |
| Tilia platyphyllos | Otu00202 | Diversisporaceae_sp | Glomeromycota | 1.317 | -4.785 |
| Tilia platyphyllos | Otu00117 | Psathyrella_panaeoloides | Basidiomycota | 1.374 | -4.923 |
| Tilia platyphyllos | Otu00192 | Incertae_sedis_unclassified | Ascomycota | 0.943 | -5.039 |
| Tilia platyphyllos | Otu00027 | Agaricales_unclassified | Basidiomycota | 2.443 | -5.069 |
| Tilia platyphyllos | Otu00218 | Glomus_sp | Glomeromycota | 1.263 | -5.086 |
| Tilia platyphyllos | Otu00130 | Mycosphaerella_tassiana | Ascomycota | 1.429 | -5.285 |
| Tilia platyphyllos | Otu00150 | Rhizophagus_irregularis | Glomeromycota | 1.167 | -5.392 |
| Tilia platyphyllos | Otu00147 | Septoglomus_viscosum | Glomeromycota | 1.290 | -5.649 |
| Tilia platyphyllos | Otu00220 | Lasiosphaeriaceae_unclassified | Ascomycota | 1.105 | -5.919 |
| Tilia platyphyllos | Otu00153 | Ilyonectria_robusta | Ascomycota | 1.115 | -5.970 |
| Tilia platyphyllos | Otu00063 | Rhizophagus_unclassified | Glomeromycota | 1.582 | -6.276 |
| Tilia platyphyllos | Otu00061 | Pulvinula_sp | Ascomycota | 1.927 | -6.414 |
| Tilia platyphyllos | Otu00071 | Auriculariales_unclassified | Basidiomycota | 1.571 | -7.046 |
| Tilia platyphyllos | Otu00096 | Tomentella_sp | Basidiomycota | 1.352 | -7.213 |
| Tilia platyphyllos | Otu00058 | Calyptella_sp | Basidiomycota | 1.184 | -7.415 |
| Tilia platyphyllos | Otu00067 | Stephanosporaceae_sp | Basidiomycota | 1.332 | -7.580 |
| Tilia platyphyllos | Otu00032 | Tuber_maculatum | Ascomycota | 1.613 | -8.177 |
| Tilia platyphyllos | Otu00040 | Flagelloscypha_minutissima | Basidiomycota | 2.256 | -10.570 |
| Tilia platyphyllos | Otu00170 | Ceratobasidiaceae_sp | Basidiomycota | 1.589 | -18.034 |
| Tilia platyphyllos | Otu00113 | Entoloma_lanicum | Basidiomycota | 0.917 | -20.199 |
| Tilia platyphyllos | Otu00102 | Tomentella_ellisii | Basidiomycota | 1.097 | -20.763 |
| Tilia platyphyllos | Otu00118 | Glarea_unclassified | Ascomycota | 1.182 | -21.035 |
| Tilia platyphyllos | Otu00064 | Ascomycota_unclassified | Ascomycota | 1.430 | -21.829 |
| Tilia platyphyllos | Otu00086 | Subulicystidium_sp | Basidiomycota | 1.578 | -22.301 |

Appendix Table S4: Linear mixed effects models relating mycorrhization rates assessed by morphological methods as well as OTU richness and ACE to log_2_ tree species richness and mycorrhiza host type (Myc_Type). Shown are estimates (est) and standard errors (se) of marginal effects of fixed factors as well as p values based on a type III Anova, with significant p values given in bold fonts.

|  | log(R)=0_AM | | log(R)=0_EM | | log(R)=1_AM | | log(R)=1_EM | | log(R)=2_AM | | log(R)=2_EM | | log Tree richness | Myc_Type | log Tree richness: Myc_Type |
| --- | --- | --- | --- | --- | --- | --- | --- | --- | --- | --- | --- | --- | --- | --- | --- |
|  | est | se | est | se | est | se | est | se | est | se | est | se | p value | p value | p value |
| ECT | 53.66 | 8.05 | 44.96 | 8.06 | 49.11 | 4.25 | 44.94 | 4.25 | 44.56 | 5.17 | 44.91 | 5.08 | 0.5581 | 0.4447 | 0.5346 |
| AM F | 84.22 | 7.45 | 27.54 | 7.24 | 85.78 | 4.09 | 31.33 | 4.00 | 87.34 | 5.05 | 35.11 | 4.83 | 0.4303 | **<0.0001** | 0.7428 |
| AM M | 11.05 | 2.92 | 3.04 | 2.85 | 11.17 | 1.68 | 3.26 | 1.65 | 11.30 | 1.97 | 3.47 | 1.89 | 0.8953 | 0.0762 | 0.9710 |
| AM A | 0.97 | 0.58 | 0.95 | 0.56 | 0.76 | 0.36 | 0.60 | 0.35 | 0.55 | 0.40 | 0.25 | 0.38 | 0.2511 | 0.9737 | 0.7703 |
| OTU richness all fungi | 392.43 | 50.13 | 332.18 | 50.28 | 391.27 | 41.67 | 342.06 | 41.62 | 390.11 | 42.41 | 351.94 | 41.96 | 0.7629 | 0.4088 | 0.7024 |
| ACE all fungi | 901.93 | 105.05 | 708.39 | 105.40 | 856.68 | 81.51 | 796.51 | 81.38 | 811.43 | 84.08 | 884.64 | 82.76 | 0.5358 | 0.2083 | 0.0558 |
| OTU richness Ascomycota | 176.19 | 18.54 | 157.13 | 18.62 | 174.77 | 13.99 | 161.18 | 13.97 | 173.35 | 14.74 | 165.24 | 14.55 | 0.8434 | 0.4732 | 0.6699 |
| ACE Ascomycota | 370.83 | 79.80 | 433.79 | 80.27 | 360.04 | 60.56 | 428.03 | 60.53 | 349.26 | 62.69 | 422.26 | 61.95 | 0.7696 | 0.5830 | 0.9263 |
| OTU richness Basidiomycota | 62.92 | 35.96 | 82.79 | 36.07 | 63.35 | 31.10 | 98.07 | 31.07 | 63.78 | 31.48 | 113.35 | 31.25 | 0.4091 | 0.7027 | 0.4305 |
| ACE Basidiomycota | 168.30 | 79.88 | 144.02 | 80.37 | 153.47 | 61.01 | 237.10 | 60.99 | 138.64 | 63.08 | 330.19 | 62.39 | 0.1663 | 0.8317 | **0.0480** |
| OTU richness Glomeromycota | 116.34 | 15.44 | 26.19 | 15.58 | 103.68 | 11.67 | 26.56 | 11.69 | 91.01 | 12.11 | 26.93 | 12.02 | 0.2888 | **0.0004** | 0.2036 |
| ACE Glomeromycota | 370.65 | 37.32 | 41.28 | 38.37 | 325.17 | 23.43 | 49.75 | 24.13 | 279.68 | 26.43 | 58.22 | 26.61 | 0.2642 | **<0.0001** | 0.1048 |

log(R): log_2_ of plot tree species richness (which is 0, 1 and 2 for plot richness 1, 2 and 4 species, respectively). Myc_Type: The tree species’ preferred type of mycorrhiza (AM, arbuscular mycorrhiza; EM ectomycorrhiza). ECT: Frequency of active ectomycorrhizal root tips. AM F: Frequency of arbuscular mycorrhiza in the root system. AM M: Intensity of the arbuscular mycorrhizal colonisation in the root system. AM A: Arbuscular abundance in the root system. OTU richness and ACE (abundance-based coverage estimator) of all fungi and separately by phylum (Ascomycota, Basidiomycota and Glomeromycota).


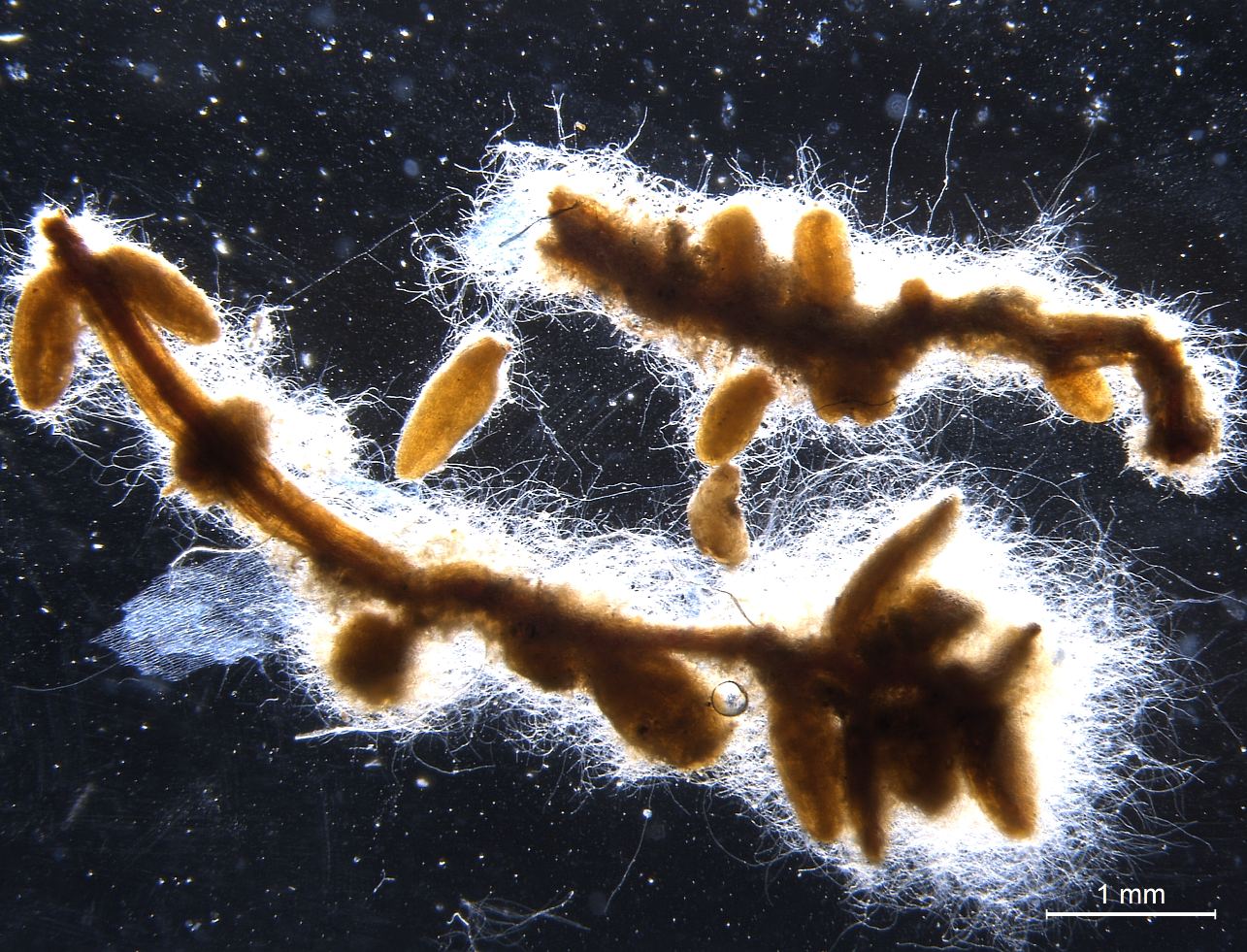

Appendix Figure S1: Fine roots of *Aesculus hippocastanum* (top) and a cross section of a cone-shaped fine root, with the fungal structures stained blue (bottom). There is no hyphal mantle in the outer layers of the root parenchyma and no Hartig net. Photographs: Paul Kühn.


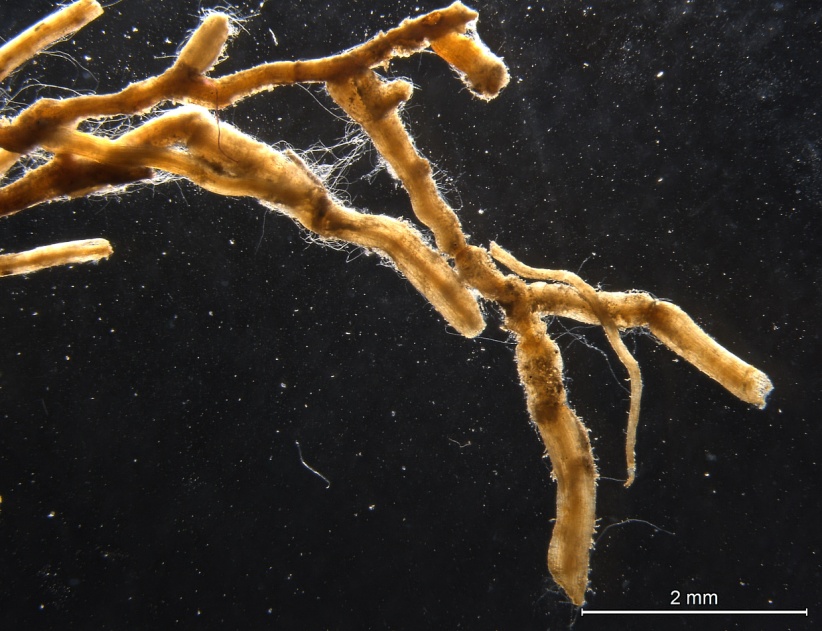

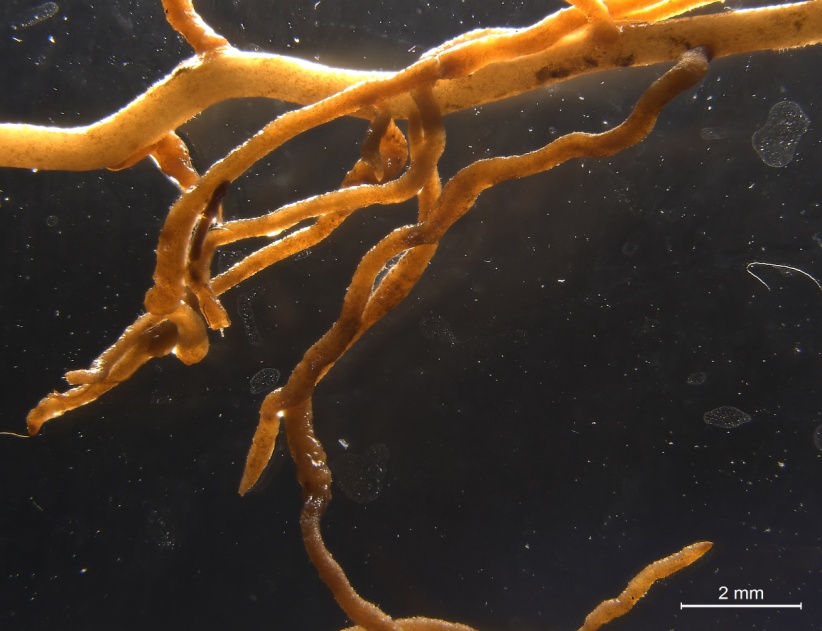

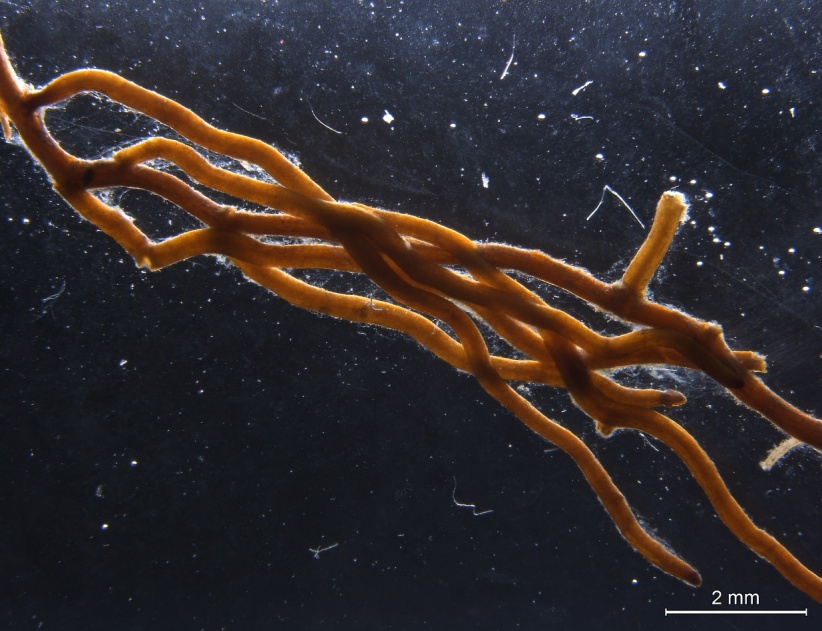

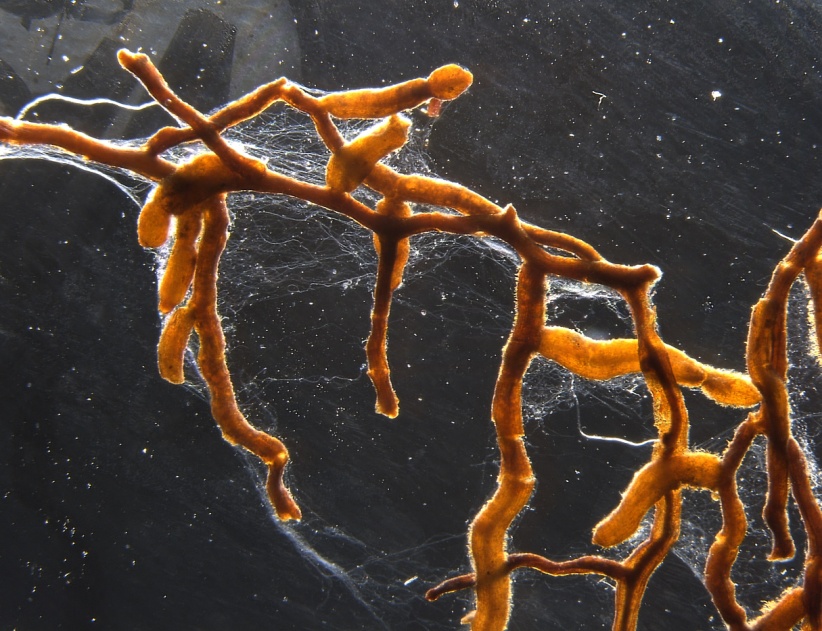


a

b

c

d

Appendix Figure S2: Fine roots of AM tree species from the MyDiv experiment. a) *Acer pseudoplatanus,* b) *Fraxinus excelsior,* c) *Prunus avium,* d) *Sorbus aucuparia,* showing EM mycorrhization, as revealed by by lighter-coloured and swelled root tips of fresh roots. Photographs: Paul Kühn.


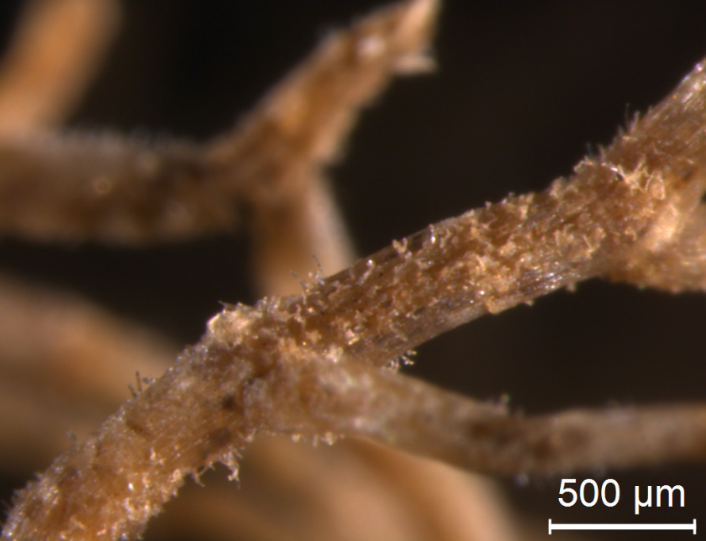

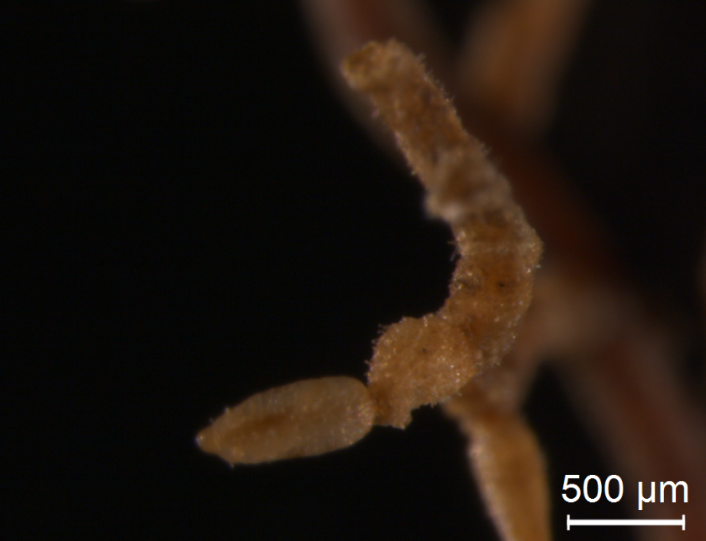

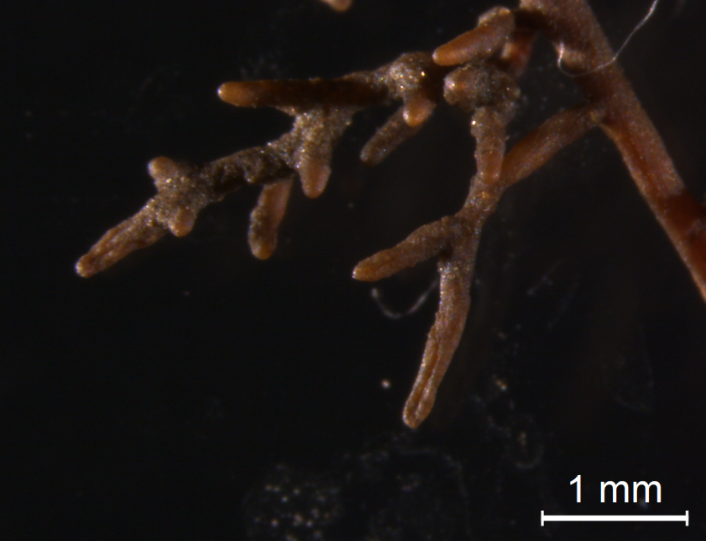

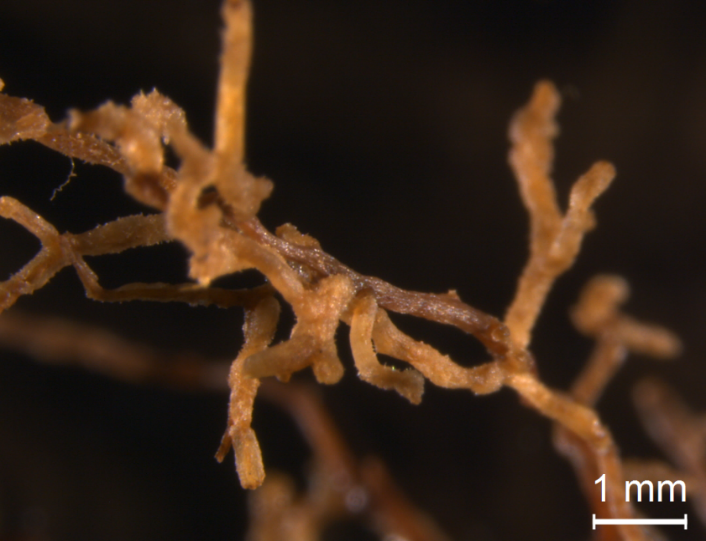


a

b

c

d

Appendix Figure S3: Fine roots of EM tree species from the MyDiv experiment. a) *Betula pendula,* b) *Fagus sylvatica,* c) *Carpinus betulus,* d) *Tilia platyphyllos,* showing EM mycorrhization, as revealed by by lighter-coloured and swelled root tips of fresh roots. Photographs: Nicole Schindler.


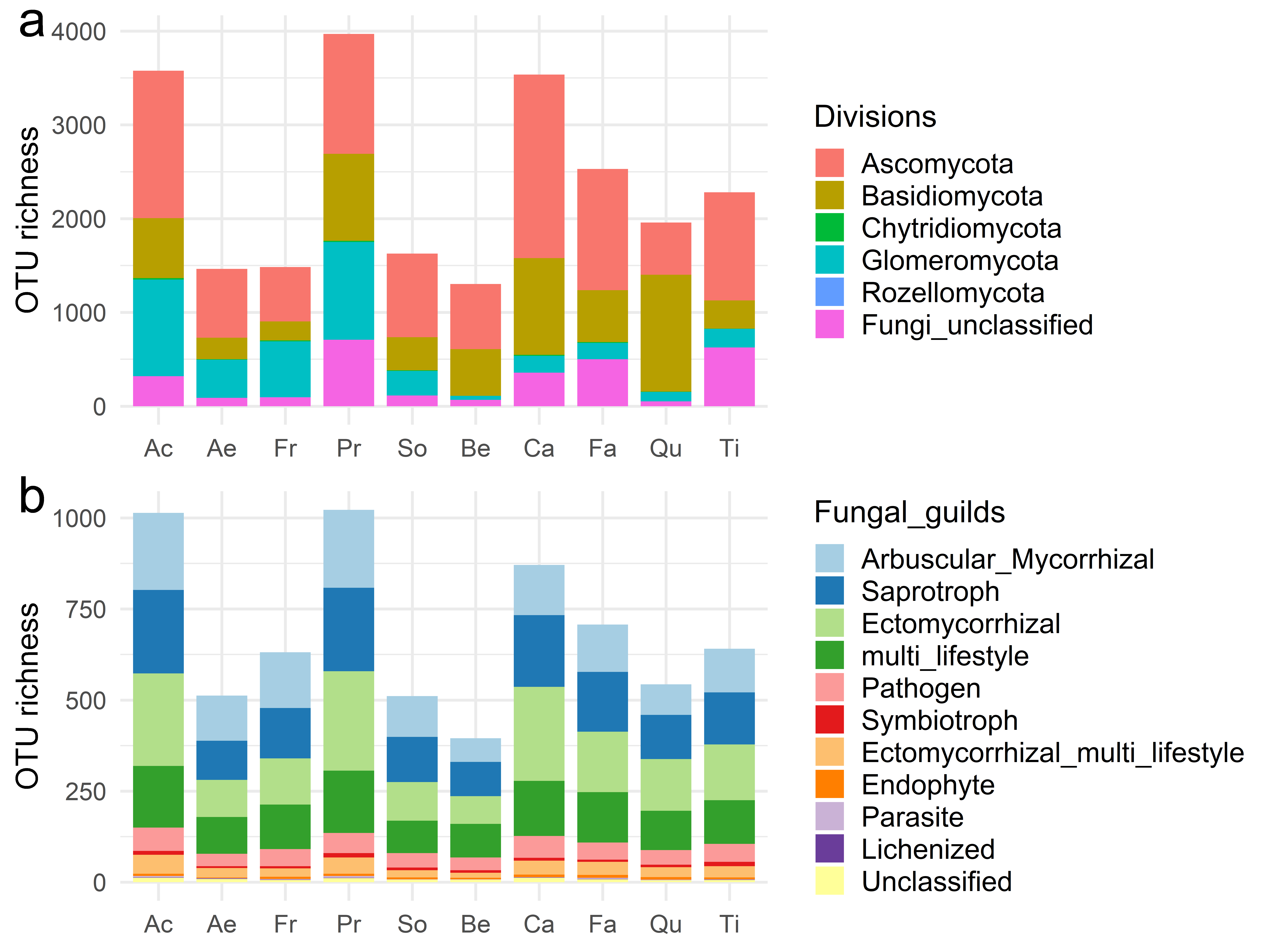


Appendix Figure S4: OTU richness across the whole study shown a) by fungal divisions and b) by fungal guild, separately by host tree species. Host tree species on the left site are AMF tree species (Ac=*Acer pseudoplatanus,* Ae=*Aesculus hippocastanum*, Fr=*Fraxinus excelsior,* Pr=*Prunus avium,* So=*Sorbus aucuparia*), while those on the right side are EMF species (Be=*Betula pendula,* Ca=*Carpinus betulus,* Fa=*Fagus sylvatica,* Qu*=Quercus petraea,* Ti= *Tilia platyphyllos).* a) is based on the total number of 15,366 OTUs detected across all samples, while b) refers to the 1,996 OTUs listed in FUNGuild (Nguyen et al., 2016). All guilds containing more than one lifestyle were pooled in the group of “multi-lifestyle”.


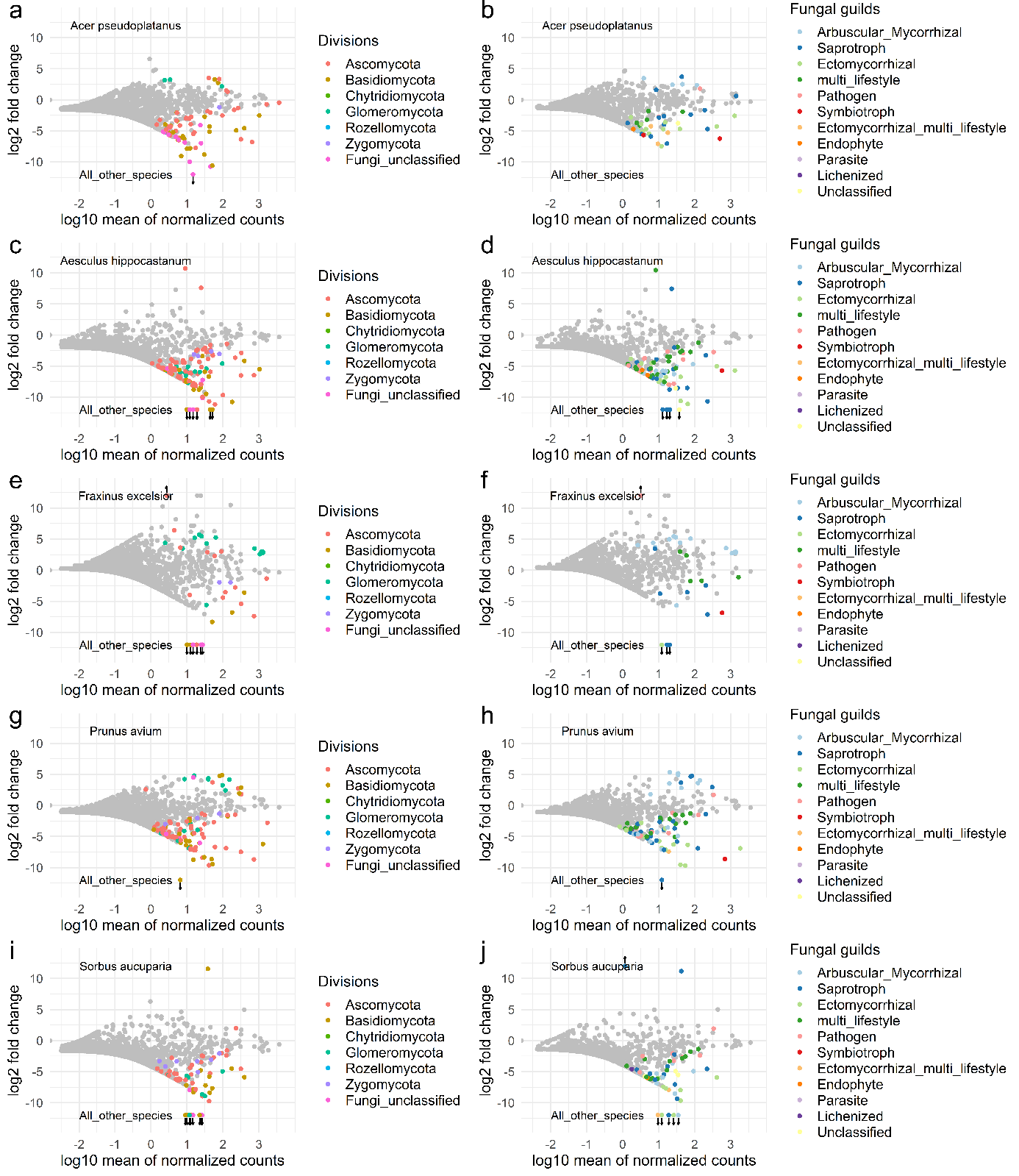
Appendix Figure S5: OTUs enriched in particular tree species typically associated with AM as opposed to all other species plotted against abundance (log_10_ mean of normalized counts). a), b) *Acer pseudoplatanus*, c), d) *Aesculus hippocastanum*, e), f) *Fraxinus excelsior*, g), h) *Prunus avium*, i), j) *Sorbus aucuparia*. Insignificant fold changes are shown in grey, while significant fold change according to a Wald test (p < 0.05) are highlighted in colour. a), c), e), g) and i) significant fold changes by taxonomic division, based on all 15,366 OTUs detected across all samples. b), d), f), h) and j) significant fold changes by taxonomic fungal guilds, based on the 1,996 OTUs listed in FUNGuild (Nguyen et al., 2016).


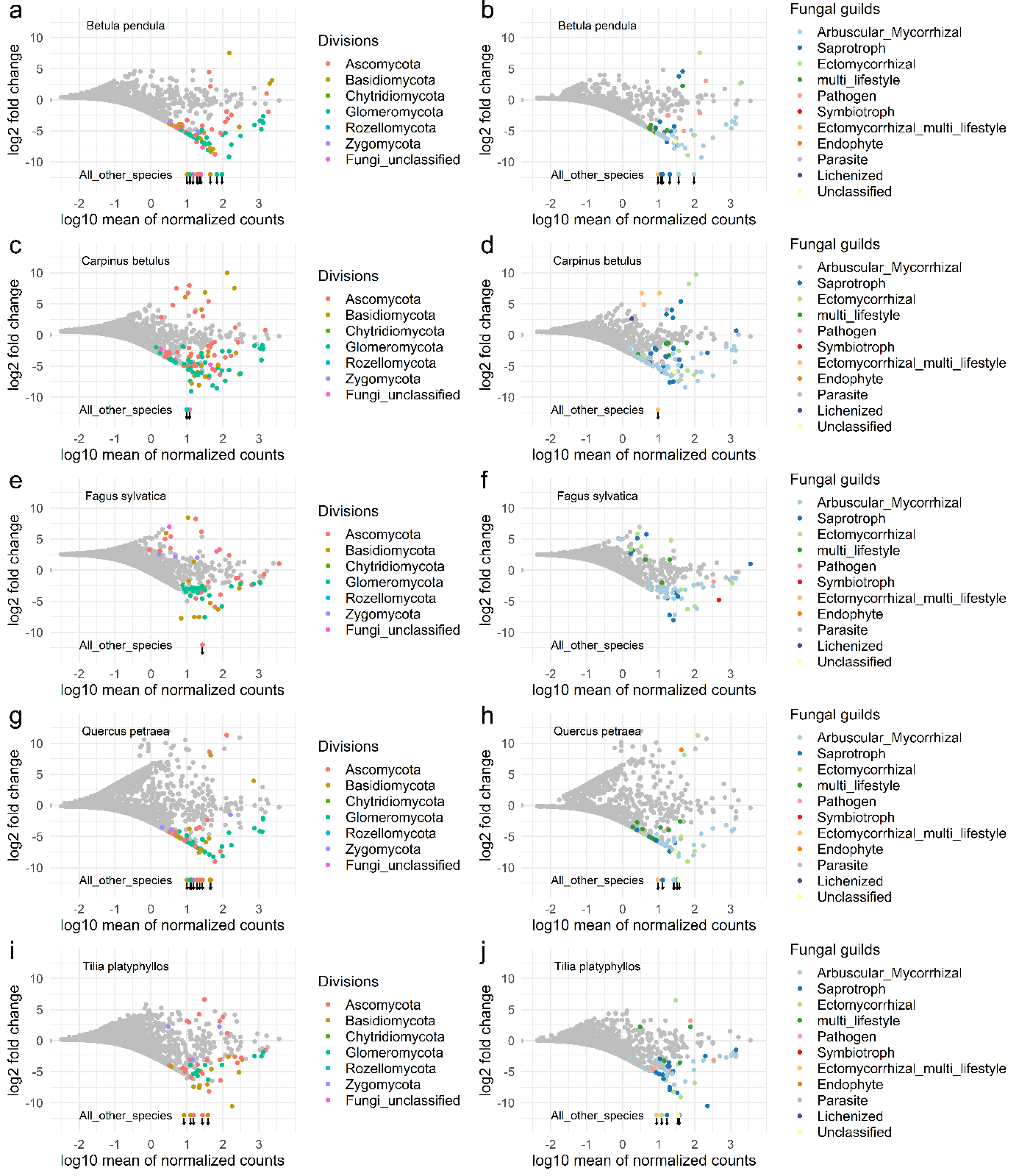


Appendix Figure S6: OTUs enriched in particular tree species typically associated with EM as opposed to all other species plotted against abundance (log_10_ mean of normalized counts). a), b) *Betula pendula*, c), d) *Carpinus betulus*, e), f) *Fagus sylvatica*, g), h) *Quercus petraea*, i), j) *Tilia platyphyllos*. Insignificant fold changes are shown in grey, while significant fold change according to a Wald test (p < 0.05) are highlighted in colour. a), c), e), g) and i) significant fold changes by taxonomic division, based on all 15,366 OTUs detected across all samples. b), d), f), h) and j) significant fold changes by taxonomic fungal guilds, based on the 1,996 OTUs listed in FUNGuild (Nguyen et al., 2016).


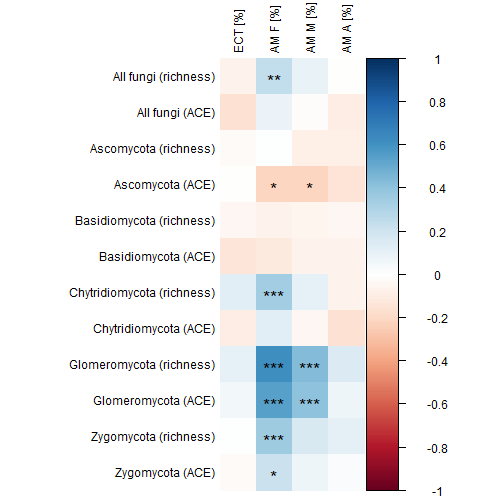


Appendix Figure S7: Spearman correlation between mycorrhization rates assessed by microscopy (columns) and molecular data (rows) for OTU richness and abundance-based coverage estimator (ACE) as obtained from next-generation sequencing, shown separately by phylum. *** p< 0.001, ** p < 0.01, * p < 0.05.
